# An ECG biomarker for sudden cardiac death discovered with deep learning

**DOI:** 10.1038/s41586-026-10674-6

**Published:** 2026-06-24

**Authors:** Ziad Obermeyer, Alexander Schubert, James Ross, Sendhil Mullainathan, Markus Lingman

**Affiliations:** 1 School of Public Health, University of California, Berkeley, Berkeley, CA USA; 2 Computational Precision Health, College of Computing, Data Science, and Society, University of California, Berkeley, Berkeley, CA USA; 3 Booth School of Business, University of Chicago, Chicago, IL USA; 4 Department of Economics, MIT, Cambridge, MA USA; 5 Department of Electrical Engineering and Computer Science, MIT, Cambridge, MA USA; 6 Center for Applied Intelligent Systems Research in Health, Halmstad University & Region Halland, Halmstad, Sweden; 7 Institute of Medicine, Department of Molecular and Clinical Medicine, University of Gothenburg & Sahlgrenska University Hospital, Gothenburg, Sweden

**Keywords:** Predictive markers, Translational research

## Abstract

Sudden cardiac death is, in theory, preventable with defibrillators. But every year, many patients die without defibrillators because doctors fail to predict their risk^1^. The only predictive biomarker in wide use, cardiac left ventricular ejection fraction (LVEF), misses most sudden cardiac deaths^2^, and flags many low-risk patients for futile defibrillators that never fire^3,4^. Here we apply deep learning to a dataset linking all electrocardiograms (ECGs) in a Swedish region to death certificates. The resulting model isolates a high-risk group (2.2% of the sample) with a 7.0% annual rate of sudden cardiac death, higher than those with reduced LVEF (1.9% of the sample; 4.6% annual rate). Notably, 86.1% of the model’s high-risk patients were not flagged by LVEF. High-risk ECG patients with defibrillators implanted were 54.4% less likely to die than expected, suggesting a mortality benefit. We externally validate the model in a US health system, in which it predicts ventricular arrhythmias that cause sudden death; and a Taiwanese hospital registry, in which it specifically predicts future arrhythmic cardiac arrests. To visualize the waveform morphology ‘discovered’ by the predictive model, we pair it with a generative model of the ECG waveform. Together, they reveal a biomarker that is easily visible and robustly predicts sudden cardiac death, but has not to our knowledge been previously described. Tying the biomarker’s shape to electrophysiological first principles, we form and preliminarily test a new hypothesis on the mechanism of sudden cardiac death.

## Abstract (web summary)

A deep-learning model trained on electrocardiogram (ECG) waveforms identifies an easily visible biomarker that predicts sudden cardiac death more accurately than the current clinical state of the art.

## Main

Every year, cardiac arrhythmias cause hundreds of thousands of sudden deaths in the USA alone^1,5^. These deaths occur despite the availability—since 1980—of implanted cardioverter defibrillators, which can detect and terminate arrhythmias before they kill.

Implanting a defibrillator has costs, so choosing the right patients requires risk prediction: the higher the likelihood of future arrhythmia, the more the benefits outweigh the costs^6–9^. The current state of the art for risk prediction is a biomarker measured through cardiac ultrasound: the heart’s left ventricular ejection fraction (LVEF)^9,10^. Reduced LVEF confers a high risk of sudden cardiac death, and a large survival benefit from defibrillators^1,11^. But LVEF has many false negatives: most people who suffer sudden cardiac death do not have LVEF measured premortem, and of those who do, only a minority have reduced LVEF^2^. LVEF also has false positives: of all defibrillators implanted for reduced LVEF, two-thirds never go on to deliver a life-saving shock, incurring all the costs of implantation without any of the benefits^3,4^.

A growing awareness of LVEF’s shortcomings has prompted many to call for new approaches^12–16^, but LVEF remains the only predictor in widespread use. This has as much to do with its pragmatic advantages as its predictive power: LVEF is an imaging-based biomarker, and the ultrasounds that measure it are ubiquitously available and highly standardized around the world (minor inter-observer variation notwithstanding)^17,18^. Although other diagnostic modalities have shown promise—for example, cardiac magnetic resonance imaging (cMRI), long-term ambulatory monitoring, electrophysiological studies, positron emission tomography (PET), single-photon emission computed tomography (SPECT) and genetic profiling^5,9,18,19^—these costly and sometimes risky tests are impractical for screening large populations. At the other end of the cost spectrum, ECGs have long been scrutinized for predictive biomarkers, but none has achieved sufficient predictive power to compete with LVEF^10,20^. In addition, although ECGs are cheap, coding ECG biomarkers requires human expertise, making data collection costly and variable and limiting generalizability.

Deep learning applied to ECGs could provide the best of both worlds: a costless, widely available, standardized image that can be fed directly into a classifier, predicting risk without human input. A key reason such classifiers do not yet exist is the scarcity of large training datasets of ECGs linked to reliable information on cause of death. Although impressive progress has been made with small case–control datasets^21^, it is unclear whether such models can generalize to the goal of screening populations.

Here we train a deep-learning model using population-based data from Sweden, linking the universe of ECG waveforms from an entire region to death certificates and electronic health records. We evaluate the model’s ability to predict sudden cardiac death, and the arrhythmias that cause it, in an independent Swedish hold-out set, and compare it with LVEF. We also test the model’s ability to generalize to two diverse datasets from the USA and Taiwan. Finally, to visualize the biomarker identified by the model, we train a generative model to create synthetic high-risk ECGs.

## Predictive performance in the Sweden data lockbox

Sudden cardiac death is, by construction, mysterious: when patients die suddenly outside the hospital, it is difficult to know what happened. Recognizing the inevitable uncertainty in any one definition of sudden cardiac death, we evaluate our model against a diverse set of definitions from the literature: death certificates, diagnoses of arrhythmias that cause sudden death and detailed chart review.

We first evaluate model predictions against the most straightforward definition, which was also the training label for our model: a death certificate indicating out-of-hospital death from cardiac causes, in the year after an ECG. Our main analyses are performed in a 40% data ‘lockbox’: 119,541 ECGs with one-year follow-up, from 35,885 patients under 80 years old, and specifically the subset of 113,072 ECGs from 35,417 patients without defibrillators. The lockbox was created and set aside before any data analysis or model training, and was not touched until after the provisional acceptance of this manuscript at *Nature*. In the originally submitted version, our model was trained on a 30% random sample, and validated in another 30%. On provisional acceptance, we retrained the model on all 60% of patients whose data we had accessed (262,554 ECGs from 75,157 patients), and without further modification, produced results for the lockbox. This sample is described in Table 1, and changes from the original to the present manuscript are shown in Supplementary Information section VII.A.

**Table 1:** Summary statistics for the Sweden data lockbox (under 80 years old)

	All	By outcome	
		**Sudden cardiac death**^**a**^	**All others**
Patients	35,885	143	35,812
ECGs, *n*	119,541	711	118,830
ECG count per patient, mean	3.33	4.97	3.32
Median days from ECG to end of follow-up period (25th–75th percentile)	2,100 (1,164–2,760)	111 (40–215)	2,100 (1,170–2,760)
**Demographics**			
Age, mean	59.3 (0.0502)	69.2 (0.3144)	59.3 (0.0504)
Female	0.450 (0.0014)	0.323 (0.0175)	0.451 (0.0014)
Sudden cardiac death, year after ECG	0.006 (0.0002)	1	0
VF/VT, year after ECG	0.013 (0.0003)	0.027 (0.0060)	0.012 (0.0003)
Defibrillator implanted before ECG	0.054 (0.0007)	0.077 (0.0100)	0.054 (0.0007)
**SCD risk factors**^**b**^			
Prior VF/VT	0.026 (0.0005)	0.110 (0.0117)	0.026 (0.0005)
LVEF recorded^c^	0.243 (0.0012)	0.439 (0.0186)	0.242 (0.0012)
LVEF ≤ 35%	0.026 (0.0005)	0.160 (0.0138)	0.025 (0.0005)
CHF and cardiomyopathy	0.074 (0.0008)	0.464 (0.0187)	0.072 (0.0008)
Recent MI			
Acute MI diagnosis (40 days before ECG)	0.033 (0.0005)	0.100 (0.0112)	0.032 (0.0005)
Positive troponin (40 days before ECG)^d^	0.120 (0.0009)	0.453 (0.0187)	0.118 (0.0009)
CAD history	0.131 (0.0010)	0.304 (0.0172)	0.130 (0.0010)
Previous MI	0.054 (0.0007)	0.156 (0.0136)	0.053 (0.0007)
Hypertension	0.210 (0.0012)	0.522 (0.0187)	0.208 (0.0012)
Hyperlipidaemia	0.036 (0.0005)	0.084 (0.0104)	0.036 (0.0005)
Diabetes	0.117 (0.0009)	0.276 (0.0168)	0.116 (0.0009)

Numbers are fractions (with s.e. clustered by patient in parentheses) unless noted. Statistics are calculated at ECG level. Note that this table includes patients with defibrillators, so numbers may differ slightly from those in the text when calculated in the sample without defibrillators. CAD, coronary artery disease; CHF, congestive heart failure; MI, myocardial infarction.

^a^Sudden cardiac death classification is based on death certificates in the year after an ECG, so the same patient may appear in both this and the next column.

^b^Diagnoses, defibrillator placement and cardiac risk factors come from International Classification of Disease codes in electronic records; see Supplementary Information section VII.B.

^c^LVEF comes from cardiac ultrasound results, using the study closest in time to each ECG (median: 51.8 days).

^d^Positive troponin is based on sex-specific thresholds (≥34 ng l^−1^ male; ≥16 ng l^−1^ female).

We calculate discrimination using the area under the receiver-operating-characteristic curve (AUC): 0.872 (patient-level bootstrapped 95% confidence interval (CI): 0.843–0.899). For additional performance metrics and calibration, receiver operating characteristic and precision-recall curves (and AUPRC), overall and in clinically relevant subgroups, see Supplementary Information section I.A. By comparison, generic predictors of cardiovascular risk have a lower AUC: 0.697 for the American Heart Association–American College of Cardiology (AHA/ACC) 10-year risk score^22^, and 0.655 for a validated ECG-based deep-learning model for heart disease (SEER^23^). Our model also has the highest AUC in clinically relevant subgroups (Supplementary Information section I.B).

**Fig. 1:**
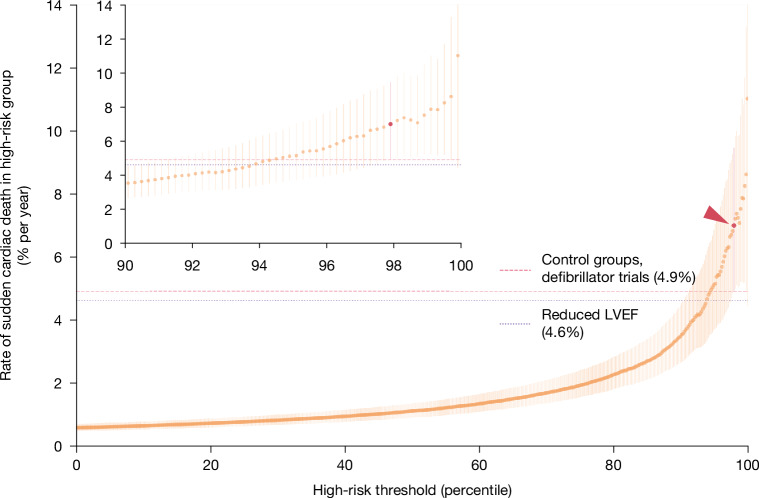
Positive predictive value for sudden cardiac death. Rate of sudden cardiac death (from death certificates) in the high-risk group (*y* axis, with bootstrapped 95% CI), versus percentile threshold used to define high-risk group (*x* axis); inset zooms in on the top 10%. Upper CI in the highest-risk group is censored by top of* y* axis. Horizontal lines show median outcome rate in defibrillator trial control groups and in patients with reduced LVEF. Dark-red point (arrowhead) shows our preferred high-risk group, where lower-bound risk equals median RCT control group patient risk. Total *n* = 113,072 ECGs.

To be clinically useful, predictions must flag a discrete group whose annual risk is high enough to benefit from defibrillators. This requires us to choose a risk threshold. Figure 1 shows sudden cardiac death rate from death certificates, in a range of high-risk groups defined by varying the threshold over model-predicted risk percentiles. This is analogous to positive predictive value (PPV). At the far right is the annual rate in the highest-risk 0.2%: 11.0% (95% CI: 4.4%–17.0%). Moving left, the threshold decreases and the next riskiest ECGs are added to the high-risk group. Sudden cardiac death risk increases sharply above the 90th risk percentile. The riskiest 1.0% have the highest 95% CI lower bound, with an annual rate of 7.9% (95% CI: 5.2%–11.0%).

Our preferred high-risk group comprises 2.2% of the sample, and has an annual sudden cardiac death rate of 7.0% (95% CI: 4.9–9.5%; survival analysis in Supplementary Information section I.C). We set this threshold on the basis of data from randomized trials of defibrillators, which provide implicit guidance on the risk level that justifies defibrillator placement (in the absence of formal guidelines^1^). To enrol patients ethically, risk must be high enough to justify a defibrillator in equipoise. That risk is approximated by the rate of sudden cardiac death in the trial control groups, reported in six major trials (for example, MUSTT, DEFINITE, and DINAMITE; see Supplementary Information section II). Our high-risk group is the largest group whose lower 95% confidence bound exceeds the median control group rate (4.9%; dark red in Fig. 1), such that our high-risk patients are riskier than the median control patient who could have received a defibrillator.

One concern with death certificates is that they might mistakenly flag sudden non-arrhythmic deaths (for example, from stroke, pulmonary failure and so on) as arrhythmic^24–28^. A model trained on death certificates might then conflate these causes in its high-risk group, and thus flag some deaths that are not preventable with defibrillators.

**Fig. 2:**
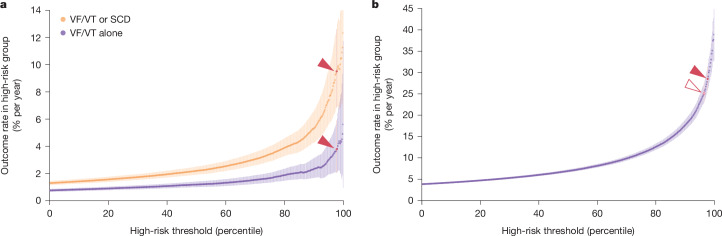
Incidence of ventricular arrhythmia in the high-risk group versus the threshold for defining the high-risk group. **a**, Swedish data. Rate of VF/VT combined with sudden cardiac death (SCD, from death certificates; *n* = 113,072 ECGs; orange) and rate of VF/VT alone (*n* = 106,890 ECGs after accounting for censoring by death; purple). **b**, US data. Rate of VF/VT (*n* = 251,858 ECGs; purple). Both panels show rates and 95% CI in the high-risk group (*y* axis) versus the percentile threshold used to define the high-risk group (*x* axis). Dark-red point (solid arrowhead) shows our preferred high-risk group, comprising the riskiest 2.2% of the sample; open dark-red point (open arrowhead) shows an alternative definition of the high-risk group in the USA, using the absolute high-risk threshold from Sweden.

To investigate, we complement the analysis above by testing the model’s ability to predict the proximal causes of arrhythmic death, ventricular fibrillation or ventricular tachycardia (VF/VT), documented in health records. These rhythms can be seen in patients who do not die, and correlate with future mortality (see Box 1 for an illustrative case study). Of note, not all incident VF/VT is diagnosed: these rhythms are transient, either because they self-resolve or because they kill the patient. We thus show the composite rate of VF/VT or sudden cardiac death in Fig. 2a, as well as rates of VF/VT alone. Both increase monotonically in risk (we account for the censoring of VF/VT in high-risk patients by death; further discussion in Supplementary Information section III.C). Over and above the 7.0% of the high-risk group with sudden cardiac death, an additional 3.8% per year (95% CI: 2.2–7.0) have VF/VT.

Box 1 Case study of a high-risk patient in Sweden Some details have been changed to protect privacy. A 75-year-old woman with a remote history of lymphoma presented to a regional ED after several episodes of lightheadedness. She was found to be in intermittent VT and admitted to the hospital. She underwent cardiac ultrasound, which showed normal LVEF (50%), and cardiac catheterization, which showed clean coronary arteries. After discharge, she was referred to the electrophysiology service for further testing. Her VT could be provoked in the catheterization laboratory, but no clear source was identified, and she was scheduled for outpatient defibrillator placement. But she experienced sudden cardiac death before her procedure. An ECG, done at a routine outpatient visit four months before her ED visit, would have flagged her as high risk on the basis of our model predictions.

## Model generalization to US and Taiwan data

We next investigate how model predictions generalize to two external datasets. It is important to acknowledge that our datasets differ structurally, in terms of design and outcome definitions: in Sweden we use both death certificates and VF/VT diagnoses from health records; in the USA, VF/VT diagnoses only; and in Taiwan, a case–control hospital registry dataset capturing arrhythmic arrests. This affects interpretation of performance metrics, which we interrogate and discuss further in Supplementary Information section III.A-C. That said, a benefit of these differences is that each dataset highlights a different aspect of model performance across diverse global populations, and different measurement strategies for sudden cardiac death.

The first dataset is drawn from the health records of a large non-academic US health system (Sharp HealthCare, San Diego, CA): all 251,858 ECGs performed between 2021 and 2022 in 139,613 patients under the age of 80 (see Supplementary Information section VII.C for more details). We do not observe the cause of death, because of difficulties in linking US health records to death certificates. However, we do observe the incidence of VF/VT in the year after ECGs, from longitudinal records. In the USA, because there are strong financial incentives for measuring and documenting VF/VT, a higher fraction of incident arrhythmias is likely to be captured. Although this introduces clear differences in the nature of the VF/VT label in the USA versus Sweden, it has a compelling advantage: in Sweden, a model that accurately predicts a patient’s VF/VT might appear ‘wrong’ if the patient goes untested (that is, a false negative). In the USA, more intensive measurement of incident VF/VT will accurately label such a patient, providing a useful complement to Sweden.

With no fine-tuning or other modification, the model ‘zero-shot’ AUC for VF/VT is 0.822 (95% CI: 0.812–0.831) in the USA, compared with 0.717 (95% CI: 0.676–0.756) in Sweden. Figure 2b shows annual VF/VT increases sharply in predicted risk. Using a similarly sized high-risk group to that in Sweden (riskiest 2.2%, solid dark-red point and arrow), the incidence of VF/VT is 29.1% (95% CI: 26.5–31.9%; versus base rate of 3.8%). If we set the threshold using the same absolute risk threshold as Sweden, which requires no distributional information about the US population, the high-risk group is larger—3.4% of the sample (hollow dark-red point and arrow)—with a VF/VT incidence of 25.7% (95% CI: 23.6– 28.0%). Supplementary Information section III.C shows that, conditional on model-predicted risk, patients in the USA are six times more likely to be coded as having VF/VT than are those in Sweden, supporting the idea that better performance in the USA than in Sweden might reflect more accurate labelling and intensive measurement. In addition, lower barriers to access and better overall health in Sweden could compress the distribution of cardiovascular risk, making it harder for the model to distinguish high-risk patients from others; a wider risk distribution in the USA would make this task easier. Supporting this, we find that the US risk distribution is both higher on average (mean: 0.64% versus 0.38%), and wider (s.d.: 1.85% versus 1.23%); details in Supplementary Information section III.C.

Our second dataset is a hospital-based registry in Taiwan (National Taiwan University Hospital, Taipei), where we identify 257 emergency department (ED) patients under 80 years old in cardiac arrest (that is, without measurable cardiac output), as well as a random sample of 4,011 under-80-year-old control patients visiting the same ED (see Supplementary Information section VII.D for more details). A clear limitation here is that cases are a highly selected, non-population-based sample of patients presenting at the ED. However, the upside of this small scale is the detailed investigation of individual cases, similar to canonical studies of sudden death from the literature. This case review identifies 96 arrests caused by cardiac arrhythmia (37% of arrests), on the basis of the totality of evidence collected by research staff (patients’ ED and hospital course, interviews of emergency services and family, medical record review at discharge). Following other case–control studies of sudden cardiac death^29^, we identify the most recent ECG before the ED visit for both arrests and control individuals (median 391 days, interquartile range 74 to 2,189, from ECG to ED visit), then apply our model with no fine-tuning or other modifications. Our model distinguishes future arrhythmic arrests from controls with a zero-shot AUC of 0.767 (95% CI: 0.706–0.823). The dataset’s case–control structure means that population-based event rates are not defined, so we cannot calculate PPV (although we provide some clinically relevant metrics for illustrative purposes in Supplementary Information section II.A,B; see ref. ^30^).

Using detailed information on the cause of arrest in this dataset, we develop a precise test of our model’s specificity for arrhythmic arrests. We observe both arrhythmic and non-arrhythmic arrests (from pulmonary or neurological causes), which can easily be misclassified as arrhythmic^28^. This lets us calculate a ‘placebo’ AUC: how well does the model discriminate these non-arrhythmic arrests versus controls? Comparing this AUC to the arrhythmic arrest AUC above, we should find poor performance if our model is specific to arrhythmia, but if it inappropriately flags both arrhythmic and non-arrhythmic deaths as high risk, it should perform well. Empirically, this placebo non-arrhythmic AUC is significantly worse (*P* < 0.001) than the arrhythmic AUC—indeed, it approaches random guessing: 0.582 (95% CI: 0.529−0.636). This provides reassurance on the model’s specificity for arrhythmic deaths as they are conventionally defined, by detailed case review.

## Defibrillators and mortality in high-risk patients

Without randomization, we cannot know whether defibrillators would lower mortality in high-risk patients. We can, however, provide suggestive evidence by comparing high-risk patients who received defibrillators to patients at similar risk who did not. If those with defibrillators die less than expected, it could suggest a benefit, although unmeasured confounding can bias causal estimates in observational data; we discuss this in more detail below. Returning to the Swedish data, we perform ordinary least squares (OLS) regression of sudden cardiac death (based on death certificates) on age, sex and (i) an indicator for whether an ECG was high risk, (ii) an indicator for whether the patient had a defibrillator and (iii) an interaction term indexing high-risk patients with defibrillators. The coefficient on the latter is the quantity of interest: it captures mortality differences in high-risk patients with defibrillators, relative to what we would expect on the basis of (i) their high risk and (ii) the fact that they had a defibrillator, independently. We use OLS rather than logistic regression, despite a binary outcome, because it is useful to interpret coefficients as changes in absolute probabilities.

We find that high-risk patients with a defibrillator in place are significantly less likely to die than predicted: Table 2, column 1 shows that the interaction term (defibrillator placement × high-risk) is large, negative and significant. In absolute terms, high-risk patients with defibrillators die 3.62 percentage points (p.p.) less than their predicted rate of 6.65 p.p., a reduction of 54.4% (*P* < 0.001; the predicted rate sums the baseline rate in the low-risk population; that is, intercept plus age and sex effects at population means, with the high-risk indicator and the defibrillator indicator.) The defibrillator coefficient probably captures the opposing causal effect of defibrillators (negative) and selection bias (positive)—but the lack of easy interpretation does not detract from its utility as a control variable. Note that if doctors are better at selecting patients for defibrillators on the basis of sudden cardiac death risk, compared with all-cause mortality risk, we might expect more selection bias—and thus more positive coefficients—in columns 1–3 than in columns 4–6, as we observe here.

**Table 2:** Regression analysis of differences in sudden cardiac death and all-cause mortality for patients with and without defibrillators

	(1)	(2)	(3)	(4)	(5)	(6)
	Sudden cardiac death			All-cause mortality		
	ECG model	LVEF	Both	ECG model	LVEF	Both
**Risk indicator variables**						
ECG high risk	0.0639*** (0.002)		0.0602*** (0.002)	0.315*** (0.005)		0.3091*** (0.005)
LVEF high risk		0.0392*** (0.002)	0.0309*** (0.002)		0.0928*** (0.005)	0.0499*** (0.005)
Defibrillator present	−0.0019 (0.001)	−0.0001 (0.001)	−0.0018 (0.001)	−0.0406*** (0.003)	−0.0332*** (0.003)	−0.0463*** (0.003)
**Defibrillator × high-risk interactions**						
**Defibrillator** **×** **ECG high risk**	−0.0362*** **(0.003)**		−0.0332*** **(0.003)**	−0.1264*** **(0.009)**		−0.1309*** **(0.009)**
Defibrillator × LVEF high risk		−0.0299*** (0.003)	−0.0268*** (0.003)		−0.0053 (0.009)	0.0032 (0.009)
Baseline outcome rate	0.0045*** (0.0002)	0.0052*** (0.0002)	0.004*** (0.0002)	0.0497*** (0.0007)	0.055*** (0.0007)	0.0489*** (0.0007)

Columns 1–3 show coefficients from regressions of sudden cardiac death on high-risk indicators (column 1, ECG model high-risk group; column 2, reduced LVEF; column 3, both), an indicator for whether a defibrillator was implanted and an interaction term, shown in bold: this is the coefficient of interest, which captures mortality differences in those with defibrillators relative to expected. Columns 4–6 show regressions of all-cause mortality on the same variables. The interaction effect captures the difference in mortality between high-risk patients with versus without defibrillators. *n* = 119,541 for all regressions, which is the full sample including those with defibrillators, restricted to those with one-year follow-up. All models control for patient age and sex. Standard errors (s.e.) are shown in parentheses and clustered by patient. *P < 0.05, **P < 0.01, ***P < 0.001. Baseline rate combines model intercept with age and sex effects (set to population means; s.e. accounts for covariances) to estimate outcome rate in low-risk patients without defibrillators.

Given the clear risk of unmeasured confounding, we perform a test of face validity by replicating this analysis for patients with reduced LVEF, in whom causal effects are known from several randomized trials: if our regression framework is valid, it should ‘rediscover’ these known effects. Column 2 shows the result: those with reduced LVEF and defibrillators die 2.99 p.p. less than their predicted rate of 4.43 p.p., a 67.5% reduction, in the same neighbourhood as trial effect sizes (50–88% for trials enrolling based on LVEF, see Supplementary Information section II, boldface). Column 3 compares the two predictors directly, using indicators for both high-risk ECG and reduced LVEF, as well as their respective interaction terms. Both interaction terms are negative, but the risk reduction in the ECG model’s high-risk group is larger in absolute terms. We impute LVEF where missing; Supplementary Information section IV.A shows similar results in those with measured LVEF.

We also investigate whether those with defibrillators have lower all-cause mortality, by substituting this as the dependent variable in the regressions. This is important: some trials have found large defibrillator effects on sudden cardiac death, but null results for all-cause mortality, which is likely to be a result of competing causes (that is, the defibrillator might have worked but the patient dies from something else). Column 4 shows that the ECG model’s high-risk group has a 12.6 p.p. (39.0%) reduction in all-cause mortality relative to that predicted (32.4 p.p.) with defibrillators. Column 5 shows that those with LVEF ≤ 35% and defibrillators have a 0.5 p.p. (4.6%) non-significant reduction relative to that predicted (11.5 p.p.). Column 6 shows that only patients with high-risk ECGs have mortality reductions, irrespective of LVEF.

Although these results are unlikely to generalize to the general population of high-risk patients—they are estimated in patients selected for defibrillators under current practice norms—they nonetheless provide encouraging evidence to support future investigation. Furthermore, because defibrillators were associated with reductions in both sudden cardiac and all-cause mortality in those with high-risk ECGs, it increases confidence that our model identifies preventable arrhythmic deaths, irrespective of the death-certificate cause of death.

## Comparison of ECG model and LVEF

Our ECG model, like LVEF, has practical advantages conducive to widespread use: both are biomarkers derived from common medical imaging tests, and both identify high-risk groups for sudden cardiac death who may benefit from defibrillators. Both ECGs and ultrasounds are standardized, ubiquitous, and easy to acquire. Given these similarities, it is important to ask whether the ECG simply rediscovers a known set of high-risk patients, already flagged by reduced LVEF, or whether it identifies new, previously unsuspected high-risk patients. We investigate this in the Swedish lockbox, by measuring the overlap between the 22.5% of our sample with LVEF measured, the 1.9% of the sample known to have reduced LVEF ( ≤ 35%) and the 2.2% of the sample in the model’s high-risk group (results for the US cohort are in Supplementary Information Section IV.B).

**Fig. 3:**
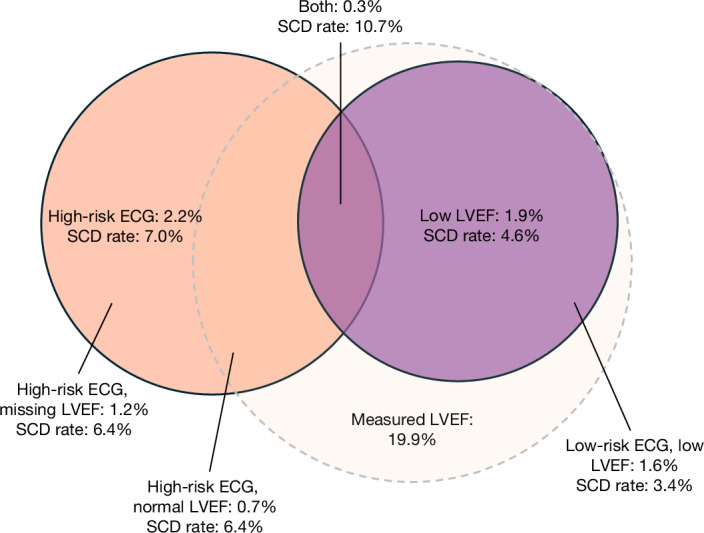
Overlap of the high-risk ECG group and the reduced LVEF group in Swedish data. For each set, we show the fraction of the sample accounted for by the set and the rate of sudden cardiac death (SCD) based on death certificates in that group (*n* = 113,072 ECGs). The measured LVEF circle is not drawn to scale.

Figure 3 shows that only 13.9% of the high-risk ECG group were known to have reduced LVEF, indicating that the ECG identifies large numbers of net new high-risk patients. The ECG model’s high-risk group overall has a higher sudden cardiac death rate than those with reduced LVEF: 7.0% versus 4.6% (*P* = 0.02). If we instead set the size of the ECG high-risk group equal to that of the reduced LVEF group (that is, 1.9% of the sample), sudden cardiac death rates are 7.3% versus 4.6%, respectively (*P* = 0.03). When both biomarkers agree that a patient is high risk, the sudden cardiac death rate is 10.7%, implying that LVEF adds independent risk information. Patients with reduced LVEF who are not in the model high-risk group had a rate of 3.4%, whereas patients in the model high-risk group with normal or unknown LVEF had a rate of 6.4%. Notably, even among those with a measured and normal LVEF, for whom there is currently no means of risk prediction^29^, the ECG model identifies a group with a risk higher than in those with reduced LVEF (6.4% versus 4.6%, respectively). Supplementary Information Section IV.C shows similar results in subgroups defined by risk factors and LVEF for both the Sweden and the US cohorts.

## An ECG biomarker discovered by the model

Many cardiovascular discoveries over the past 100 years started with a correlation: a curious ECG in a patient with a salient outcome—for example, in 1986, the ‘dolphin-like’ waveform noted by Pedro Brugada in the ECG of a three-year-old boy with multiple cardiac arrests^31^. If such correlations hold up in larger samples, researchers start to reason about potential mechanisms, working from first principles: what could cause both the curious waveform and the outcome? This generates testable hypotheses—for example, experiments suggesting that sodium channels drive electrical instability in Brugada syndrome^32^. The earlier discoveries of Herrick and Smith (1918)^33^, Wolff, Parkinson and White (1930)^34^ and others followed a similar pattern.

The human process of noticing correlations is prone to error. Some are spurious; others are missed owing to visual or computational limits^35,36^. Machine learning avoids many of these pitfalls: here, it robustly correlates the appearance of an ECG with sudden cardiac death. But our model cannot easily translate correlation into discovery because there is no curious ECG feature to inspect. Standard interpretability methods (for example, saliency maps) show only where a model ‘looks’, not what it ‘sees’, yielding no substrate for first principles to be applied or new hypotheses to be formed. Nor can a human simply look at ECG instances to reverse-engineer the model. ECGs are complex, high-dimensional signals, so comparing high-risk and low-risk waveforms will reveal many differences, most unrelated to risk^37^. Indeed, if such comparisons were straightforward, we would not need machine learning: humans would have discovered the predictors of sudden cardiac death already, simply by comparing the ECGs of deceased individuals with those of surviving individuals.

We frame the task of visualizing the model’s ‘discovery’ as a dialogue between (i) our predictive model, which can quantify the risk of an arbitrary ECG waveform, and (ii) a new generative model, which can produce arbitrary ECG waveforms. Risk predictions from the former guide the latter, incentivizing it to produce progressively higher-risk counterfactual ECG beats. Details are in Supplementary Information section V.A-B. The result is a series of ECG waveforms that, starting with an actual low-risk patient’s ECG, progressively morph it into counterfactual higher-risk ECGs for that patient. This allows us to isolate the risk signal ‘seen’ by the predictive model in particular ECG instances, holding constant the many other dimensions of variation across patients.

**Fig. 4:**
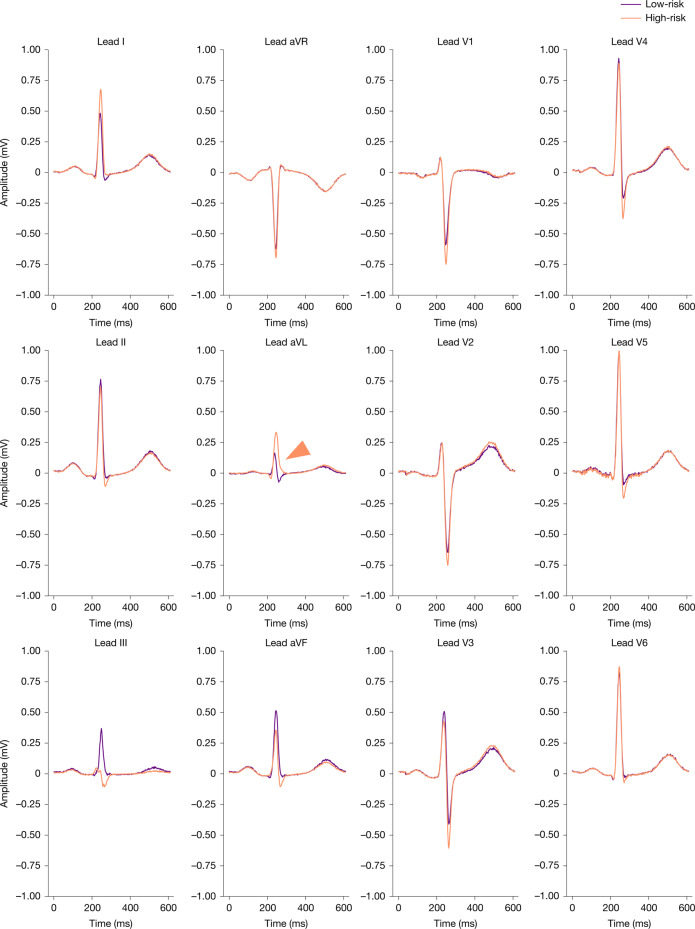
Low-risk versus high-risk ECG waveform morphs. The ECG beat of a representative single patient (low risk; dark purple) and its high-risk morph (light orange), measured on 12 ECG leads. The orange arrowhead indicates a novel feature identified in lead aVL.

Figure 4 compares a low-risk ECG (dark purple) with its high-risk morph (light orange). The high-risk morph exhibits axis deviation, or rotation in the average electrical vector of depolarization. In the frontal plane, we see left axis deviation (higher amplitudes in leads I and aVL; lower amplitude in lead III), consistent with left anterior–superior fascicle blockage (LAFB). In the axial plane, we see posterior rotation or ‘poor R-wave progression’. Supplementary Information section V.C contains a brief primer on the physiology underlying the ECG; for more detail on these findings, see Supplementary Information section V.D. All these morphologies are well-known correlates of ischaemic heart disease, making it interesting—but not surprising—that the model links them to sudden cardiac death. Supplementary Information section V.E shows that the changes seen in this individual ECG are quite general, and present in median beats for low-risk versus high-risk morphs, and low-risk versus high-risk actual beats. However, they are less visible in a simple comparison of sudden cardiac deaths and others, highlighting the importance of the predictive model.

Figure 4 also shows an intriguing morphology in the high-risk QRS complex of lead aVL, which, unlike axis deviation, has not been previously described to our knowledge. The terminal aspect of the R wave is slurred (arrow), replacing the sharp negative S wave seen in the low-risk morph. A saliency map for this ECG (Supplementary Information section V.F) agrees that this region contributes prominently to predictions, but yields no further clues.

**Fig. 5:**
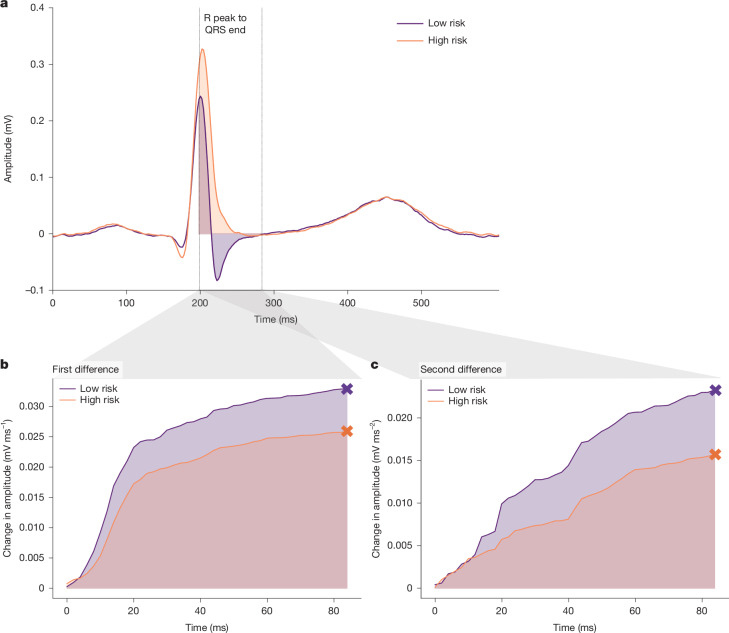
A biomarker identified in the ECGs of high-risk patients. **a**, aVL detail (from Fig. 4). The box shows the interval from the peak of the R wave to the end of the QRS complex, over which quantitative features are calculated (intervals differ slightly for low-risk and high-risk morphs, but for clarity only one interval is shown). **b**, Running absolute first difference for low-risk (purple) and high-risk (orange) morphs. Starting from R peak, we calculate the absolute change in aVL amplitude, and plot the running, cumulative total until QRS end; all sums are divided by the length of the total interval so that the final points, denoted by crosses, show the actual feature values (that is, the mean absolute difference over the interval). **c**, As in **b**, for the second difference of aVL amplitude.

We confirm that this morphology in aVL is, alone, a robust predictor of sudden cardiac death by quantifying one aspect of its shape, using the first and second differences of QRS amplitude as a proxy. Figure 5 shows how we implement this, and the differences between high-risk and low-risk morphs. From R peak to QRS end (identified using standard methods^38^) we sum the absolute first and second voltage differences, and take the mean. Regression of sudden cardiac death on these features, both with and without controls used in quantitative ECG analysis (rate, intervals, axes and so on), shows that coefficients are large, negative and significant in both Sweden and the USA; in Taiwan, they are similarly large and negative, but less precisely estimated and not significant, probably owing to the smaller sample (Supplementary Information section V.G). We can directly compare the magnitude of coefficients from these new features to the known feature of left axis deviation, a measure of LAFB: the new features add independent, significant predictive signal, of approximately the same magnitude (in s.d. units) as left axis deviation (Supplementary Information section V.G). In Supplementary Information section V.H, we train a new set of models, each using data from a single ECG lead to predict sudden cardiac death, to determine whether predictive signal is concentrated in a particular view of the heart. We find that these single-lead models have AUCs almost as high as that of the 12-lead model (albeit lower PPVs), suggesting that the model is capturing a diffuse process throughout the myocardium.

The new features produced by the generative model relate to several known features of ECGs. For example, intrinsicoid deflection^39^ has been linked with cardiac adverse events, but affects the early parts of the QRS complex, not the later parts we focus on here. Empirically, intrinsicoid deflection was not increased in our model’s high-risk waveforms. Fragmented QRS morphology has also been linked to risk, but its RSR′ or notching pattern is likely to create increased rather than decreased first and second derivatives. Late potentials are also related, but these small-amplitude signals require special processing to observe, and are not visible on standard ECGs. Finally, QRS duration has been linked to sudden cardiac death; however, QRS interval is distinct from first or second differences over the interval, and in any case, we control for QRS duration in all regressions alongside the new features.

In summary, the model identifies an ECG feature that is easily visible and robustly predicts sudden cardiac death, but has not to our knowledge been previously described in the literature.

## Clinical and scientific implications

Our results suggest two broad directions for future work, both practical and scientific. Practically, high-risk patients seem to represent a new, previously unsuspected population with frequent, preventable death. This risk can be confirmed with outpatient cardiac monitoring. Recent advances in defibrillator technology—for example, subcutaneous devices or patches^40,41^—make the placement of defibrillators in novel high-risk groups more attractive. A randomized trial in high-risk patients will be crucial: many promising predictors in the past have failed to identify patients who benefit from defibrillators^42^.

Scientifically, our methods open up new ways to study the mechanisms that underlie sudden cardiac death. First, computational models of the heart provide increasingly sophisticated ways to simulate how electrophysiological processes, captured by model parameters, generate surface ECG waveforms^16,43^. By linking our generative model to these computational models, new high-risk ECG morphologies can be tied back to specific mechanisms, suggesting novel targets for diagnostics or treatments for sudden cardiac death. Second, better prospective predictions enable new kinds of data collection for high-risk patients. Today, attention focuses on small groups of patients—for example, individuals with genetic abnormalities, prior arrhythmias or reduced LVEF—who make up a small minority of actual deaths; the vast majority of deaths are studied only ex post. By flagging new high-risk patients ex ante, we can study them in detail, considering ‘red-flag’ symptoms that might herald arrhythmias^44^, cardiovascular and genetic risk factors^5,45^, cardiac structure (for example, cardiac ultrasound and cMRI) and, especially, cardiac function (for example, electrophysiological studies and provocative testing)^19^. The latter is both novel and crucial: although the heart’s structure can be studied post mortem, its function cannot, leaving a major gap in our understanding of the electrical conditions that precipitate sudden cardiac death.

To illustrate this scientific potential, we generate and preliminarily test one mechanistic hypothesis on sudden cardiac death. The slurred downstroke at the end of the QRS complex in high-risk patients could indicate that the electrical vector of depolarization becomes progressively more orthogonal, or more disorganized, with respect to the lead over time. This could suggest an accretive process that worsens as depolarization progresses. Although linking our results to an established computational model is beyond the scope of this work, we can gain some intuition from a thought experiment (see Supplementary Information section VI.A) involving a highly stylized, uniform sheet of myocardial cells. Distributing obstacles to conduction at random in such a sheet might produce something like the biomarker we observe. Intuitively, obstacles will cause the electrical wavefront of depolarization to split repeatedly, analogous to scatter in other settings. A lead oriented parallel to the initial wave would thus record an average vector that grows increasingly orthogonal over time, as scattering progresses. We might see such changes most clearly in lead aVL because its axis matches the (leftward-rotated) axis of high-risk patients, or because the positive electrode for lead aVL is positioned closest to the base of the heart—the most distal part of the heart’s conduction system—and is thus best situated to capture the end of depolarization.

For clues about which physical phenomena could produce such diffusely distributed obstacles to conduction, we turned to cMRIs, present in the health records of a small number of patients in our validation set. Blinded review showed that patients in the riskiest 10% of predictions had a significantly higher prevalence of subtle, diffuse late gadolinium enhancement (LGE) throughout the left ventricle (see Supplementary Information section VI.B for further details on MRIs). This pattern, representing differences in extracellular contrast uptake, is most often associated with myocardial fibrosis: the deposition of electrically inert collagen tissue between myocardial cells. This effectively insulates cells, and is known to alter conduction^46^.

Fibrosis—a diffusely distributed phenomenon that alters conduction—is one mechanism that ties together the ECG biomarker and sudden cardiac death. It could also explain the left axis deviation in high-risk patients. Although fibrosis is a diffuse problem, it often manifests first as left anterior fascicular block (LAFB), causing left axis deviation: this fascicle is the thinnest and most vulnerable to disruption, for example, by randomly distributed lesions^47^. LAFB has itself been linked to risk of cardiac events and death^48^.

We are not the first to hypothesize that fibrosis could cause sudden cardiac death^49^. However, studying fibrosis is difficult: endomyocardial biopsy is the only way to establish the diagnosis, and studies comparing cMRI with biopsy show that LGE is often falsely negative in diffuse, subtle fibrosis of the kind suggested by the ECG biomarker^50^. As a result, both researchers and clinicians might underestimate its importance. As one metric of this disregard, we reviewed cardiologists’ notes after they received the results of cMRIs in high-risk patients. We found no mention of LGE, fibrosis or its implications in any patient record. Although these findings are speculative, future work to correlate ECGs with biopsy could be valuable for identifying a broad set of pathophysiological factors.

In conclusion, deep learning identifies new patients who are at high risk of sudden cardiac death, in data from three continents. These high-risk patients are overlooked by doctors, and could benefit from defibrillators, an observation with great practical importance. In addition, the model suggests new avenues for detailed study of this tragic and widespread medical mystery.

## Methods

### Study cohort and outcomes: Sweden

We obtained all 441,614 ECGs done from 2010 to 2016 in Region Halland, a public regional health system in Sweden. (Twelve patients in the region opted out of participation in research, so we did not include their ECGs.) We linked these to death certificates and patient electronic health records, which capture all interactions between patients and the national health-care system that oversees all care in Sweden^51^. ECGs were sampled at 500 Hz and retrieved in XML format from a Philips IntelliSpace system. This research was approved by the ethical review board of Lund University (protocol 2016/517 and amendment 2024-02316-02).

Before performing any analysis, we created strict random splits in our dataset to safeguard against overfitting (see Supplementary Information section VII.A for a CONSORT-style diagram). We first created a data lockbox by randomly sampling 40% of patients and all of their ECGs. The lockbox remained untouched from model development through peer review, until provisional acceptance of the manuscript. The remaining 60% was split in half at the patient level, one half for training and the other half for validation, and used for initial submission and the usual peer-review process. On provisional acceptance of the manuscript, we retrained the model on the 60% of data we had accessed, 262,554 ECGs from 75,157 patients, and applied the resulting model with no modification to generate predictions in the 40% lockbox: 179,060 ECGs from 51,481 patients. Those results are shown here. Supplementary Information section VII.A records changes between the initial submitted version and the present version. Model performance improved, consistent with a larger training set size (for example, AUC went from 0.837 to 0.872), providing additional reassurance with regard to overfitting.

We perform all analyses at the ECG level, to account for risk variation over time within a patient, and account for within-patient correlation by clustering standard errors by patient. We view this as preferable to selecting one ECG per patient, which reduces sample size and can introduce bias (for example, the most recent ECG selects on those who survived past initial ECGs). All statistical tests are two-sided.

Our primary outcome, sudden cardiac death in the year after ECGs, was censored for ECGs in the final year of our dataset (2016). Although we do not have death certificates after 2016, we do have access to full electronic health records from 2017 onwards, which indicate whether a clinical encounter occurred. If we observe such an encounter in the year after the ECG, we label the outcome as absent, not missing. The result is that only 12,969 out of 247,286 ECG records in the training set and 6,446 out of 125,987 ECGs in the lockbox are censored, and are thus excluded from outcome evaluation metrics (they are used selectively in training, as detailed in Supplementary Information section IX).

Our primary definition of sudden cardiac death is based on death certificates, using standard epidemiological criteria^24^: deaths (i) from cardiac or ill-defined causes, and (ii) occurring outside the hospital or in the first 24 h of hospital stays. Details are in Supplementary Information section VII.B. There are many approaches for measuring sudden cardiac death, each with trade-offs. An idealized definition is ‘arrhythmic death’: death preceded by an arrhythmia that can be terminated by defibrillation (ventricular fibrillation or ventricular tachycardia: VF/VT). Of course, measuring this would require continuous premortem ECG monitoring, which is rare. Most studies thus rely on other data: diagnosis codes from death certificates, medical chart review or autopsy. Detailed chart review and autopsy might provide more certainty about arrhythmic causes of death, but exist only for small samples; death-certificate data achieve larger scale, at the expense of detail.

A large body of research has investigated how well our primary definition agrees with more detailed investigations of arrhythmic deaths (for example, in-depth case review, autopsy). Some studies find close agreement^52^, whereas others find that death certificates are more sensitive than specific for arrhythmic deaths^24–28^. Low specificity would mean that our definition—and thus predictions—might capture a mix of arrhythmic and non-arrhythmic deaths. Assessing model performance on the basis of death certificates only could thus be misleading: the model would seem to perform well, but some fraction of deaths in the high-risk group would be non-arrhythmic deaths, and thus not preventable with defibrillators.

The limitations of any one definition of arrhythmic death make it crucial to use multiple sources of data to validate model predictions. The experiments described above use three such data sources. First, diagnosed ventricular arrhythmias, the mechanism for sudden cardiac death, as documented in health records from both Sweden and an independent US cohort. Second, detailed investigation into the cause of individual cardiac arrests, in our hospital-based registry from Taiwan. Third, direct estimation of potential mortality reductions from defibrillators, comparing patients with and without defibrillators, as a measure of preventability of deaths in high-risk patients. All of this means that we do not rely on death certificates alone to validate predictions, but also incorporate a range of other information across several independent datasets, to isolate preventable arrhythmic deaths.

Our analysis focuses on younger, healthier high-risk patients who could be good candidates for defibrillators, because our ultimate goal is the prevention of arrhythmic deaths. There is no formal age restriction for defibrillator placement^8^, but benefit probably decreases with age. Older patients have more complications from the surgical implantation procedure and are more likely to die of competing causes. Physicians think that benefit diminishes for those over 80 years old^53^, and empirically, only 10% of US defibrillators are implanted in this age group^54^. We thus focus on ECGs done in patients under 80 years old in the main results—74.6% of all ECGs, and 25.7% of all sudden cardiac deaths. Supplementary Information section VIII replicates all main analyses in the entire cohort, and finds that performance is comparable overall when those over 80 are included.

Summary statistics for the lockbox sample are in Table 1. The median follow-up period was 2,010 days. The overall rate of sudden cardiac death in the year after ECGs was 0.6%; 43.9% of these deaths had LVEF measured premortem, and 36.4% of measured LVEFs were low (LVEF ≤ 35%). Nearly half (41.2%) of sudden cardiac deaths had no obvious risk factors at the time of their ECG—no coronary artery disease or myocardial infarction, heart failure or prior ventricular arrhythmias; 10.0% had a recent myocardial infarction (within 40 days before the ECG, versus 3.3% base rate); and 7.7% had defibrillators implanted (versus 5.4% base rate) but nonetheless experienced sudden cardiac death.

### Study cohort and outcomes: USA and Taiwan

We rely on two external datasets to validate model predictions in diverse populations outside of its training context in Sweden. The first is a US cohort of ECGs provided by Dandelion Health, a company that aggregates deidentified medical imaging and health outcomes data from a consortium of health systems across the USA. We obtain all 251,858 ECGs performed in 2021 and 2022, from 139,613 patients under the age of 80 years, sampled at either 500 Hz (83.3%) or 250 Hz (16.7%) and drawn from a GE MUSE storage system. Of note, this is a different manufacturer and format to that of the Philips system from which the Swedish training data were obtained. All 250-Hz ECGs were linearly interpolated to 500 Hz. Supplementary Information section VI.C contains additional details on the population.

The second external dataset is a Taiwanese hospital registry available through the Nightingale Open Science platform^55^, and described in more detail elsewhere^56^. Patients were enrolled after being brought to the hospital ED in cardiac arrest (that is, without measurable cardiac output), or after experiencing arrest in the ED, from 2011 to 2019. Data were entered using an Utstein-style reporting template, and linked to all patient ECGs in hospital records, including those before the arrest. The dataset also contains ECGs for a random sample of control patients who visited the same ED (without arrest). We identify all ECGs before the ED visit, and exclude those done in the two days before visits, which might have been done in the context of the same acute event that precipitated arrest, making them less useful for prevention. The final sample includes 4,268 patients, 257 arrests and 4,011 controls. ECGs were sampled at 500 Hz and retrieved from a GE MUSE ECG storage system. Supplementary Information section VI.D contains further details on the population.

### Predictive model training

Before model development, we surveyed the literature and online competition platforms (for example, PhysioNet) to review deep-learning architectures used for ECG waveforms. After experimenting with several models (convolutional and residual neural networks, transformers and long short-term memory (LSTM) models) and hyperparameter choices (number of layers, dropout probability, learning rate and so on), we settled on a 64-layer ResNet model consisting of 32 residual blocks, with each block made up of 2 convolutional layers with 128 filters and a kernel of size 16. We verified that this model was able to achieve performance matching or exceeding published benchmarks, both for human-visible ECG features (for example, QRS and corrected QT (QTc) intervals, atrial fibrillation and flutter, and so on^57^) as well as less obvious patient characteristics (for example, age, sex^58^ and cardiovascular outcomes^23,59,60^).

The model’s primary training objective was to predict the probability of sudden cardiac death, on the basis of death certificates, in the year after the ECG. To do so, we developed a multitask learning set-up with three main components, each developed on a different subset of training data: (i) in the entire cohort, we predicted sudden cardiac death (over several time frames, as well as other outcomes; see Supplementary Information section IX); (ii) in patients who died within one year of the ECG, we predicted sudden cardiac death versus other causes of mortality; and (iii) in patients with measured LVEF, we predicted reduced LVEF (≤35%). We then used logit to calibrate predictions (formed in patients of all ages) using only patients aged under 80 years old, all in the Swedish training set, to generate final predictions on one-year sudden cardiac death. Further details are provided in Supplementary Information section IX. In both the Swedish lockbox and external validation samples, model predictions are not modified or fine-tuned in any way, to measure ‘zero-shot’ performance in a new dataset (and, in the USA and Taiwan, on a new label).

### Generative model and morphing procedure

Both the generative model and the predictive model that guides it are trained on a dataset of individual beats, viewed across all 12 leads, that are segmented from the full sample of 10-s ECGs using standard methods^38^. Visualizing individual beats rather than 10-s ECGs makes it easier to understand the focused changes in beat morphology identified by the predictive model. To retrain the predictive model, we use the same network architecture and training procedure as those used for the model trained on 10-s ECGs. To train the generative model, we implement a variational auto-encoder (VAE) with 512-dimension latent space that encodes these individual beats. Of note, all results from the generative model and morphing procedure are drawn from the initial dataset (30% training, 30% validation) rather than the lockbox: the process was computationally intensive, and subsequent results involved several rounds of human review (waveforms, blinded interpretation of linked MRIs) that were difficult to perform again in the lockbox. We see this as acceptable because the emphasis of these results is hypothesis generation, rather than predictive performance.

The morphing procedure begins by randomly sampling 56 patient beats for the VAE to encode (the number was chosen given computational constraints, because the full pipeline here took hours to execute for each beat). These beats serve as our point of entry for exploration of the model’s latent space: we identify the gradient of predicted risk around each beat, and perturb its latent vector to follow the gradient. This produces a higher-risk vector, which we then pass through the decoder to reconstruct, resulting in a counterfactual, higher-risk ECG waveform. The new vector is the starting point for another round of perturbation and reconstruction, which we repeat 2,000 times, or until the risk of the generated synthetic beat reaches the 90th risk percentile. Further details on this procedure can be found in Supplementary Information section VII.B, and the accompanying codebase is available at https://github.com/alexmschubert/ECG-SCD.

### Reporting summary

Further information on research design is available in the Nature Portfolio Reporting Summary linked to this article.

## Online content

Any methods, additional references, Nature Portfolio reporting summaries, source data, extended data, supplementary information, acknowledgements, peer review information; details of author contributions and competing interests; and statements of data and code availability are available at 10.1038/s41586-026-10674-6.

## Supplementary information

Supplementary Information This Supplementary Information file contains the following sections: I Additional analyses—Sweden; II Landmark defibrillator trials: Enrolment criteria and sudden cardiac death rates; III Additional performance metrics—Sweden and external validation datasets; IV Additional comparisons of ECG model versus LVEF; V Generative model and electrophysiological mechanisms; VI Hypothesis generation; VII Dataset and key variable construction; VIII Replication of main results—Sweden, all ages; IX Deep-learning model.

Reporting Summary

Peer Review File

## Data Availability

Because our analysis relies on high-dimensional patient data that lack universally accepted standards for deidentification, the availability of data varies by the data source and relevant legal frameworks. For Sweden, access is through a direct collaboration agreement with Region Halland. Inquiries should be directed to M.L.: markus.lingman@vgregion.se. Data from Dandelion Health may be accessed through a research partnership between the company and non-profit entities, or under the company’s standard data access pathway for for-profit entities. Inquiries should be directed to N. Gossen: nick@dandelionhealth.ai. Data from Taiwan are freely available from Nightingale Open Science: https://ngsci.org.

## Code availability

Because our analysis relies on high-dimensional patient data that lack universally accepted standards for deidentification, code availability varies by analysis. For deep-learning models derived from Swedish data that could, in theory, inadvertently encode patient information, the only permissible access pathway under the General Data Protection Regulation of the European Union is a direct collaboration agreement with Region Halland. Inquiries should be directed to M.L.: markus.lingman@vgregion.se. All other code, including implementation of the morphing process, is available at https://github.com/alexmschubert/ECG-SCD.
