# Supplementary material for: An ECG biomarker for sudden cardiac death discovered with deep learning
Source: Nature. 2026 Jun 24;655(8121):210–8. doi: 10.1038/s41586-026-10674-6 (PMC13323061; doi:10.1038/s41586-026-10674-6)
Supplement: Supplementary file 1 — This Supplementary Information file contains the following sections: I Additional analyses—Sweden; II Landmark defibrillator trials: Enrolment criteria and sudden cardiac death rates; III Additional performance metrics—Sweden and external validation datasets; IV Additional comparisons of ECG model versus LVEF; V Generative model and electrophysiological mechanisms; VI Hypothesis generation; VII Dataset and key variable construction; VIII Replication of main results—Sweden, all ages; IX Deep-learning model. [file 41586_2026_10674_MOESM1_ESM.docx]

Supplement to

An ECG biomarker for sudden cardiac death discovered via deep learning

Ziad Obermeyer*, Alexander Schubert, James Ross, Sendhil Mullainathan^†^, Markus Lingman^†^

^*Correspondence to: zobermeyer@berkeley.edu^

^†Equal contribution^

**Contents**

[I Additional analyses—Sweden 2](#_Toc226448862)

[II Landmark defibrillator trials: Enrollment criteria and sudden cardiac death rates 7](#_Toc226448863)

[III Additional performance metrics—Sweden and external validation datasets 8](#_Toc226448864)

[IV Additional comparisons of ECG model vs. LVEF 16](#_Toc226448865)

[V Generative model and electrophysiological mechanisms 19](#_Toc226448866)

[VI Hypothesis generation 32](#_Toc226448867)

[VII Dataset and key variable construction 36](#_Toc226448868)

[VIII Replication of main results—Sweden, all ages 46](#_Toc226448869)

[IX Deep learning model 50](#_Toc226448870)

#### I Additional analyses—Sweden

##### I.A Additional performance metrics, clinically relevant subgroups—Sweden, under 80 years old

This section supplements the performance measures presented in the main text.

*Figure I.A.1* shows model PPV and calibration for subgroups. These confirm the results in the main text (Figure 3), showing that LVEF (as well as prior MI) add independent predictive power to the ECG-based risk predictions (top two lines), with both groups having higher rates conditional on predicted risk. We next show additional performance metrics commonly used in the literature.

***Figure I.A.1: Positive predictive value for sudden cardiac death, by clinical subgroups.*** *Rate of sudden cardiac death (from death certificates) in high-risk groups (y-axis, 95% CIs suppressed for visibility), vs. percentile threshold used to define high-risk group (x-axis); inset zooms in on the top 10%.*

***Figure I.A.2: Calibration by clinical subgroups.*** *For each* *group, we show 10-bin (i.e., deciles with equal numbers of observations) calibration.*

|  |  | All | LVEF ≤35% | LVEF >35% | No EF | Recent MI (40d) |
| --- | --- | --- | --- | --- | --- | --- |
|  | n | 113,072 | 2,104 | 23,292 | 87,676 | 3,681 |
|  | Base rate | 0.0058 | 0.0461 | 0.0079 | 0.0043 | 0.019 |
| *Threshold-independent classification metrics* | | | | | | |
|  | AUC | 0.872 | 0.742 | 0.841 | 0.871 | 0.878 |
|  |  | (0.843, 0.899) | (0.623, 0.823) | (0.765, 0.891) | (0.838, 0.901) | (0.751, 0.937) |
|  | AUPRC | 0.049 | 0.099 | 0.047 | 0.043 | 0.113 |
|  |  | (0.033, 0.071) | (0.050, 0.200) | (0.022, 0.093) | (0.025, 0.075) | (0.051, 0.211) |
| *Calibration metrics* | | | | | | |
|  | Brier Score | 0.006 | 0.044 | 0.008 | 0.004 | 0.018 |
|  |  | (0.005, 0.007) | (0.026, 0.064) | (0.005, 0.011) | (0.003, 0.005) | (0.010, 0.026) |
|  | ECE^†^ | 0.001  (0.001, 0.002) | 0.023  (0.013, 0.046) | 0.001  (0.001, 0.004) | 0.001  (0.000, 0.002) | 0.008  (0.003, 0.019) |
|  |  |  |  |  |  |  |
|  | Log- loss | 0.030 | 0.188 | 0.040 | 0.023 | 0.076 |
|  |  | (0.025, 0.035) | (0.123, 0.256) | (0.030, 0.051) | (0.019, 0.029) | (0.047, 0.104) |
| *Threshold-dependent classification metrics* | | | | | | |
|  | High risk | 0.022 | 0.1649 | 0.0357 | 0.0149 | 0.0671 |
|  | PPV | 0.07 | 0.107 | 0.064 | 0.064 | 0.134 |
|  |  | (0.050, 0.094) | (0.041, 0.196) | (0.030, 0.110) | (0.038, 0.096) | (0.053, 0.233) |
|  | Sensitivity | 0.265 | 0.381 | 0.29 | 0.223 | 0.471 |
|  |  | (0.197, 0.333) | (0.185, 0.561) | (0.167, 0.396) | (0.141, 0.313) | (0.256, 0.636) |
|  | F1 Score | 0.111 | 0.167 | 0.104 | 0.1 | 0.208 |
|  |  | (0.081, 0.146) | (0.068, 0.284) | (0.050, 0.169) | (0.059, 0.145) | (0.086, 0.334) |
|  | Accuracy | 0.975 | 0.824 | 0.961 | 0.983 | 0.932 |
|  |  | (0.973, 0.978) | (0.781, 0.861) | (0.954, 0.967) | (0.981, 0.984) | (0.913, 0.948) |
|  | Balanced Accuracy | 0.622  (0.588, 0.656) | 0.613  (0.515, 0.703) | 0.628  (0.566, 0.681) | 0.605  (0.564, 0.649) | 0.706  (0.598, 0.790) |
|  |  |  |  |  |  |  |
| *^†^ Expected Calibration Error, computed with 10 equally-sized (decile) bins.* ***Table I.A.1: Performance metrics*** *(columns) for different sub-populations (rows). 95% confidence intervals are constructed via 1000 bootstrap samples at patient level.* | | | | | | |

##### I.B Comparison of ECG-based risk model discrimination vs. other predictive models across clinically relevant subgroups —Sweden, under 80 years old

***Table I.B.1*** compares the ECG model AUC for sudden cardiac death to a variety of other risk predictors: LVEF, AHA/ACC 10-year ASCVD score, and a previously-validated ECG model that predicts longer-term cardiovascular risk called SEER.

|  | ***n*** | | **AUC (95% CI)** | | | |  |
| --- | --- | --- | --- | --- | --- | --- | --- |
|  |  |  |  |  |  |  |  |
| *Population* | *Patients* | *ECGs* | *ECG-SCD* | *LVEF* | *AHA/ACC ASCVD10* | *SEER ECG model* |  |
| Full population | 35,417 | 113,072 | **0.872** | 0.724 ^†^ | 0.697 | 0.655 |  |
|  |  |  | (0.843, 0.899) | (0.661, 0.783) | (0.646, 0.750) | (0.617, 0.694) |  |
| Recorded EF | 5,707 | 25,396 | **0.850** | 0.724 ^‡^ | 0.628 | 0.666 |  |
|  |  |  | (0.797, 0.890) | (0.639, 0.803) | (0.538, 0.737) | (0.598, 0.726) |  |
| EF≤35% | 351 | 2,104 | **0.742** | 0.584 ^‡^ | 0.611 | 0.608 |  |
|  |  |  | (0.623, 0.823) | (0.367, 0.810) | (0.442, 0.789) | (0.527, 0.671) |  |
| EF>35 | 5,555 | 23,292 | **0.841** | 0.703 ^‡^ | 0.620 | 0.649 |  |
|  |  |  | (0.765, 0.891) | (0.589, 0.790) | (0.497, 0.744) | (0.545, 0.743) |  |
| No EF recorded | 34,311 | 87,676 | **0.871** | 0.697 ^†^ | 0.713 | 0.64 |  |
|  |  |  | (0.838, 0.901) | (0.622, 0.766) | (0.663, 0.768) | (0.600, 0.680) |  |
| EF>35 or missing | 35,378 | 110,068 | **0.865** | 0.707 ^†^ | 0.694 | 0.644 |  |
|  |  |  | (0.831, 0.895) | (0.633, 0.767) | (0.636, 0.750) | (0.599, 0.686) |  |
| MI in last 40 days | 937 | 3,681 | **0.878** | 0.722 ^†^ | 0.692 | 0.595 |  |
|  |  |  | (0.751, 0.937) | (0.538, 0.898) | (0.532, 0.847) | (0.489, 0.672) |  |
| ^†^ Uses a model with age, sex, LVEF (with LVEF imputed in those without measured LVEF), and an indicator for missing LVEF | | | | | | |  |
| ^‡^ Uses a model with age, sex, and LVEF | | | | | | |  |

##### ***Table I.B.1: AUC of different predictors, by clinical subgroup.*** *Predictors are in columns, different sub-populations are in rows. 95% confidence intervals are constructed via 1000 bootstrap samples at patient level. Highest AUC in each subpopulation is shown in boldface.*

##### I.C Survival analysis—Sweden, under 80 years old

Figure I.C.1 shows time-to-event analysis to characterize when sudden cardiac death (SCD) and death from any cause occur after the index ECG. Kaplan–Meier curves (Panel A) show early and sustained separation between strata: the estimated 2-year cumulative incidence of SCD was 11.0% in high-risk patients versus 0.7% in low-risk patients. Panel C shows similar patterns among patients with LVEF recorded: the 2-year KM-estimated cumulative incidence was 6.9% for LVEF≤35% and 1.3% for LVEF>35%, while 0.7% of patients with missing LVEF experienced SCD by 2 years. Notably, patients without recorded LVEF (78.2% of the sample) had low observed event rates overall, despite some being assigned high predicted risk by the ECG model.

*A) SCD: ECG High- vs. Low-risk B) All-cause: ECG High- vs. Low-risk*

*
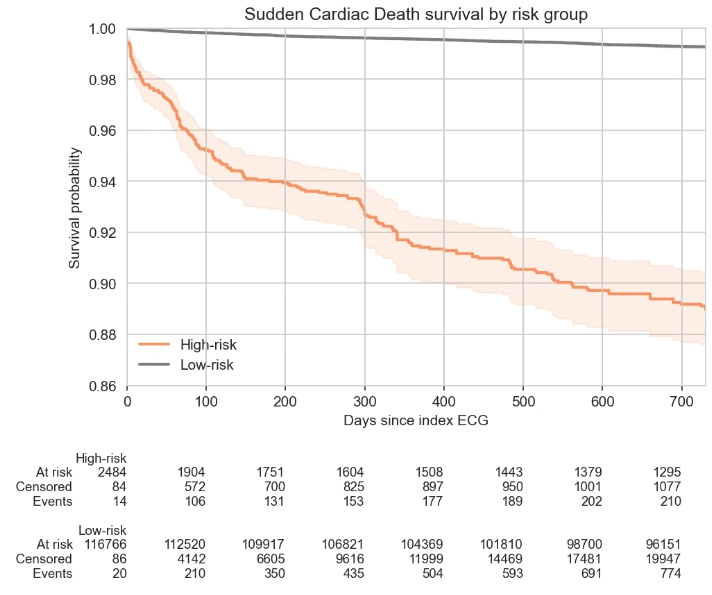

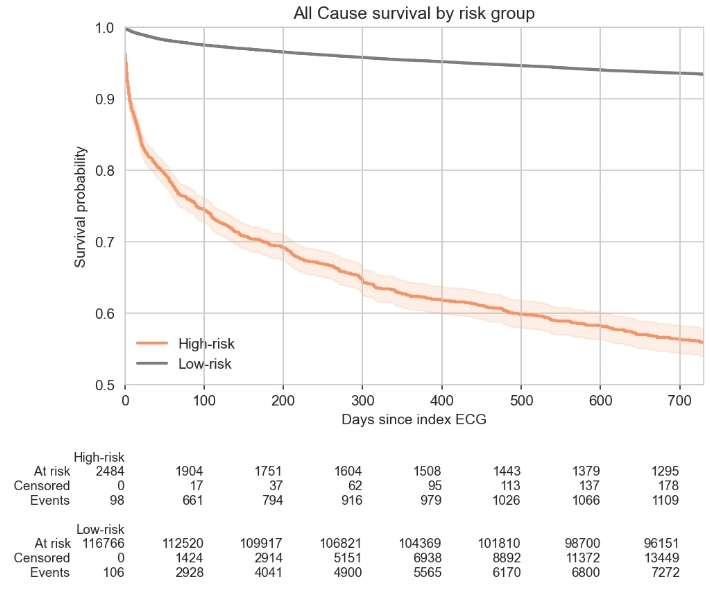
*

*C) SCD: By LVEF status D) All-cause: By LVEF status*

*
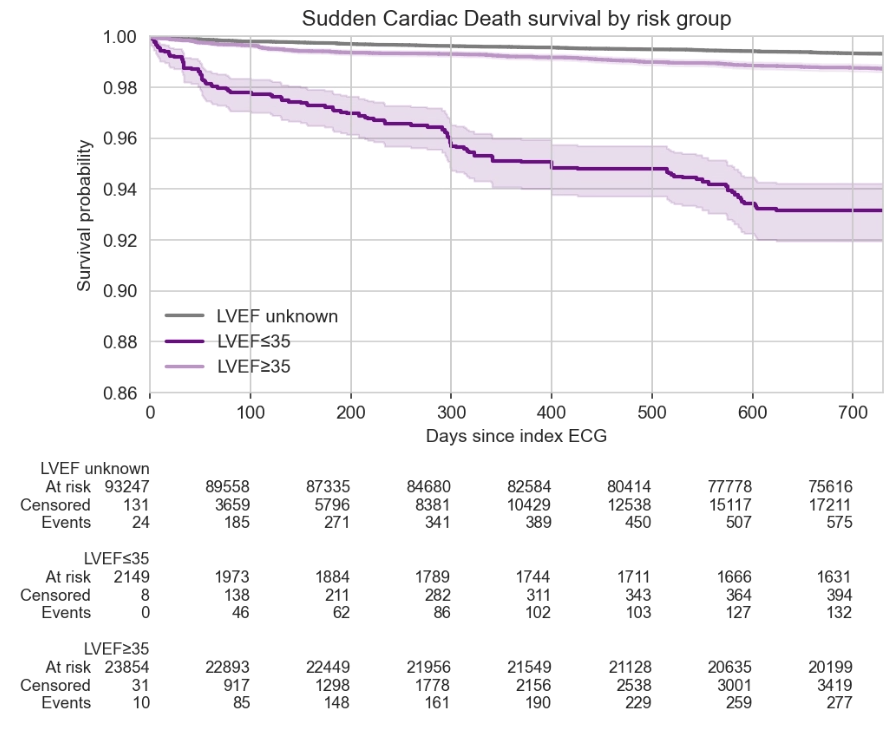

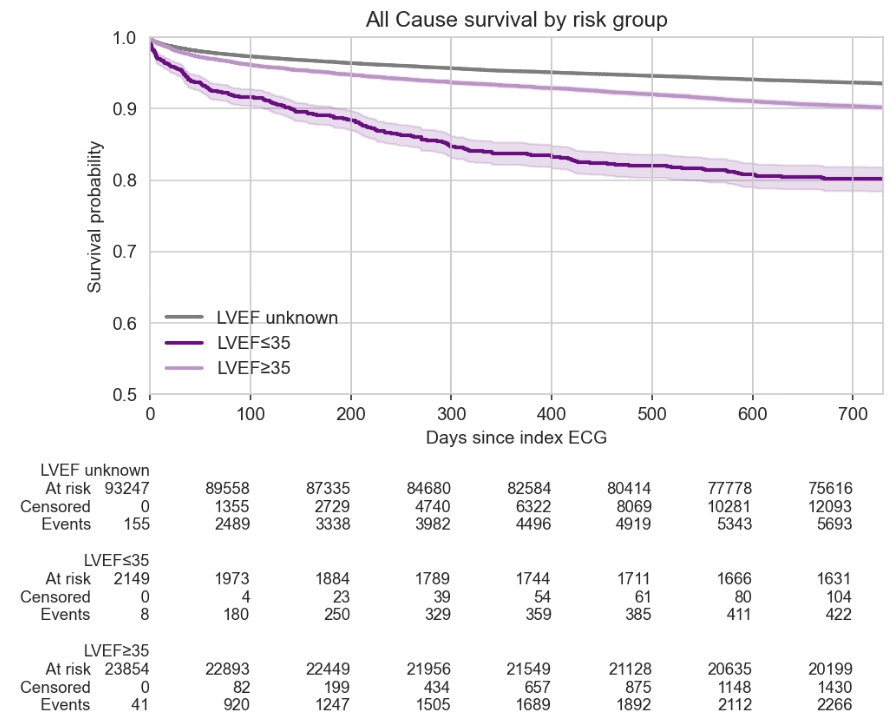
*

*E) SCD: ECG High-risk * Low LVEF F) All-cause: ECG High-risk * Low LVEF*

**

***Figure I.C.1. Survival analysis for high- vs. low-risk patients, for ECG-based model vs. LVEF.*** *The top two panels show sudden cardiac death rates and all-causes mortality over the follow-up period after ECG, separated by ECG model high-risk (top 1.8 [2.2]%) vs. low-risk (all others). The middle two panels show the same for those with low- vs. normal vs. unmeasured LVEF. The bottom two panels do the same, dividing the sample into groups based on the combination of ECG model predicted risk and LVEF. Note these analyses include ECGs considered censored in the main text (125,987 ECGs from 38,695 patients), assigned to the appropriate time interval.*

Comparing the top vs. middle panels, we observe that the model identifies a group that is at least as high risk as those with reduced LVEF over the two years after the ECG. The bottom panels show that by far the worst survival is observed among those with both reduced LVEF and high model-predicted risk based on the ECG.

Interestingly, the model is quite good at predicting risk among unknown-LVEF patients, likely because there are many instances to learn from (78.2% of the sample has unknown LVEF). So while these patients have low outcome rates on average—because doctors tend to measure LVEF in patients they suspect are high-risk—the model is able to identify high-risk subsets.

#### II Landmark defibrillator trials: Enrollment criteria and sudden cardiac death rates

We synthesize major trials, focusing on the enrollment criteria, the rate of sudden cardiac death in the control group, and the reduction in risk from defibrillators. Trials enrolling patients based on reduced LVEF are shown in boldface.

| **Trial Name** | **Year** | **Sample**  **size** | **Enrollment**  **criteria** | **Treated SCD rate** | **Control SCD rate** | **Reduction (%)** |
| --- | --- | --- | --- | --- | --- | --- |
| **MUSTT** | 1999 | 670 (Treatment: 161, Control: 509) | CAD, **LVEF ≤ 40**, VT in last 6 months | 1.30% | 11.00% | 88.2 |
| CIDS | 2000 | 659 (Treatment: 328; Control: 331) | At least one of: VF, VT, cardiac arrest, manifested in the absence of  acute MI (≤ 72 hours) or electrolyte imbalance | 4.37% | 6.23% | 29.9 |
| CASH | 2000 | 288 (Treatment: 99; Control: 189) | Patients resuscitated from cardiac arrest from documented  sustained ventricular arrhythmias | 1.30% | 9.60% | 86.5 |
| **DINAMIT** | 2004 | 674 (Treatment: 332; Control: 342) | MI 6 to 40 days prior to trial start, **LVEF ≤ 35**, std. dev. of RR intervals ≤ 70 msec, mean RR interval ≤ 750 msec, HR >= 80 beats per minute over a 24-hour period | 1.50% | 3.50% | 57.1 |
| **DEFINITE** | 2004 | 458 (Treatment: 229; Control: 229) | DCM, **LVEF ≤ 35**, VFVT, PVC, NSVT | 0.40% | 1.30% | 69.2 |
| **DANISH** | 2016 | 1,116  (Treatment: 556; Control: 560) | CHF, NICM, NYHA functional class II or III, **LVEF ≤ 35**, NT-proBNP > 200pg/mL | 0.10% | 0.20% | 50.0 |
| **Median** |  |  |  |  | **4.87%** |  |

####

#### III Additional performance metrics—Sweden and external validation datasets

The main text focuses on what we consider the most clinically relevant performance metric—sudden cardiac death risk in a discrete high-risk group (i.e., PPV). This section presents a set of more general figures and metrics, as well as discussion of some key comparisons between the Sweden and external validation datasets.

##### III.A Receiver-operating-characteristic, precision-recall, and calibration curves—Sweden and external validation datasets

Each figure below shows one aspect of the model’s performance in the Swedish hold-out set for an outcome—sudden cardiac death (based on death certificates) or VF/VT—and compares it to performance in the relevant external validation dataset: Taiwan for sudden cardiac death (based on detailed chart review) and US for VF/VT. We summarize the results here.

*Calibration*. As shown in the calibration curves, the model is well calibrated in the Swedish validation set for predicting sudden cardiac death. We can also assess calibration in the Taiwanese dataset, which we report naively, treating the case-control sample as a ‘population’ (with a base rate of the outcome is 1.6%, vs. 0.6% in Sweden, driven by the ratio of cases to controls; this is arbitrary, but likely not far from realistic population rates). With these caveats in mind, calibration remains good in Taiwan: the risk increase is linear at 45-degrees, i.e. observed risk rises proportionally and linearly with predicted risk. The intercept is slightly shifted above the 45-degree line, but the 95% confidence intervals include it. Of note, the highest-risk decile in Taiwan is almost 2x as risky as the highest-risk decile in Sweden, but calibration is linear and unaffected.

Because the model was not trained to predict VF/VT, we should not necessarily expect perfect calibration on the 45-degree line for this outcome. However, given the physiological relationship between VF/VT and the prediction target of SCD, we can make some inferences regarding what the graph should look like. First, VF/VT and SCD should be strongly positively correlated: VF/VT causes sudden cardiac death. Reassuringly, we see the monotonic increases in VF/VT vs. predicted risk we expect as a result, i.e., higher risk bins have higher rates of VF/VT. Second, not all VF/VT leads to SCD (these rhythms can self-resolve), so SCD is a subset of VF/VT. Thus we should expect a calibration slope >1, i.e., above the 45-degree line, which is also what we observe (except in the highest decile in Sweden). Again here, we emphasize that the highest-risk decile in the US is 74.3% riskier than the highest-risk decile in Sweden, but US calibration remains approximately linear despite being well outside of the support of the original Swedish distribution.

*Precision and recall*. The precision-recall curve for sudden cardiac death shows clinically high precision (PPV) in the top few percent with reasonable sensitivity. At our preferred threshold (based on ICD RCTs, with median control-arm SCD rates of 4.9%), precision is 7.0% with a sensitivity of 26.5% (see table). As a point of comparison, reduced LVEF has a precision of 4.6% and a sensitivity of 14.8% in our sample.^[[1]](#footnote-1)^ The corresponding F1 scores are 0.111 (95% CI: 0.081, 0.146) for our model vs. 0.070 (95% CI: 0.029, 0.116) for LVEF. In Taiwan (with the same caveats regarding case-control design noted above), the precision-recall curve is at least as good as in Sweden, with very similar PPV, sensitivity, and F1 scores. The difference between the Swedish vs. US precision-recall curves for VF/VT is quite striking - more so than with AUC, because here the base rate affects precision - with US precision and recall both far better than in Sweden. Additional discussion of this in Supplement III.C.

*Outcome: Sudden cardiac death Outcome: VF/VT*

Datasets: Sweden vs. Taiwan Datasets: Sweden vs. US

***Figure III.A.1: Calibration curves***

*Outcome: Sudden cardiac death Outcome: VF/VT*

Datasets: Sweden vs. Taiwan Datasets: Sweden vs. US


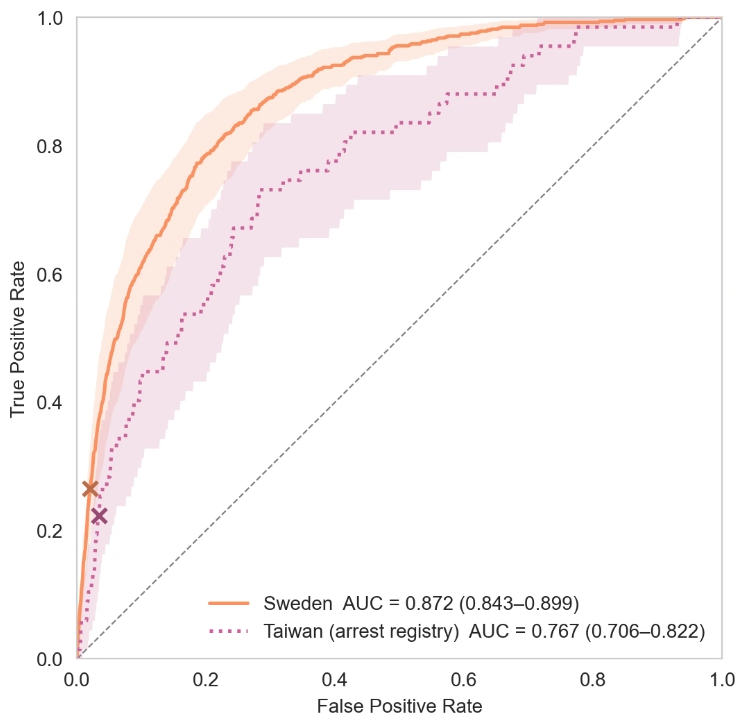

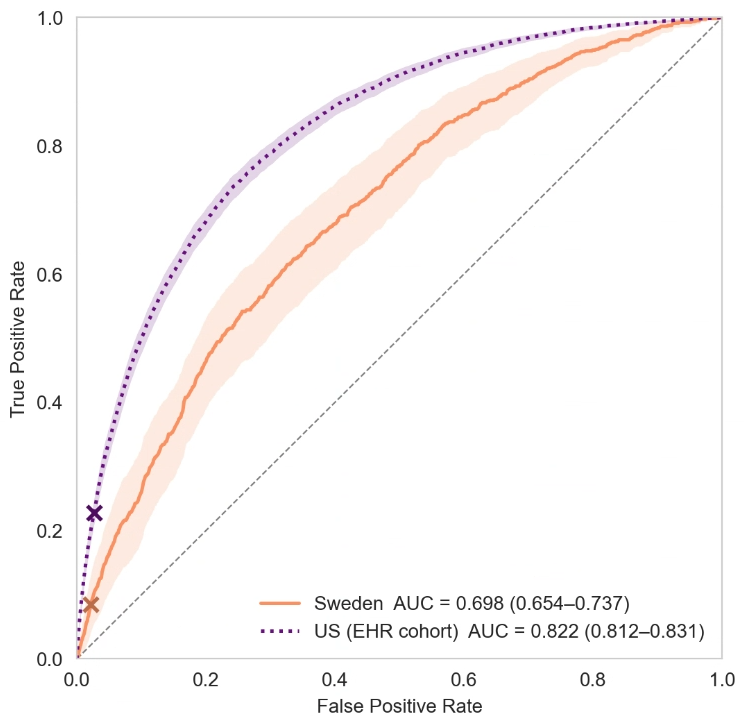


***Figure III.A.2: Receiver-operating-characteristic curves****. Preferred high-risk thresholds marked with x.*

*Outcome: Sudden cardiac death Outcome: VF/VT*

Datasets: Sweden vs. Taiwan Datasets: Sweden vs. US


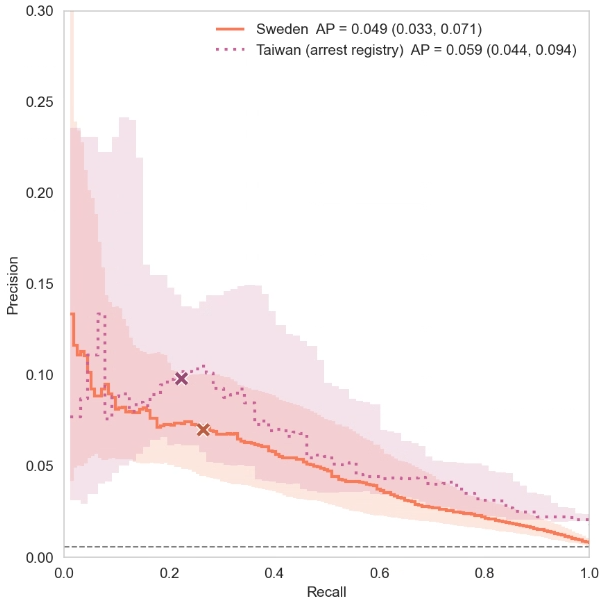

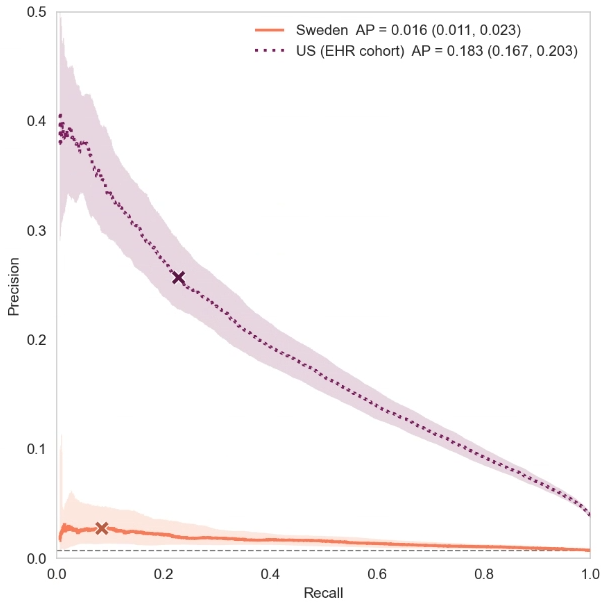


***Figure III.A.3: Precision-recall curves****. Preferred high-risk thresholds marked with x.*

##### III.B Summary statistics on performance—Sweden and external validation datasets

Table III.B.1 shows additional metrics of the model’s performance in the Swedish hold-out set for an outcome—sudden cardiac death (based on death certificates) or VF/VT—and compares it to performance in the relevant external validation dataset: Taiwan for sudden cardiac death (based on detailed chart review) and US for VF/VT.

Overall, results are consistent with the figures above. We add some context regarding Brier scores (also known as mean squared error) by comparing our model to LVEF. We can only do this in the subset of those with LVEF recorded, where our model’s Brier score is 0.011 (95% CI: 0.008, 0.014). This is very close to LVEF, 0.011 (95% CI: 0.008, 0.014; to get LVEF into a probability suitable for Brier calculation, we include it in a logistic regression mode with age and sex in the training set, and calculate Brier score in the validation set,.)

| Outcome | | **Sudden cardiac death** | | **VF/VT** | |
| --- | --- | --- | --- | --- | --- |
| Population | | *Sweden* | *Taiwan*^‡^ | *Sweden* | *US* |
| n | | 113,072 | 4,107 | 106,890 | 251,858 |
| Base rate of outcome | | 0.0058 | 0.0163 | 0.0075 | 0.0384 |
| *Threshold-independent classification metrics* | | | |  |  |
|  | AUC | 0.872 | 0.767 | 0.717 | 0.822 |
|  |  | (0.843, 0.899) | (0.706, 0.822) | (0.676, 0.756) | (0.812, 0.831) |
|  | AUPRC | 0.049 | 0.059 | 0.021 | 0.183 |
|  |  | (0.033, 0.071) | (0.044, 0.094) | (0.014, 0.033) | (0.167, 0.203) |
| *Calibration metrics* | | | |  |  |
|  | Brier Score | 0.006 | 0.016 | 0.008 | 0.036 |
|  |  | (0.005, 0.007) | (0.016, 0.016) | (0.007, 0.009) | (0.035, 0.038) |
|  | Expected Calibration Error (ECE)^†^ | 0.001 | 0.008 | 0.004 | 0.032 |
|  |  | (0.001, 0.002) | (0.007, 0.009) | (0.004, 0.006) | (0.030, 0.034) |
|  | Log- loss | 0.030 | 0.080 | 0.048 | 0.184 |
|  |  | (0.025, 0.035) | (0.074, 0.087) | (0.043, 0.055) | (0.177, 0.192) |
| *Threshold-dependent classification metrics* | | | |  |  |
|  | High risk fraction*** | 0.022 | 0.037 | 0.015 | 0.034 |
|  | PPV | 0.07 | 0.098 | 0.044 | 0.257 |
|  |  | (0.050, 0.094) | (0.062, 0.142) | (0.022, 0.070) | (0.236, 0.280) |
|  | Sensitivity | 0.265 | 0.224 | 0.084 | 0.228 |
|  |  | (0.197, 0.333) | (0.134, 0.328) | (0.045, 0.133) | (0.208, 0.248) |
|  | F1 Score | 0.111 | 0.136 | 0.058 | 0.242 |
|  |  | (0.081, 0.146) | (0.085, 0.197) | (0.030, 0.091) | (0.221, 0.263) |
|  | Accuracy | 0.975 | 0.954 | 0.979 | 0.945 |
|  |  | (0.973, 0.978) | (0.948, 0.959) | (0.977, 0.981) | (0.943, 0.947) |
|  | Balanced Accuracy | 0.622 | 0.595 | 0.535 | 0.601 |
|  |  | (0.588, 0.656) | (0.550, 0.648) | (0.515, 0.559) | (0.591, 0.611) |

Note: 95% CIs are computed via 1,000 bootstrap resamples.
* High-risk threshold is set as described in the main text in the Swedish sample, based on defibrillator trial control group rates of sudden cardiac death. In external validation samples, we set the high-risk threshold to the same absolute value of predicted risk used in the Swedish sample. Sweden VF/VT high-risk fraction is smaller due to censoring (i.e., some of these patients die before one year meaning VF/VT measurement is censored).

^†^ ECE was computed with 10 equal-frequency (quantile) bins.
^‡^ Taiwan dataset has a case–control structure, and statistics are reported naively, i.e., as if the fraction of cases were the population base rate. Thus metrics that involve the base rate—AUPRC, Brier, log-loss, PPV, F1, Accuracy—will be driven by the fraction of cases in the dataset, rather than the true population rate (which is unknown, given the case–control structure).

***Table III.B.1: Additional performance metrics, Sweden, US, Taiwan.***

##### III.C Additional analysis on VF/VT—Sweden and US

While our model is able to discriminate between those who do vs. do not go on to have VF/VT in Sweden (AUC: 0.717, 95% CI: 0.676-0.756), the rates do not increase in risk as strikingly as they do for sudden cardiac death. Indeed, the share of VF/VT as a fraction of all events (VF/VT or sudden cardiac death) decreases in risk.


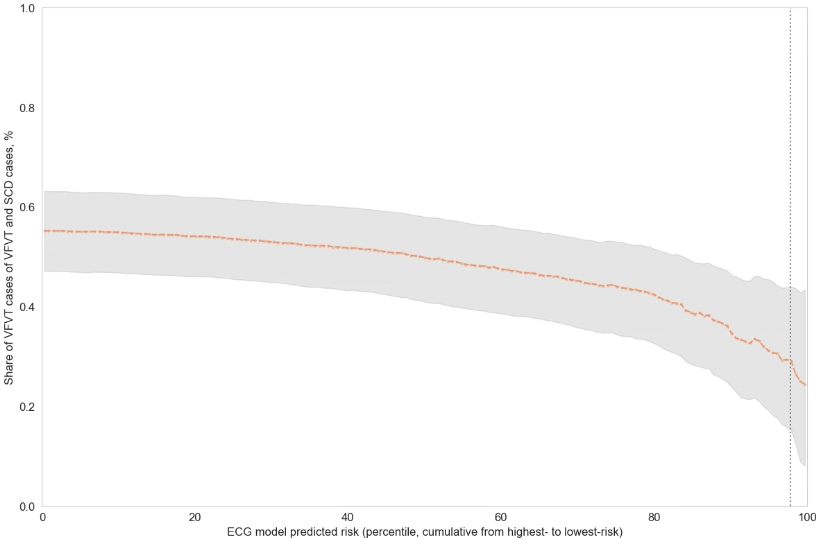


***Figure III.C.1****. Arrhythmia fraction of all arrhythmic or sudden cardiac death events vs. threshold for defining high risk, Sweden.*

As noted in the main text, this may be because not all VF/VT is recorded: doctors may not test patients proportional to risk, or VF/VT in high-risk patients could be censored by death. This could be accentuated if the high-risk cases are more sudden, or more likely to kill the patient as opposed to self-resolving. Alternatively and more pessimistically, the high-risk group could have more non-arrhythmic, non-preventable deaths. (This pattern is not present in the US data, where we could not account for censoring as accurate death information was not available)

To help distinguish between these two possibilities, Figure III.C.2 reproduces a version of Figure 2A that does not account for censoring in measurement of VF/VT due to death. Here, the rate in the highest-risk patients does not rise as steeply in the highest risk percentiles, which suggests that censoring is a major driver of this effect. For example, incidence of documented VF/VT is 3.8% per year (95% CI: 2.2-7.0) after accounting for censoring, but ignoring censoring the rate is far lower: 2.7% (95% CI: 1.4% - 4.5%). Of note, in the main text, the rate of the composite outcome, sudden cardiac death or VF/VT, is calculated in the full population while the rate of VF/VT accounts for censoring by death, resulting in a smaller sample less (if we only include the riskiest 2.2% uncensored observations, the rate is higher: 4.4%, 95% CI: 2.2-7.0).

***Figure III.C.2: Ventricular arrhythmia incidence in high-risk group, vs. threshold for defining high-risk group, without accounting for censoring of VF/VT measurement by death.*** *VF/VT, combined with sudden cardiac death (SCD), in orange, and alone, in purple.*

We reinforce this qualitative observation by designing a simple simulation to explore the amount of censoring needed to produce the pattern we see. We set up a population with Beta-distributed risk *r* of arrhythmia (chosen based on the empirical distribution of risk predictions). In those with arrhythmia, a doctor will test with some probability *t*, revealing VF/VT (*v*=1); otherwise the patient dies (*d*=1) and no VF/VT is recorded.  Censoring is simulated by introducing a small negative correlation between testing *t* and risk *r*. We reproduce Figure 2 in this population below, left, and the share of VF/VT (as a fraction of the total sum of VF/VT and sudden cardiac death) on the right. With no correlation between *r* and *t*, risk increases exponentially (left: *k*=0, top line), and VF/VT share is flat (right: *k*=0, top line). But even small correlations (*k* = -0.025, corresponding to a ~10% decrease in testing in the top risk decile) flattens this at the high end (left), and causes the share of VF/VT to slope down in the highest-risk groups (right), just as we see in the real graph (just above). So censoring is very consistent with the data.

*Rate of VF/VT, high risk group Share of VF/VT in high risk group*


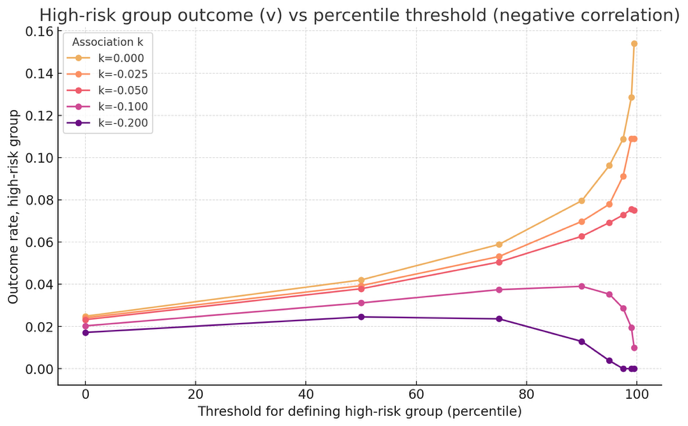

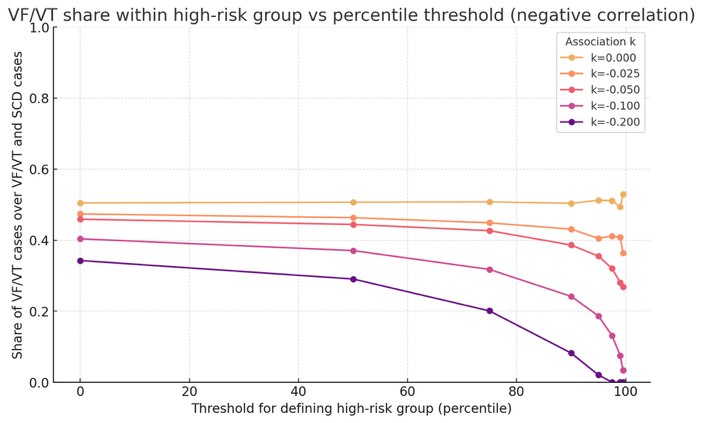


***Figure III.C.3:*** *Simulation: VF/VT outcomes, varying correlation between testing and risk*

The comparison of model performance in Sweden vs. US provides additional context here. While the underlying physiology of VF/VT is likely similar across countries, two key differences between Swedish and US contexts affects the measurement of the outcome. This has several implications for interpretation of model performance metrics and assessment of generalization.

One driver relates to the well-known fact that Swedish patients are healthier to begin with (e.g., life expectancy of 81.7 years vs. 75.8 in the US), and also more likely to have earlier intervention and better disease management thanks to lower barriers to care. This likely compresses the risk distribution captured by ECGs, and with fewer obviously high-risk people, it is more difficult for the model to distinguish future SCDs from others: everyone looks similarly healthy, so the model has to “work harder” to distinguish high- vs. low-risk ECGs. By contrast, the US population is likely to have a wider distribution of risk captured by the ECG, reflecting less equal access to care and outcomes. Thus the same small differences the model picked up on in Sweden are amplified in the US, making it easier to discriminate.

To show this empirically, Table III.C.1, Panel A, shows how the predicted risk of these two populations differ: US mean risk is higher (0.64% vs. 0.38%), which we expect from the higher base rate of VF/VT. Just as importantly, the US risk distribution is wider (SD: 1.85% vs. 1.23%), supporting the idea of differences in distribution, not just mean. In the highest-risk groups, these differences are even more pronounced, e.g., the top decile in the US has 74.3% higher predicted risk than the top decile in Sweden.

|  | **Sweden** | | **US** | |
| --- | --- | --- | --- | --- |
| **Panel A: Predicted risk and observed rate of VF/VT** | | | | |
| *Risk stratum* | *Predicted risk,*  *mean (SD)* | *VF/VT rate* | *Predicted risk,*  *mean (SD)* | *VF/VT rate* |
| Top 2.5% | 6.20 (4.17) | 3.74 | 10.05 (4.14) | 28.17 |
| Top 5% | 4.23 (3.55) | 3.03 | 7.21 (4.11) | 23.01 |
| Top 7.5% | 3.26 (3.19) | 2.43 | 5.71 (3.98) | 19.76 |
| Top 10% | 2.72 (2.94) | 2.36 | 4.74 (3.83) | 17.71 |
| Top 20% | 1.64 (2.35) | 1.88 | 2.84 (3.32) | 12.63 |
| Top 30% | 1.17 (2.02) | 1.53 | 2.01 (2.95) | 9.88 |
| Top 40% | 0.91 (1.81) | 1.32 | 1.55 (2.68) | 8.15 |
| Top 50% | 0.74 (1.66) | 1.18 | 1.26 (2.46) | 6.93 |
| Top 60% | 0.62 (1.53) | 1.08 | 1.06 (2.29) | 6.02 |
| Top 70% | 0.54 (1.44) | 0.98 | 0.91 (2.15) | 5.30 |
| Top 80% | 0.47 (1.35) | 0.90 | 0.80 (2.04) | 4.72 |
| Top 90% | 0.42 (1.28) | 0.83 | 0.71 (1.94) | 4.23 |
| Full population | 0.38 (1.23) | 0.75 | 0.64 (1.85) | 3.84 |
|  |  |  |  |  |
| **Panel B: Regression of observed VF/VT on predicted risk** | | | | |
|  | *Coefficient* | *SE* | *Coefficient* | *SE* |
| Predicted risk | 0.4523*** | 0.0215 | 2.7939 | 0.0199 |
| Constant | 0.0058*** | <0.0001 | 0.0206 | <0.0001 |

***Table III.C.1: Model predicted risk and observed VF/VT rate in Sweden vs. the US****. Panel A shows these two quantities by quantile of predicted risk. Panel B shows the results of a regression of VF/VT on predicted risk.*

Panel B shows the second major driver: testing for VF/VT in Sweden is likely far less common than in the US, conditional on predicted risk. Hospitals receive higher insurance reimbursement rates when VF/VT is present in the US (they are “major complications and comorbidities”), creating strong financial incentives to test, e.g., with Holter monitors, cardiac monitoring during hospitalization, and document. In particular, comparing the regression coefficients, we see that patients in the US are 6.2x (coefficient on risk: 2.79 vs. 0.45) more likely to be coded as having VF/VT, conditional on model predicted risk. (We attempted to produce some empirical results on the frequency of measurement across settings, but unfortunately, ambulatory ECG monitoring in Sweden is either very infrequently recorded in EHRs, or very infrequently done: we were only able to identify a handful of patients with Holter monitors, for example, over the span of our dataset.) This means the VF/VT label is likely *more aligned with the true underlying incidence* of VF/VT in the US, which increases the AUC of the model - which is quite good at predicting arrhythmias, because it was trained on death certificates. As a concrete example, imagine that the model correctly flags a high-risk Swedish patient who goes on to have VF/VT. Because that person is less likely to be tested, the model is more likely to appear “wrong” in Sweden data, decreasing AUC - but in this case, the model prediction was right and the label was wrong. In the US dataset, the same patient is more likely to be tested, and the model is more likely to be vindicated by a recorded episode of VF/VT. This increased testing may also mitigate the censoring of high-risk patients we hypothesize in Sweden.

The net result of these factors is that it is easier for the model to discriminate, and find a high-risk group with high rates of VF/VT, in the US vs. Sweden.

#### IV Additional comparisons of ECG model vs. LVEF

##### IV.A Analysis of mortality with vs. without defibrillator—Sweden, subset of patients under 80 with measured LVEF only

|  | (1) | (2) | (3) | (4) | (5) | (6) |
| --- | --- | --- | --- | --- | --- | --- |
|  | **Sudden cardiac death** | | | **All-cause mortality** | | |
|  | *ECG model* | *LVEF* | *Both* | *ECG model* | *LVEF* | *Both* |
| *Risk indicator variables* | | | | | | |
| ECG high-risk | 0.0668*** (0.003) |  | 0.0604*** (0.003) | 0.2947*** (0.008) |  | 0.2836*** (0.008) |
| LVEF high-risk |  | 0.0371*** (0.002) | 0.0296*** (0.002) |  | 0.0864*** (0.006) | 0.0513*** (0.006) |
| Defibrillator present | -0.004 (0.002)* | -0.0037 (0.002) | -0.0034 (0.002) | -0.0445*** (0.005) | -0.0429*** (0.005) | -0.0543*** (0.005) |
| *Defibrillator x high-risk interactions* | | | | | | |
| Defibrillator x  ECG high-risk | -0.0467*** (0.005) |  | -0.0419*** (0.005) | -0.0942*** (0.013) |  | -0.0974*** (0.014) |
| Defibrillator x  LVEF high-risk |  | -0.0264*** (0.004) | -0.0224*** (0.005) |  | 0.0065 (0.011) | 0.0067 (0.011) |
| Baseline rate | 0.0081*** (0.0007) | 0.0081*** (0.0007) | 0.0059*** (0.0007) | 0.0643*** (0.0016) | 0.0711*** (0.0017) | 0.0606*** (0.0017) |
| *n*= 29,014 for all regressions. All models control for patient age and sex. Standard errors are clustered by patient. Baseline rate combines model intercept with age and sex effects (set to population means; SE accounts for covariances) to estimate outcome rate in low-risk patients without defibrillators. | | | | | | |

***Table IV.A.1: Regression analysis of differences in sudden cardiac death (Cols 1-3) and all-cause mortality (Cols 4-6) for patients with vs. without defibrillators, restricted to those with measured LVEF.*** *Columns 1-3 show coefficients from regressions of sudden cardiac death on high-risk indicators (Col 1: ECG model high-risk group, Col 2: reduced LVEF, Col 3: both), an indicator for whether a defibrillator was implanted, and an interaction term. Columns 4-6 show regressions of all-cause mortality on the same variables. The interaction effect captures the difference in mortality between high-risk patients with vs. without defibrillators.*

##### IV.B Overlap of ECG high-risk group vs reduced LVEF group—US


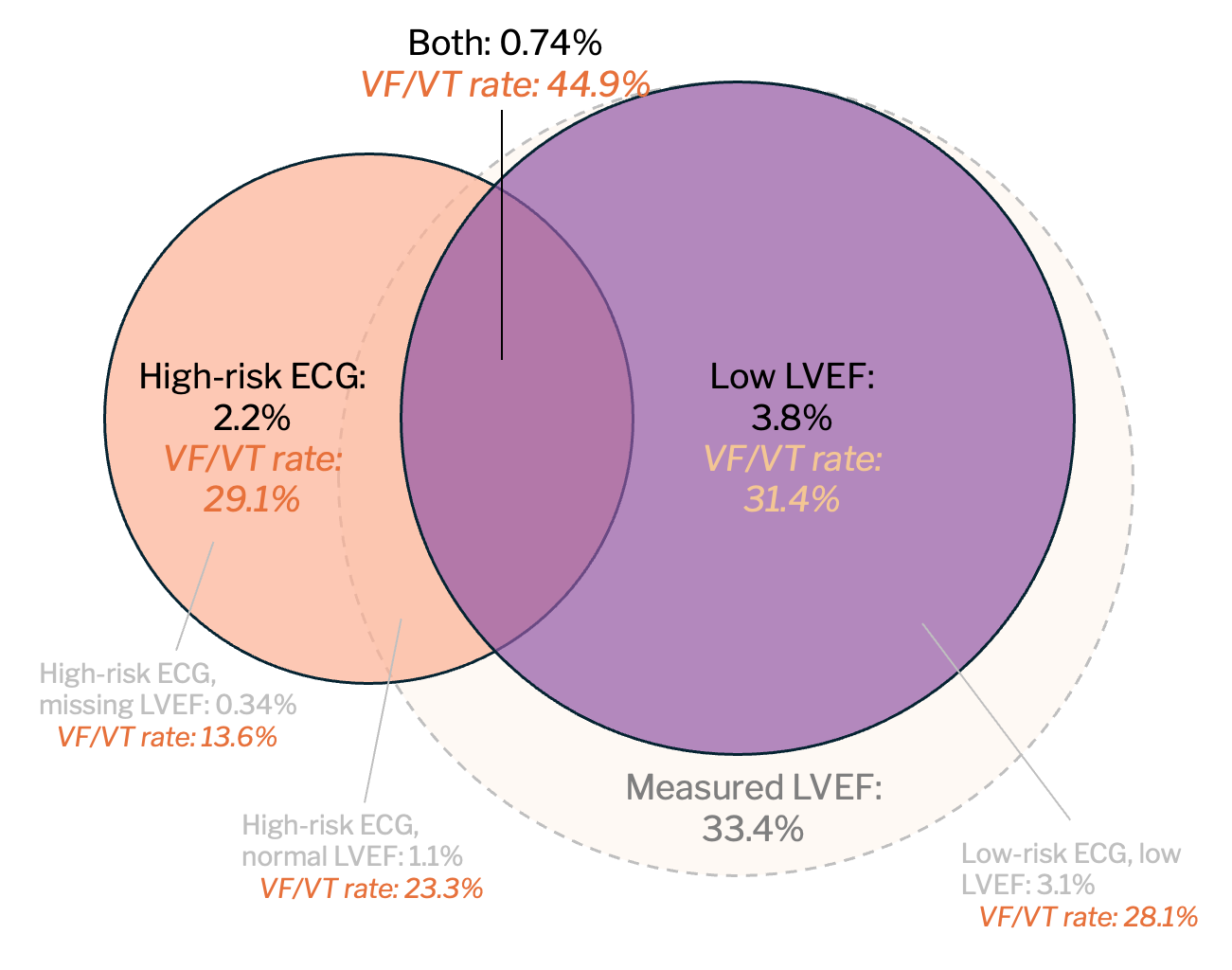


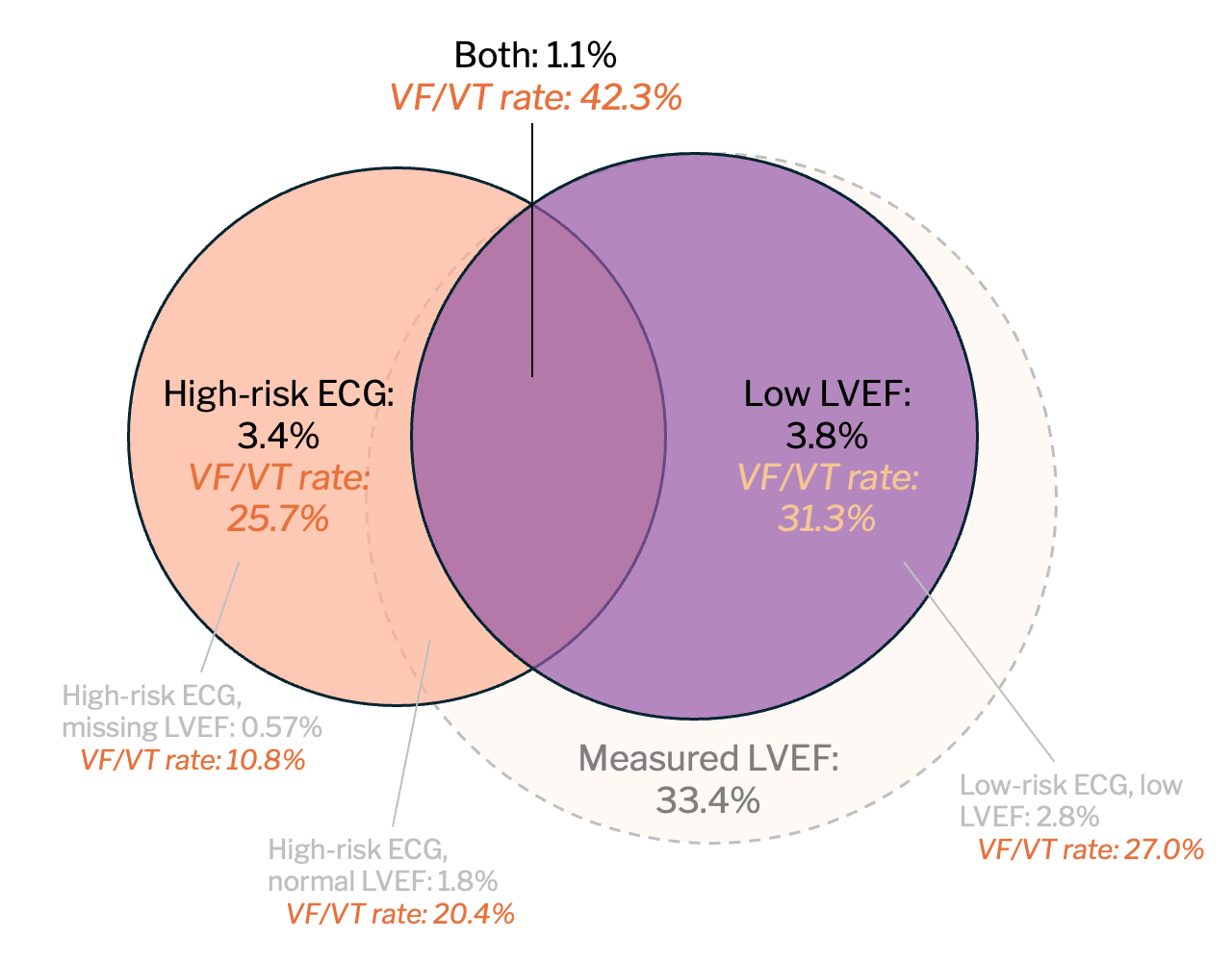


##### ***Figure IV.B.1: Overlap of high-risk ECG group and reduced LVEF group, US.*** *For each set, we show the fraction of the sample accounted for by the set, and the rate of VF/VT in that group. Measured LVEF circle is not drawn to scale. Top—high-risk group defined by relative risk threshold from Sweden (top 2.2%); bottom—absolute risk threshold from Sweden (top 3.4%).*

##### IV.C Rate of key outcomes in subgroups by ECG risk and LVEF—Sweden and US

|  | | *Sweden Cohort*  (N=113,072) | | | *US Cohort*  (N=251,858) | |
| --- | --- | --- | --- | --- | --- | --- |
|  | | Proportion | SCD Rate | VF/VT Rate | Proportion | VF/VT Rate |
| Full Population | | 100% | 0.6  (0.5-0.7)% | 0.8  (0.7-0.9)% | 100% | 3.8  (3.7-4.0)% |
| *LVEF risk predictions* | |  |  |  |  |  |
| Reduced LVEF | | 1.9% | 4.6  (1.9-7.7)% | 2.5  (0.9-4.5)% | 3.8% | 31.4  (28.6-34.2)% |
| Normal LVEF | | 20.6% | 0.8  (0.5-1.2)% | 1.6  (1.1-2.0)% | 35.6% | 6.4  (6.0-6.8)% |
| LVEF unmeasured | | 77.5% | 0.4  (0.3-0.5)% | 0.5  (0.4-0.6)% | 60.6% | 0.6  (0.6-0.7)% |
| *ECG risk predictions* | |  |  |  |  |  |
| High-Risk | | 2.2% | 7.0  (5.0-9.4)% | 4.4  (2.2-7.0)% | 3.4% | 25.7  (23.6-28.0)% |
|  | Reduced LVEF | 0.3% | 10.7  (3.3-20.2)% | 2.0  (0.0-5.0)% | 1.0% | 42.8  (38.3-47.5)% |
|  | Normal LVEF | 0.7% | 6.4  (2.6-11.2)% | 5.0  (0.9-11.6)% | 1.8% | 20.4  (17.8-23.0)% |
|  | LVEF unmeasured | 1.2% | 6.4  (4.0-9.6)% | 4.6  (2.3-7.3)% | 0.6% | 10.8  (7.5-14.8)% |
| Low-Risk | | 97.8% | 0.4  (0.4-0.5)% | 0.7  (0.6-0.8)% | 96.6% | 3.1  (2.9-3.2)% |
|  | Reduced LVEF | 1.6% | 3.4  (1.4-5.8)% | 2.6  (0.9-4.6)% | 2.8% | 27.1  (24.4-29.7)% |
|  | Normal LVEF | 19.9% | 0.6  (0.4-0.9)% | 1.4  (1.1-1.9)% | 33.8% | 5.6  (5.3-6.0)% |
|  | LVEF unmeasured | 76.4% | 0.3  (0.3-0.4)% | 0.5  (0.4-0.5)% | 60.1% | 0.5  (0.5-0.6)% |
| *Combined risk predictions* | | | | | | |
| High-Risk ECG or reduced LVEF | | 3.8% | 5.5  (3.8-7.4)% | 3.5  (2.0-5.2)% | 6.2% | 26.3  (24.4-28.3)% |
| Model Low-Risk and LVEF normal or unmeasured | | 96.2% | 0.4  (0.3-0.5)% | 0.7  (0.6-0.8)% | 93.8% | 2.4  (2.2-2.5)% |
| ***Table IV.C.1: High-risk ECG group and reduced LVEF group, Sweden vs. US.*** *Relative risk threshold (top 2.2%).* | | | | | | |

#### V Generative model and electrophysiological mechanisms

##### V.A Generative model overview

To explore how features of the electrocardiogram (ECG) relate to predicted risk of sudden cardiac death, we combine two components: (i) the risk predictor $M(\cdot)$ trained on real ECGs to estimate the probability of sudden death for a given ECG waveform, and (ii) a new variational autoencoder (VAE) trained to encode and reconstruct realistic ECG waveforms.

**Variational Autoencoder (VAE).** The VAE is comprised of two neural networks. The encoder network $q_{\varphi}(z \mid x)$maps a real patient’s ECG waveform $x$ to a probability distribution over *d*-dimensional latent vector $z$. The decoder network $p_{\theta}\left( x \right| z)$ maps a value of $z$ to a reconstructed ECG waveform $\hat{x}$.

The VAE is trained to maximize the evidence lower bound:

$$\mathcal{L (}\theta, \varphi; x) = \mathbb{E}_{z\sim q_{\varphi}(z \mid x)} [log p_{\theta}(x \mid z)] - KL(q_{\varphi}(z \mid x) \parallel p(z))$$

This trades off two quantities: first, an expected log-likelihood term, which encourages accurate, low-variance reconstructions of $x$ from $z$; and second, a Kullback-Leibler (KL) term that keeps the learned distribution of encoded ECGs close to prior $p(z)$, a multivariate Gaussian over the latent space, in order to create a smooth manifold from which we can sample synthetic ECGs.

**Gradient-Based Morphing.** Working in the latent space of the VAE, we seek small perturbations to a real ECG that increases the risk, following the gradient of *M(·)*, while staying close to the original waveform. Concretely, starting with a real ECG *x_0_*, we pass it through the VAE encoder $q_{\varphi}$to produce latent vector $z_{0}$. We wish to find a nearby latent vector $\tilde{z}$whose decoded waveform $\tilde{x}$ has higher risk than $x_{0}$. Starting at $z_{0}$, we follow the gradient of $M$with respect to the waveform representation $\hat{x}_{0}$, and update $z_{0}$accordingly:

$$z_{t+1} \leftarrow z_{t} + \delta\left( \frac{\partial M}{\partial x} \right)\left( \hat{x}_{t} \right)\left( \frac{\partial p_{\theta}}{\partial z} \right)\left( z_{t} \right)$$

where *δ* is a step size and the procedure runs for *T* steps. This yields latent representations ${\{z}_{0,}z_{1}, \ldots, z_{T}\}$, synthetic ECGs corresponding to each step, with increasing predicted risk. We can then visualize the waveform of each perturbed ECG by passing the new point in latent space through the VAE’s decoder:

$$\hat{x}_{t+1} \leftarrow p_{\theta}(z_{t+1})$$

##### V.B VAE implementation details

The VAE takes as inputs individual ECG beats, which we segment from 10-second ECGs in our sample using standard methods. We use the NeuroKit library’s default peak detection function is used to identify R-peaks within each 12-lead 10-second ECG, and we segment a fixed time window (0.6 sec) around each detected R-peak. We exclude the first and last beat in each ECG, segments containing more than one detected peak, and outlier beats outside in the tails (2.5 percentile) of range, standard deviation, or autocorrelation.

We train the VAE for 100 epochs, using a latent space of *d*=512 dimensions in which each latent variable is represented as a multivariate Gaussian. The encoder employs two one-dimensional convolutional layers (12 channels, kernel size 10) followed by average pooling, ultimately producing the mean and scale parameters that define the Gaussian distribution for sampling the latent vector *z*. This is then passed to a four-layer feed-forward decoder with 100 hidden units per layer, which reconstructs physiologically plausible ECGs from the latent samples. Optimization follows the Evidence Lower Bound (ELBO) using the Adam optimizer and a warmup cosine decay schedule that starts at 1e−7, peaks at 1e−4 after 100 warmup steps, and decays back to 1e−7.

We then prepare a modified version of our full predictive model, trained on individual ECG beats segmented as above. Otherwise the model uses a network architecture and training procedure analogous to the full 10-second ECG model used for the main analysis.

We then randomly sample 56 patient beats for the VAE to encode (given computational constraints, the full pipeline here took hours for each beat). These serve as our point of entry for exploration of the model’s latent space. We sample these 56 beats from a population of grossly normal beats, which we obtain by filtering out beats from patients with ejection fraction below 35, and ECGs with heart rate above 120, P-wave duration exceeding 120 or below 20, QRS duration above 120 or below 20, QTcB greater than 450 or under 350, QTcF greater than 450 or under 350, or RR interval above 1500 or under 350.

Finally, we identify the gradient of predicted risk around each beat, and perturb its latent vector to follow the gradient, similar in spirit to the procedure described in Cohen et al. (2021) and Mullainathan & Ludwig (2024). This produces a higher-risk vector, which we then pass through the decoder to reconstruct, resulting in a counterfactual, higher-risk ECG waveform. The new latent vector is the starting point for another round of perturbation and reconstruction, which we repeat 2000 times, or until the risk of the generated synthetic beat reaches the 90th risk percentile. We employ a learning rate of 1e−2 for these morphing updates. This strategy allows us to produce realistic high-risk waveforms without drifting into implausible or excessively prolonged optimization trajectories.

Further details on this procedure can be found in the accompanying codebase at <https://github.com/alexmschubert/ECG-SCD>.

##### V.C Cardiac physiology underlying the ECG

The heart can be imagined as a tube of toothpaste pointing upward. To empty the tube, it is self-evident that one starts to squeeze from the bottom, then moves progressively upward. Similarly, the heart contracts from bottom (the apex—near the diaphragm) to top (confusingly called the base—near the left shoulder), squeezing blood up into the aorta, the body’s main artery.

This sequence of muscle contraction is coordinated by the heart’s electrical conduction system. Electricity and muscle contraction are directly linked. At rest, heart cells are charged negatively relative to their extracellular environment. A contraction begins when an electrical stimulus is applied to the cell, causing specialized membrane channels to open and allowing positive ions to rush in. This depolarization causes contraction of the muscle cell.

The electrical stimulus for depolarization starts in the heart’s pacemaker cells, located near the base, which spontaneously depolarize at some rate. This triggers rapid depolarization along a ‘highway’ of cells (the fascicles) running from base to apex. Cells adjacent to the highway depolarize first, via slower ‘country roads’—direct cell-to-cell connections—then in turn depolarize their neighbors. At the same time, depolarization along the highway continues rapidly, circling back upward from apex to base. Again, cells adjacent to highways depolarize first, then their neighbors. This tightly choreographed sequence causes muscle contraction to start at the apex and progress to the base, creating upward squeeze of blood.

This sequence means that, at any given time during contraction, some cells are depolarized (more positive) and others are at rest (negative). The boundary between the two differently-charged areas produces a complex surface of small electric dipole moments—vectors with magnitude and direction. The sum of these instantaneous vectors generates an electric field. As depolarization proceeds through the heart muscle, the field changes dynamically over time.

The ECG measures electric potential differences created by this field. Each of its 12 leads are defined by pairs of electrodes placed on the chest, at opposite sides of the heart and roughly equidistant from it. The potential difference between leads is proportional to the instantaneous sum of all dipole moments, along the axis defined by the lead placement points.

##### V.D Axis deviation in high- vs low-risk morphs

A textbook explanation for this is blockage in the left anterior–superior fascicle (LAFB), one of the high-speed conduction pathways that depolarizes a specific population of ventricular cells, starting from anterior and superior in the left ventricle, and progressing posterior and inferior. Blockage in this fascicle results in those cells being depolarized instead via neighboring muscle cells, starting from posterior and inferior, rather than via their direct connection to the blocked fascicle. This changes the average electrical dipole vector during depolarization.

This is not typically described in LAFB; to the extent any rotation is present in LAFB, it should be anterior (clockwise), reflecting delayed depolarization of anterior cells.

Figure V.D.1 show axis rotations visually, with each lead graphed at the angle it measures. For each lead, we sum the voltages recorded during depolarization (the QRS complex), and plot the sum as a vector on the lead’s axis. Compared to low-risk voltages (purple), high-risk voltages (orange) are rotated left (counterclockwise). We emphasize that this coherent physiological pattern was not hard-coded into the model; rather it is an empirical pattern the morphing process identifies in the limb leads (I, II, III, aVL, aVF, aVR).

Panel A: Mean vertical electrical axis for low- (purple) vs. high- (orange) risk morphed beats, calculated by summing all voltages over the QRS complex in the limb leads. If the sum is negative, it is plotted at 180 degrees to the lead. Panel B: Mean horizontal electrical axis for low- (blue) vs. high- (red) risk beats, calculated for QRS complexes in the precordial leads.


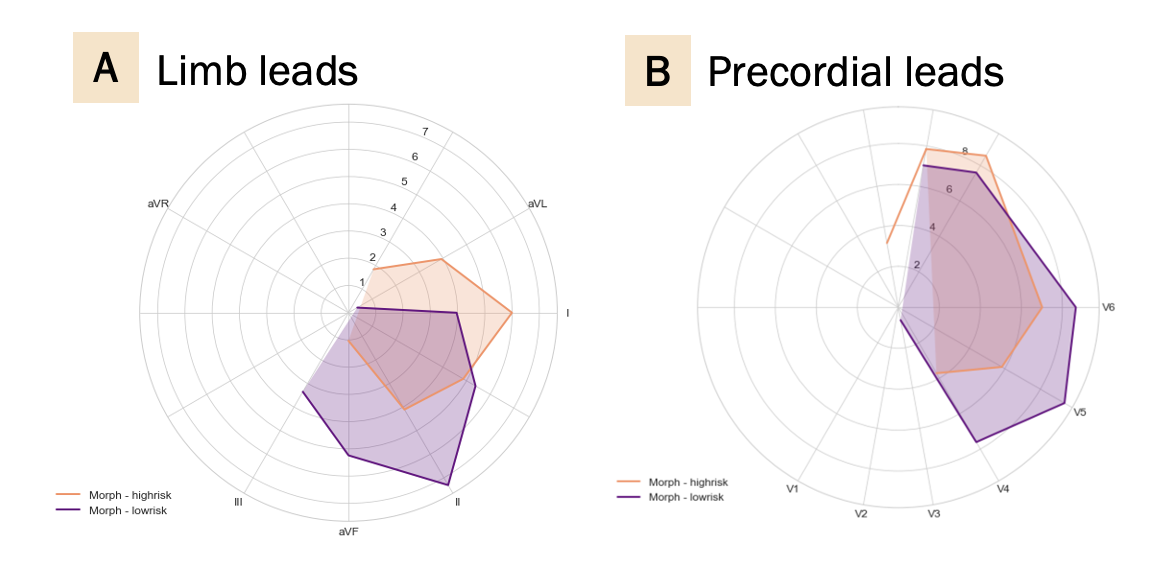


**Figure V.D.1**. Axis rotations.

##### V.E Median waveforms

*High- vs low-risk morph median beat*

*
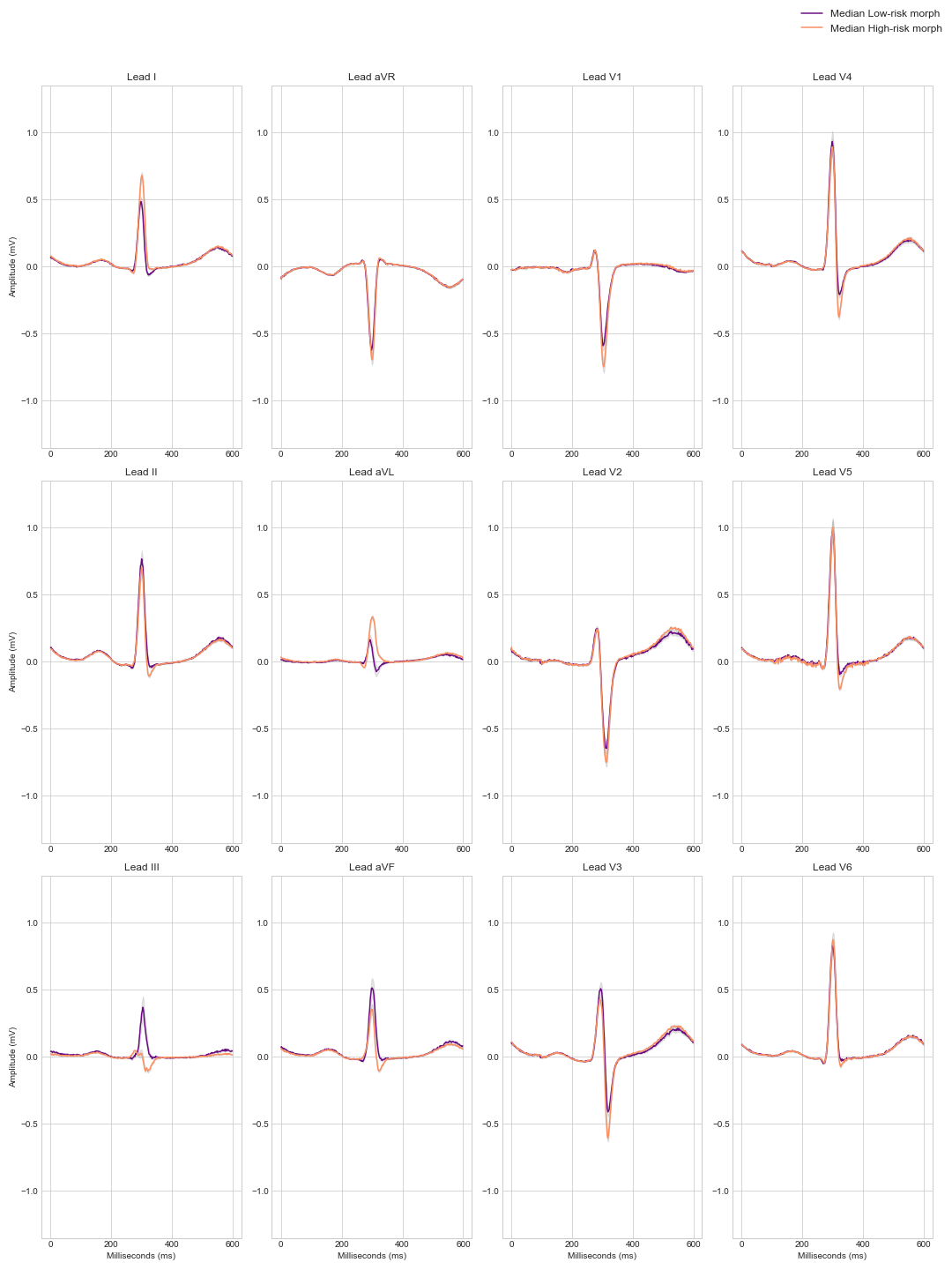
*

*High- vs low-risk real median beat*


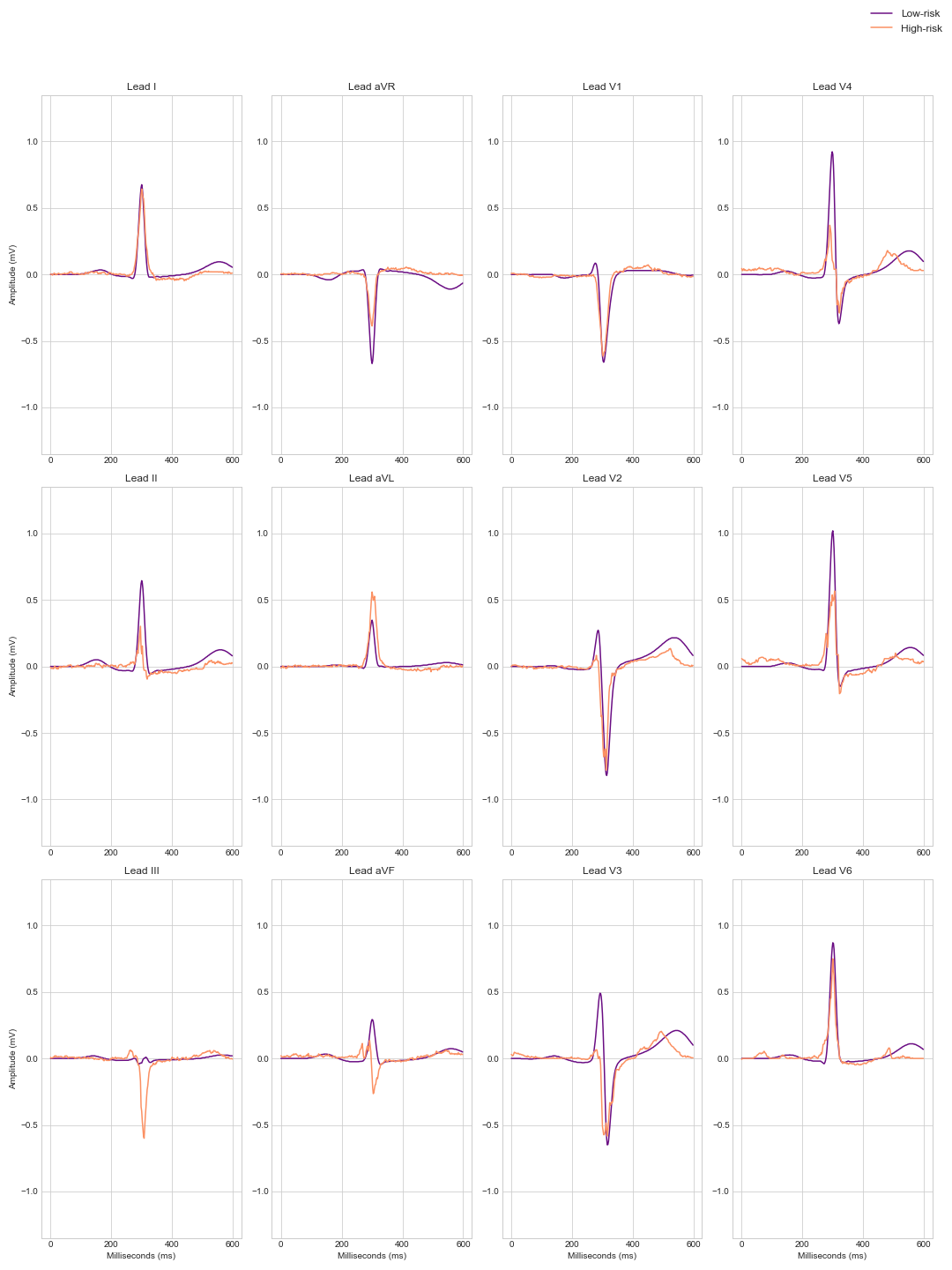


*SCD vs non-SCD patient median beat*


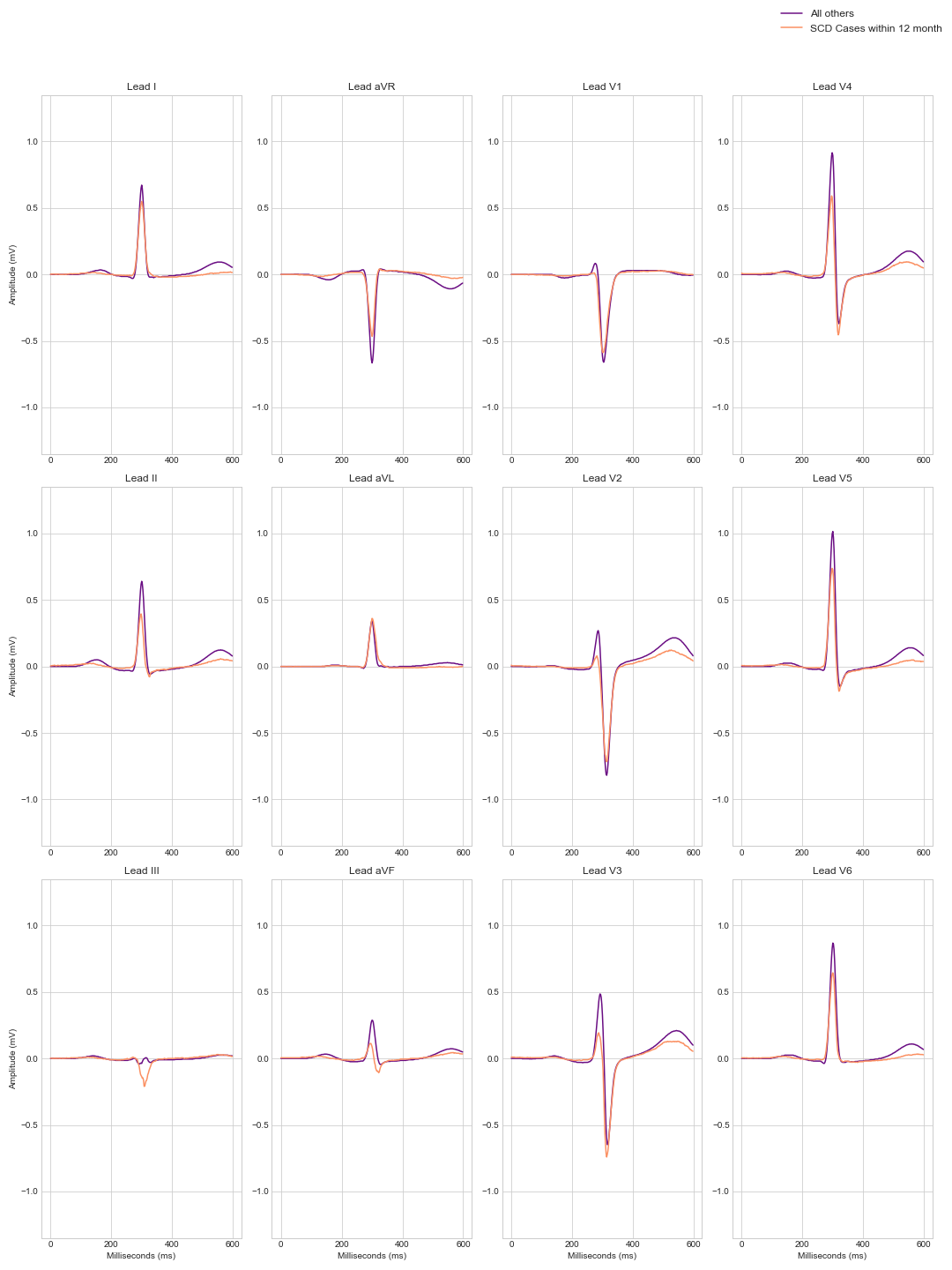


##### V.F Saliency map for ECG waveform

For the same beat shown in the main text, we apply a gradient-based saliency mapping method following Simonyan et al. (2016).^70^ This technique computes the gradient of the predicted class score for sudden cardiac death with respect to the input ECG signal, quantifying the influence of each data point on the prediction. The resulting saliency map highlights the regions of the beat that are most critical for the model's decision. However, while this method indicates which parts of the beat are influential, it does not reveal the specific ECG characteristics driving the classification.


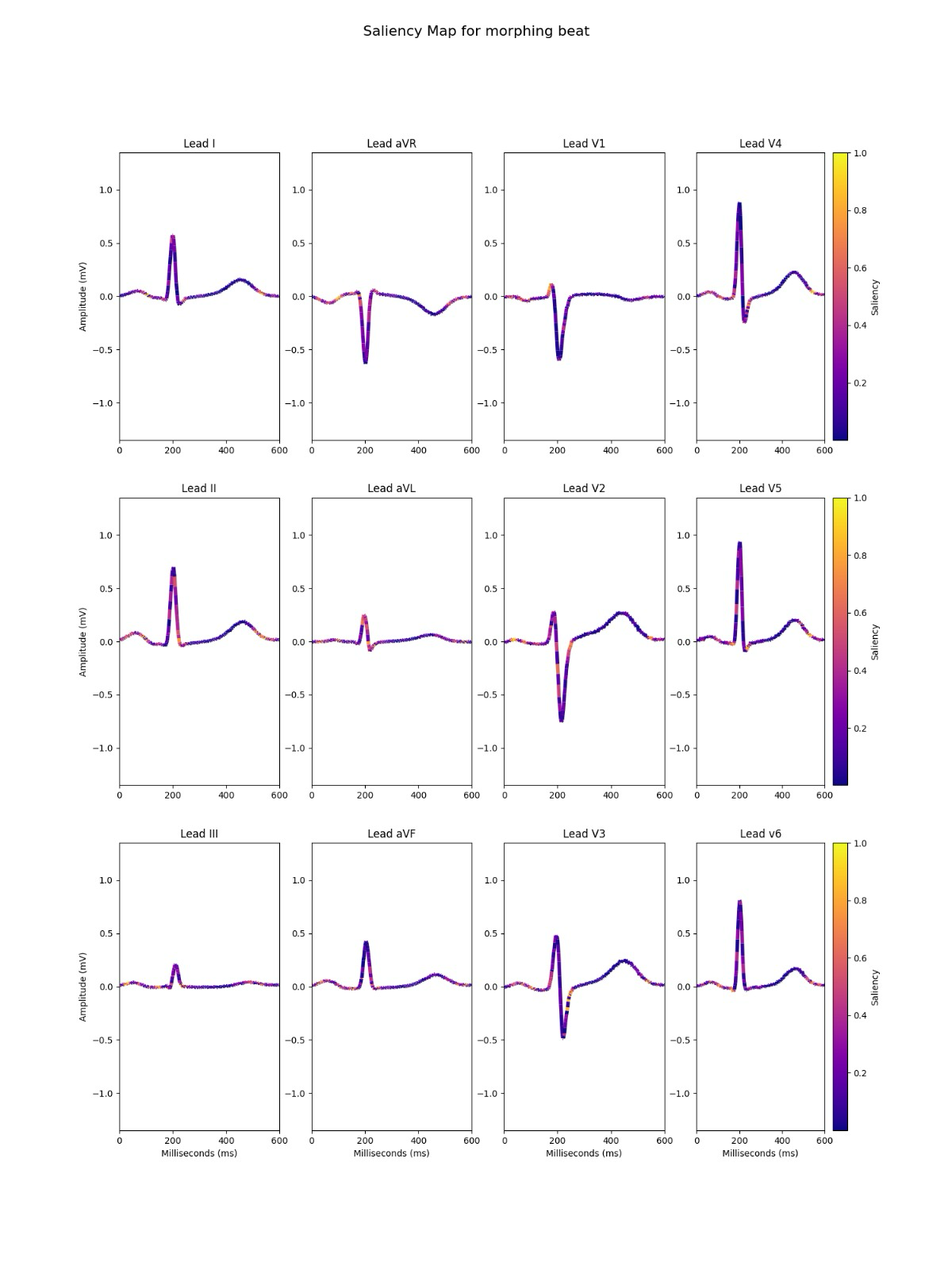


##### V.G Features derived from aVL waveform morphology

Table V.G.1 shows results of OLS regression of the different definitions of sudden cardiac death on the new features we develop based on the novel morphological feature of aVL identified by the predictive model. The first two rows show the coefficients on the features in univariate regression, and the third and fourth rows control for standard quantitative ECG features: heart rate, duration of P wave and QRS complex, frontal and horizontal QRS axis, QT interval (unnormalized and corrected), and an indicator for QTc>500ms.

|  |  | **Dataset and outcome** | | | |
| --- | --- | --- | --- | --- | --- |
|  |  | *Sweden* | | *US* | *Taiwan* |
| *Controls* | *Feature* | *SCD* | *VF/VT* | *VF/VT* | *Arrest, cardiac cause, vs. control* |
| *No controls* | 1st difference | -0.069 *** (0.012) | -0.185*** (0.016) | -0.099*** (0.011) | -0.161***  (0.047) |
|  | 2nd difference | -0.038** (0.014) | -0.1343*** (0.019) | -0.082*** (0.011) | -0.132**  (0.050) |
| *With ECG Controls* | 1st difference | -0.047*** (0.012) | -0.1321*** (0.017) | -0.042*** (0.011) | -0.073  (0.048) |
|  | 2nd difference | -0.015 (0.015) | -0.0521** (0.019) | -0.030** (0.011) | -0.077 (0.050) |
| **p<0.05, **p<0.01, ***p<0.001*  Notes: We drop 654 ECGs in Sweden, where features could not be calculated due to beat segmentation failure. Otherwise, sample size for these datasets is as described in the main text. | | | | | |

***Table V.G.1****. Regression of sudden cardiac death and related outcomes on mean absolute first and second differences from R-peak to QRS-end (lead aVL).*

Table V.G.2 uses Swedish data to compare the coefficients of the first- and second-difference features vs. frontal axis deviation, to investigate how much the new features add to the known feature of left anterior–superior fascicle block. Each row calculates the difference features in one ECG lead. Each pair of columns A and B compare the coefficient of a difference feature (columns A) and the frontal axis feature (columns B). So within each row, each A-B pair shows coefficients from one regression. All regressions control for (but do not show coefficients for) standard quantitative ECG features: heart rate, duration of P wave and QRS complex, frontal and horizontal QRS axis, QT interval (unnormalized and corrected), and an indicator for QTc>500ms. The left four columns use sudden cardiac death as the outcome, and the right four columns use VF/VT as the outcome. The top panel shows the results of a simple regression with all variables in their natural units; and the bottom panel converts the variables into standard deviation units (i.e., each predictor variable is converted to a z-score) in order to directly compare the magnitude of the new variables vs. frontal axis rotation.

|  | **Outcome: SCD** | | | | **Outcome: VF/VT** | | | |
| --- | --- | --- | --- | --- | --- | --- | --- | --- |
|  | *(1A)* | *(1B)* | *(2A)* | *(2B)* | *(3A)* | *(3B)* | *(4A)* | *(4B)* |
|  | *1st diff* | *Front axis^‡^* | *2nd diff* | *Front axis^‡^* | *1st diff* | *Front axis^‡^* | *2nd diff* | *Front axis^‡^* |
| **Natural units** | |  |  |  |  |  |  |  |
| Lead I | -0.0242*** (0.006) | -0.0014* (0.001) | -0.0103 (0.007) | -0.0016** (0.001) | -0.0554*** (0.008) | -0.0059*** (0.001) | -0.0297** (0.009) | -0.0062*** (0.001) |
| Lead II | -0.0233** (0.008) | -0.0016** (0.001) | 0.0335** (0.01) | -0.0021*** (0.001) | -0.0697*** (0.011) | -0.0061*** (0.001) | -0.0074 (0.014) | -0.0066*** (0.001) |
| Lead III | -0.0336*** (0.005) | -0.0012* (0.001) | -0.0214*** (0.006) | -0.0016** (0.001) | -0.0315*** (0.007) | -0.0060*** (0.001) | -0.0153* (0.008) | -0.0064*** (0.001) |
| Lead aVR | -0.0055 (0.008) | -0.0018** (0.001) | 0.061*** (0.011) | -0.0019*** (0.001) | -0.0584*** (0.01) | -0.0064*** (0.001) | -0.0125 (0.015) | -0.0066*** (0.001) |
| **Lead aVL** | **-0.0468*** (0.012)** | **-0.0017** (0.001)** | **-0.0186**  **(0.015)** | **-0.0015** (0.001)** | **-0.1395*** (0.017)** | **-0.0068*** (0.001)** | **-0.0616** (0.019)** | **-0.0062*** (0.001)** |
| Lead aVF | -0.0248*** (0.006) | -0.0015** (0.001) | -0.0113 (0.007) | -0.0017** (0.001) | -0.0564*** (0.008) | -0.0058*** (0.001) | -0.0309** (0.009) | -0.0061*** (0.001) |
| Lead V1 | -0.023*** (0.006) | -0.0015** (0.001) | -0.0175** (0.007) | -0.0018** (0.001) | -0.0257** (0.007) | -0.0064*** (0.001) | -0.0078 (0.009) | -0.0067*** (0.001) |
| Lead V2 | -0.003 (0.009) | -0.0018** (0.001) | -0.0083 (0.011) | -0.0018** (0.001) | -0.0204 (0.012) | -0.0066*** (0.001) | 0.0003 (0.015) | -0.0067*** (0.001) |
| Lead V3 | -0.0029 (0.008) | -0.0018** (0.001) | 0.0283* (0.012) | -0.0018** (0.001) | -0.0076 (0.011) | -0.0067*** (0.001) | 0.0144 (0.016) | -0.0067*** (0.001) |
| Lead V4 | -0.0309*** (0.006) | -0.0013* (0.001) | -0.0137 (0.011) | -0.0016** (0.001) | -0.0305*** (0.009) | -0.0063*** (0.001) | -0.009 (0.014) | -0.0066*** (0.001) |
| Lead V5 | -0.0433*** (0.007) | -0.0007 (0.001) | -0.0255* (0.01) | -0.0013* (0.001) | -0.0813*** (0.009) | -0.0050*** (0.001) | -0.0558*** (0.014) | -0.0060*** (0.001) |
| Lead V6 | -0.0441*** (0.008) | -0.0011 (0.001) | -0.0208 (0.011) | -0.0013* (0.001) | -0.0823*** (0.011) | -0.0060*** (0.001) | -0.0338* (0.015) | -0.0065*** (0.001) |
| **SD units**^†^ | |  |  |  |  |  |  |  |
| Lead I | -0.1022*** (0.025) | -0.0681* (0.027) | -0.0389 (0.025) | -0.0759** (0.027) | -0.2344*** (0.033) | -0.2802*** (0.036) | -0.1119** (0.034) | -0.2934*** (0.036) |
| Lead II | -0.076** (0.026) | -0.076** (0.027) | 0.0869** (0.027) | -0.1002*** (0.027) | -0.227*** (0.035) | -0.2896*** (0.036) | -0.0191 (0.036) | -0.3142*** (0.036) |
| Lead III | -0.16*** (0.025) | -0.0559* (0.027) | -0.0889*** (0.024) | -0.0765** (0.027) | -0.15*** (0.033) | -0.2863*** (0.036) | -0.0638* (0.032) | -0.3075*** (0.036) |
| Lead aVR | -0.0188 (0.026) | -0.087** (0.027) | 0.1535*** (0.028) | -0.0928*** (0.026) | -0.1991*** (0.035) | -0.3046*** (0.036) | -0.0314 (0.038) | -0.3132*** (0.036) |
| **Lead aVL** | **-0.101*** (0.025)** | **-0.0818** (0.027)** | **-0.0348 (0.027)** | **-0.072** (0.027)** | **-0.2815*** (0.033)** | **-0.3249*** (0.036)** | **-0.1155** (0.037)** | **-0.2941*** (0.036)** |
| Lead aVF | -0.1041*** (0.025) | -0.0711** (0.027) | -0.0421 (0.025) | -0.0787** (0.027) | -0.237*** (0.033) | -0.276*** (0.036) | -0.1151** (0.034) | -0.2891*** (0.036) |
| Lead V1 | -0.1027*** (0.025) | -0.0699** (0.027) | -0.0649** (0.024) | -0.085** (0.026) | -0.1148** (0.033) | -0.3032*** (0.036) | -0.0288 (0.032) | -0.3205*** (0.036) |
| Lead V2 | -0.0086 (0.026) | -0.0845** (0.027) | -0.0191 (0.026) | -0.0838** (0.027) | -0.0582 (0.035) | -0.3142*** (0.036) | 0.0006 (0.035) | -0.3195*** (0.036) |
| Lead V3 | -0.0085 (0.025) | -0.0851** (0.026) | 0.0616* (0.026) | -0.0847** (0.026) | -0.0226 (0.034) | -0.3204*** (0.036) | 0.0314 (0.036) | -0.3199*** (0.036) |
| Lead V4 | -0.117*** (0.025) | -0.0613* (0.027) | -0.0325 (0.025) | -0.0749** (0.027) | -0.1156*** (0.033) | -0.2994*** (0.036) | -0.0213 (0.034) | -0.3146*** (0.036) |
| Lead V5 | -0.166*** (0.026) | -0.0344 (0.027) | -0.0633* (0.025) | -0.0622* (0.027) | -0.3114*** (0.035) | -0.2406*** (0.037) | -0.1388*** (0.034) | -0.2881*** (0.037) |
| Lead V6 | -0.1418*** (0.026) | -0.0515 (0.027) | -0.0485 (0.026) | -0.0638* (0.027) | -0.2642*** (0.036) | -0.2854*** (0.036) | -0.0786* (0.036) | -0.3096*** (0.036) |

^†^ Predictors were standardized and then divided by 100. To obtain per 1 SD effects, divide coefficients by 100.

^‡^ QRS Front Axis measurements were divided by 100 for ease of legibility in the top panel.

***Table V.G.2****. Regression of sudden cardiac death and related outcomes on mean absolute first and second differences from R-peak to QRS-end and front axis (columns), across all ECG leads (rows). Top panel shows regression in naural units, bottom panel converts natural units into z-scores for comparability.*

##### V.H Contribution of single ECG leads to the predictive model

While morphological changes in aVL were the most visually striking feature of the high-risk morphs, we also investigated the predictive power of the first- and second-difference features when measured in leads other than aVL, using AUC and PPV. (For PPV, we use a high-risk group comprising 1.8% [2.2%] of the population, as in our main analysis). The results are in the first two rows of each lead subsection of the table below. By a coarse measure, when features are calculated in aVL, they have the highest combined PPVs for sudden death; but (with some exceptions, in aVR and the right-sided precordial leads) most leads generate difference features with roughly similar performance to aVL. This highlights an interesting difference between features noticeable by humans and the model, and suggests that the features are capturing some diffuse process happening throughout the myocardium - albeit more noticeable in some places than others.

Stepping back from the first- and second-difference features individually, we also investigated the predictive power contained in individual ECG leads, to compare them to the 12-lead waveform used in the main analysis. We trained 12 models on single-lead data, and measured their AUCs and PPV, shown in the third row of each lead subsection of the table below. Relative to the full 12-lead model, with AUC 0.84 [0.87], the single-lead predictions range from AUCs of 0.75 (in III) to 0.83 (in I, aVR, V4,V6), indicating that discriminative power is broadly present across all leads. The PPVs for these single-lead models, however, were substantially lower than the full 12-lead model, reflecting worse performance in the tails: using the same 1.8% [2.2%] threshold for defining the high-risk group as in the main analysis, single-lead PPVs range from from 2.1% (in III) [3.6% in III] to 4.9% (V3) [to 5.9% (aVR)], compared to 6.1% [7.0%] for the full 12-lead model. Again, these results suggest that the model finds signal diffusely and throughout many different leads, not just aVL.

It is evident that the first- and second-difference feature AUCs (around 0.60) have far lower performance than the full model AUC (0.84 [0.87]): we emphasize that these features are only a proxy for the morphology visualized by the generative model, which is only a subset of the signal used by the predictive model. As a result, the full model is much richer than any one summary statistic, and the AUC is much higher. The features are highlighted not for their predictive power (though it’s important to establish that they have some predictive power alone), but rather as a way to generate physiological hypotheses.

| *Lead* | *Feature* | *AUC (95  % CI)* | *SCD rate, high-risk* | *Lead* | *Feature* |  | *AUC (95  % CI)* | | *SCD rate, high-risk* | |  |
| --- | --- | --- | --- | --- | --- | --- | --- | --- | --- | --- | --- |
| I | 1st difference | 0.61 (0.56, 0.66) | 0.015 | V1 | 1st difference | | | 0.61 (0.56, 0.66) | | 0.005 | |
|  | 2nd difference | 0.55 (0.50, 0.61) | 0.009 |  | 2nd difference | | | 0.58 (0.54, 0.63) | | 0.009 | |
|  | *Single-lead model* | 0.83 (0.80, 0.86) | 0.045 |  | *Single-lead model* | | | 0.78 (0.73, 0.83) | | 0.044 | |
| II | 1st difference | 0.59 (0.53, 0.64) | 0.014 | V2 | 1st difference | | | 0.55 (0.51, 0.60) | | 0.005 | |
|  | 2nd difference | 0.51 (0.45, 0.57) | 0.01 |  | 2nd difference | | | 0.53 (0.48, 0.59) | | 0.015 | |
|  | *Single-lead model* | 0.80 (0.76, 0.85) | 0.048 |  | *Single-lead model* | | | 0.82 (0.79, 0.85) | | 0.053 | |
| III | 1st difference | 0.61 (0.57, 0.65) | 0.005 | V3 | 1st difference | | | 0.53 (0.49, 0.59) | | 0.008 | |
|  | 2nd difference | 0.57 (0.53, 0.62) | 0.01 |  | 2nd difference | | | 0.49 (0.45, 0.54) | | 0.013 | |
|  | *Single-lead model* | 0.75 (0.70, 0.79) | 0.036 |  | *Single-lead model* | | | 0.81 (0.78, 0.84) | | 0.046 | |
| aVR | 1st difference | 0.64 (0.59, 0.68) | 0.007 | V4 | 1st difference | | | 0.59 (0.54, 0.64) | | 0.011 | |
|  | 2nd difference | 0.55 (0.50, 0.60) | 0.011 |  | 2nd difference | | | 0.53 (0.49, 0.57) | | 0.012 | |
|  | *Single-lead model* | 0.83 (0.79, 0.87) | 0.059 |  | *Single-lead model* | | | 0.83 (0.79, 0.86) | | 0.051 | |
| aVL | 1st difference | *0.61 (0.56, 0.65)* | *0.013* | V5 | 1st difference | | | 0.61 (0.55, 0.67) | | 0.017 | |
|  | 2nd difference | *0.54 (0.48, 0.59)* | *0.009* |  | 2nd difference | | | 0.56 (0.51, 0.62) | | 0.013 | |
|  | *Single-lead model* | *0.79 (0.75, 0.82)* | *0.037* |  | *Single-lead model* | | | 0.82 (0.77, 0.86) | | 0.053 | |
| aVF | 1st difference | 0.61 (0.56, 0.66) | 0.015 | V6 | 1st difference | | | 0.63 (0.58, 0.69) | | 0.02 | |
|  | 2nd difference | 0.55 (0.50, 0.61) | 0.009 |  | 2nd difference | | | 0.58 (0.53, 0.64) | | 0.013 | |
|  | *Single-lead model* | 0.77 (0.72, 0.82) | 0.039 |  | *Single-lead model* | | | 0.83 (0.79, 0.86) | | 0.047 | |

| *Lead* | *Feature* | *AUC (95  % CI)* | *SCD rate, high-risk* | *Lead* | *Feature* |  | *AUC (95  % CI)* | | *SCD rate, high-risk* | |  |
| --- | --- | --- | --- | --- | --- | --- | --- | --- | --- | --- | --- |
| I | 1st difference | 0.58 (0.55, 0.61) | 0.011 | V1 | 1st difference | | | 0.59 (0.56, 0.62) | | 0.014 | |
|  | 2nd difference | 0.60 (0.57, 0.62) | 0.013 |  | 2nd difference | | | 0.60 (0.57, 0.63) | | 0.01 | |
|  | *Single-lead model* | *0.79 (0.77, 0.81)* | *0.034* |  | *Single-lead model* | | | *0.80 (0.78, 0.82)* | | *0.034* | |
| II | 1st difference | 0.53 (0.50, 0.56) | 0.01 | V2 | 1st difference | | | 0.54 (0.51, 0.56) | | 0.009 | |
|  | 2nd difference | 0.58 (0.55, 0.61) | 0.013 |  | 2nd difference | | | 0.52 (0.49, 0.55) | | 0.004 | |
|  | *Single-lead model* | *0.82 (0.80, 0.84)* | *0.036* |  | *Single-lead model* | | | *0.80 (0.78, 0.82)* | | *0.034* | |
| III | 1st difference | 0.61 (0.58, 0.63) | 0.014 | V3 | 1st difference | | | 0.50 (0.47, 0.52) | | 0.006 | |
|  | 2nd difference | 0.63 (0.60, 0.65) | 0.012 |  | 2nd difference | | | 0.52 (0.49, 0.54) | | 0.006 | |
|  | *Single-lead model* | *0.78 (0.76, 0.79)* | *0.021* |  | *Single-lead model* | | | *0.82 (0.80, 0.83)* | | *0.041* | |
| aVR | 1st difference | 0.49 (0.46, 0.51) | 0.006 | V4 | 1st difference | | | 0.56 (0.53, 0.58) | | 0.009 | |
|  | 2nd difference | 0.55 (0.52, 0.57) | 0.005 |  | 2nd difference | | | 0.59 (0.57, 0.62) | | 0.008 | |
|  | *Single-lead model* | *0.82 (0.80, 0.83)* | *0.038* |  | *Single-lead model* | | | *0.82 (0.80, 0.83)* | | *0.041* | |
| **aVL** | **1st difference** | **0.58 (0.56, 0.61)** | **0.014** | V5 | 1st difference | | | 0.59 (0.57, 0.62) | | 0.013 | |
|  | **2nd difference** | **0.58 (0.55, 0.60)** | **0.016** |  | 2nd difference | | | 0.62 (0.60, 0.65) | | 0.016 | |
|  | ***Single-lead model*** | ***0.79 (0.77, 0.81)*** | ***0.030*** |  | *Single-lead model* | | | *0.82 (0.80, 0.83)* | | *0.047* | |
| aVF | 1st difference | 0.58 (0.55, 0.61) | 0.012 | V6 | 1st difference | | | 0.57 (0.54, 0.60) | | 0.014 | |
|  | 2nd difference | 0.60 (0.57, 0.63) | 0.013 |  | 2nd difference | | | 0.60 (0.57, 0.63) | | 0.012 | |
|  | *Single-lead model* | *0.78 (0.76, 0.80)* | *0.033* |  | *Single-lead model* | | | *0.82 (0.80, 0.84)* | | *0.037* | |

***Table V.H.1****. AUC and PPV of mean absolute first and second differences from R-peak to QRS-end, as well as single-lead model, using individual ECG leads .*

##### V.I Features derived from aVL waveform morphology

We next show that this new feature is easily visible in real ECGs. Panel A shows four median beats in the highest-risk percentile of both first and second difference over the interval from R-peak to end of QRS. Panel B shows median beats from age- and sex- matched controls, drawn from the 25th to 75th percentiles of first and second difference features, for comparison.


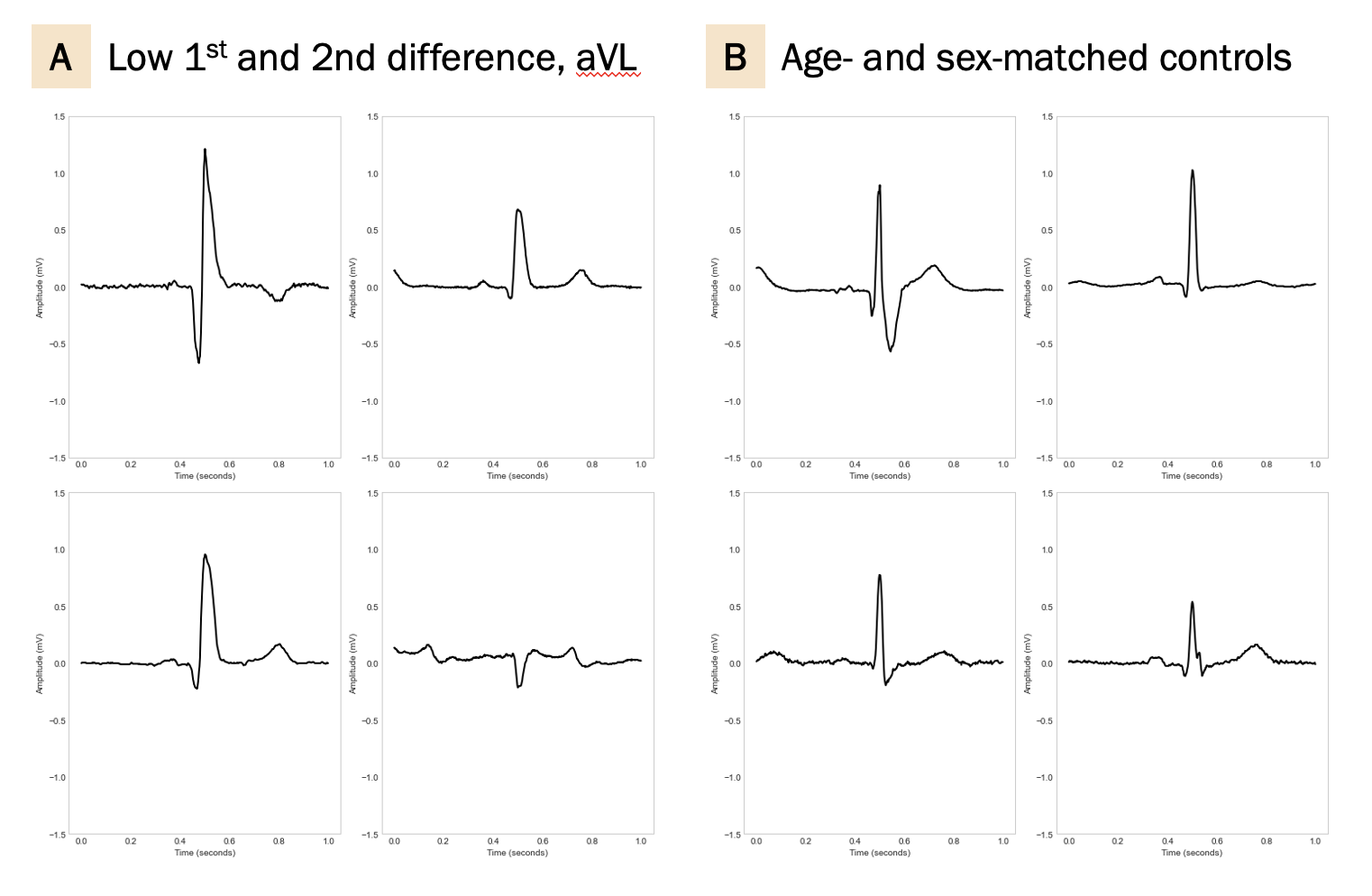


#### VI Hypothesis generation

##### VI.A Stylized model of conduction in cardiac cells

Consider a highly stylized model of the phenomenon we study: a uniform sheet of cells, each electrically connected to their neighbors. For simplicity, cells are square, equally sized, and arranged in a simple grid with connections to four neighbors (vertical and horizontal). Each cell has some probability of depolarizing, which increases in the number of neighbors that are depolarized.

At baseline, we begin depolarization at the top left corner of the sheet, and measure the average angle of the dipole between depolarized and resting cells as depolarization progresses, as if using an ECG lead oriented from left to right.


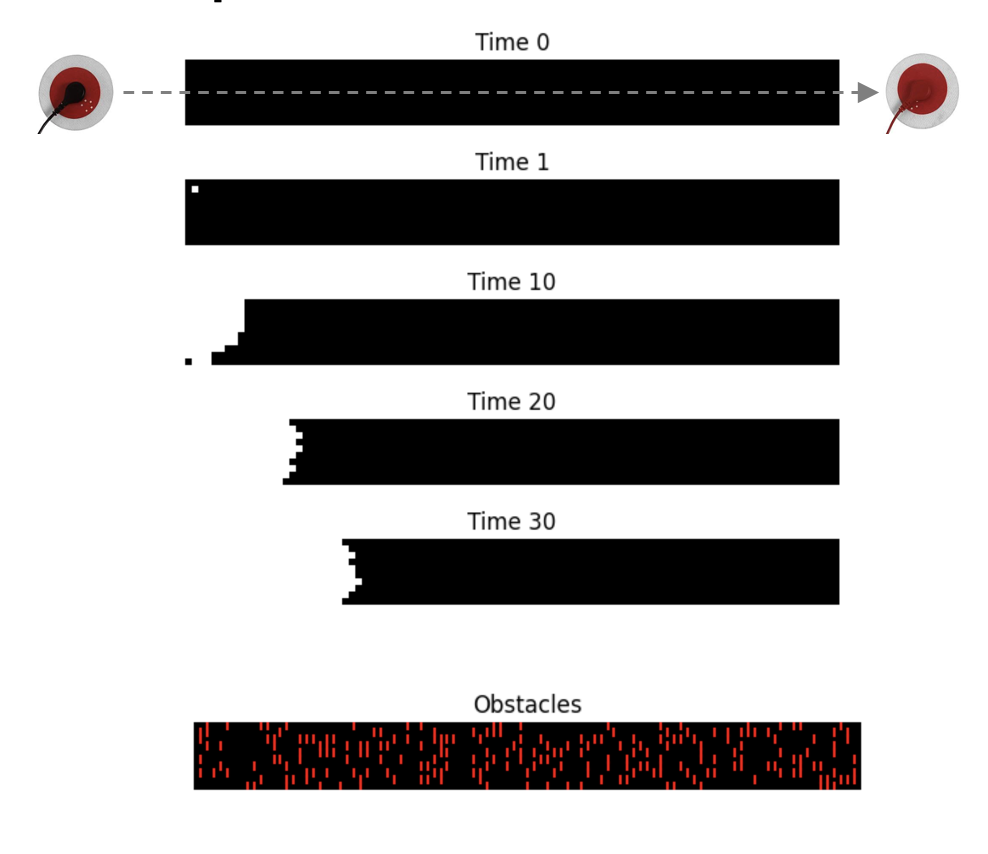


**Figure VI.A.1**. Schematic depiction of depolarization proceeding from left to right.

Next, we add a small area of electrically inert tissue, at a random location in the sheet. The wave of advancing depolarization will be forced to split around this obstacle, changing the orientation of the dipole to an angle more orthogonal to the lead. Now, randomly distribute additional obstacles in this sheet. Conduction will repeatedly split, causing the voltages recorded by the lead to become increasingly orthogonal, similar to scattering phenomena seen in other media. The Figure below illustrates how the vector of depolarization evolves in this simple simulation, varying the density of obstacles to conduction (alpha). As density increases past a critical level, the vector becomes increasingly orthogonal to the lead over time.


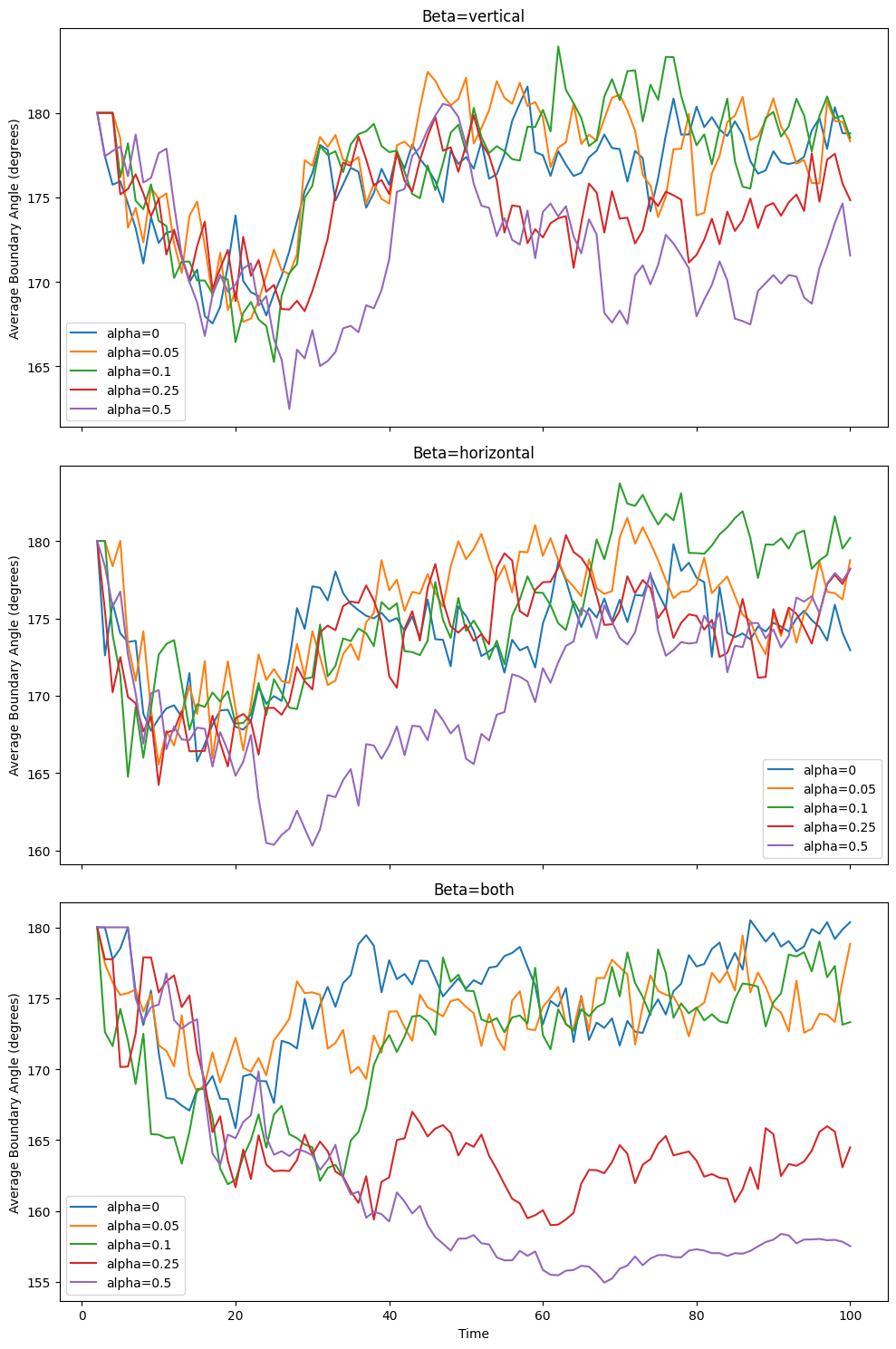


**Figure VI.A.2**. Average boundary angle separating depolarized vs. repolarized cells, analogous to dipole moment, varying the density of obstacles to conduction (alpha).

To summarize, diffusely and randomly distributed obstacles to conduction could alter the average dipole angle of the heart during depolarization, and these changes would compound over time as obstacles progressively divert the wave of depolarization. We would thus see an increasingly orthogonal angle as depolarization progresses, and these changes would be seen most clearly in lead aVL, which is closest to the most distal portions of the heart.

##### VI.B Cardiac MRI data

Several pathophysiological processes in the heart may create such obstacles to conduction. But there is a limit to how much we can infer without more detailed empirical evidence. Thanks to our linkage of ECGs to the universe of EHR data, we can identify patients who have had detailed studies of cardiac physiology in the course of their normal medical care, and correlate these to the algorithm’s predictions. In particular, we identify 98 patients in the validation set who had cardiac MRIs at some point; 88 of these were within 30 days of the ECG. We then selected all 9 patients in the top 10% of the algorithm’s predicted risk based on their ECG closest in time to the MRI, and randomly drew 15 patients with lower predicted risk for detailed review. A cardiologist with experience reading cardiac MRIs reviewed all of these sequences, blinded to both the algorithm’ prediction and patient outcomes, with instructions to focus on abnormalities that might be linked to sudden cardiac death.

The 9 patients in the top 10% of the algorithm’s predicted risk had a significantly higher prevalence of late gadolinium enhancement (LGE) than the 15 lower-risk patients. This finding represents differences in volume or uptake of contrast within the extracellular space of the myocardium. The distribution of LGE in these patients was diffuse and somewhat subtle throughout the left ventricle in particular. Such widespread changes in the extracellular space provide a basis for the hypothesized obstacles to cell-to-cell transmission of depolarization. In particular, the pattern of LGE is consistent with myocardial fibrosis, which is known to slow and redirect conduction through the myocardium and could be expected to produce a ECG pattern similar to that seen in high-risk patients. High-risk patients also had a higher prevalence of LV dysfunction and depressed LVEF, as expected.


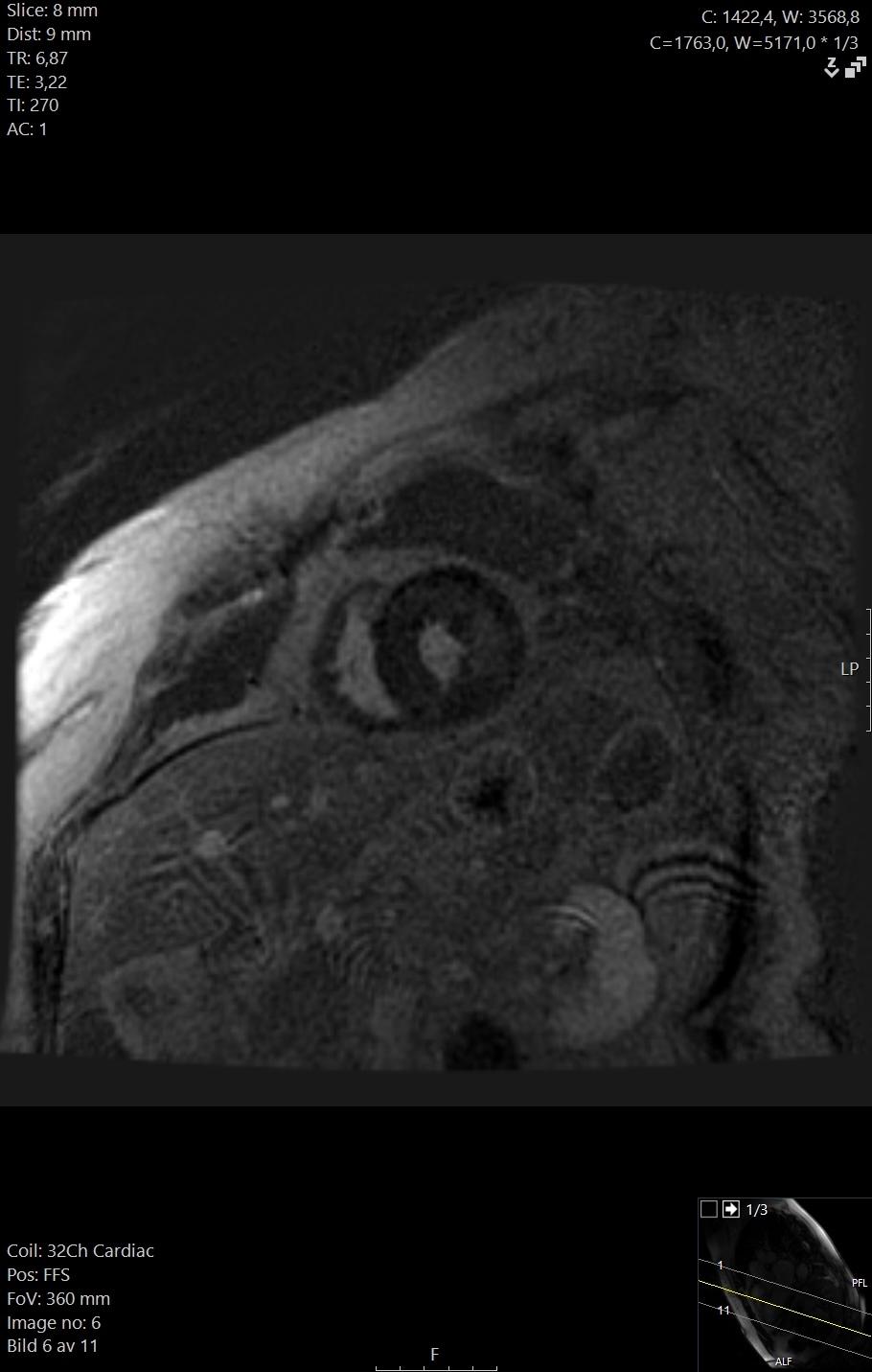


***Figure VI.B.1.*** *cMRI slice from a representative high-risk patient showing LGE*

While fibrosis has been identified as a potential risk factor for sudden cardiac death, the obstacles to diagnosis are considerable: endomyocardial biopsy is the only gold standard, and studies comparing cMRI to biopsy show that LGE is often absent in diffuse fibrosis. The difficulties in studying fibrosis have led clinicians to place low importance on its importance. As one metric of this disregard, we reviewed the cardiologists’ notes for the 3 patients with LGE. In none of the 3 cases was the finding even commented on.


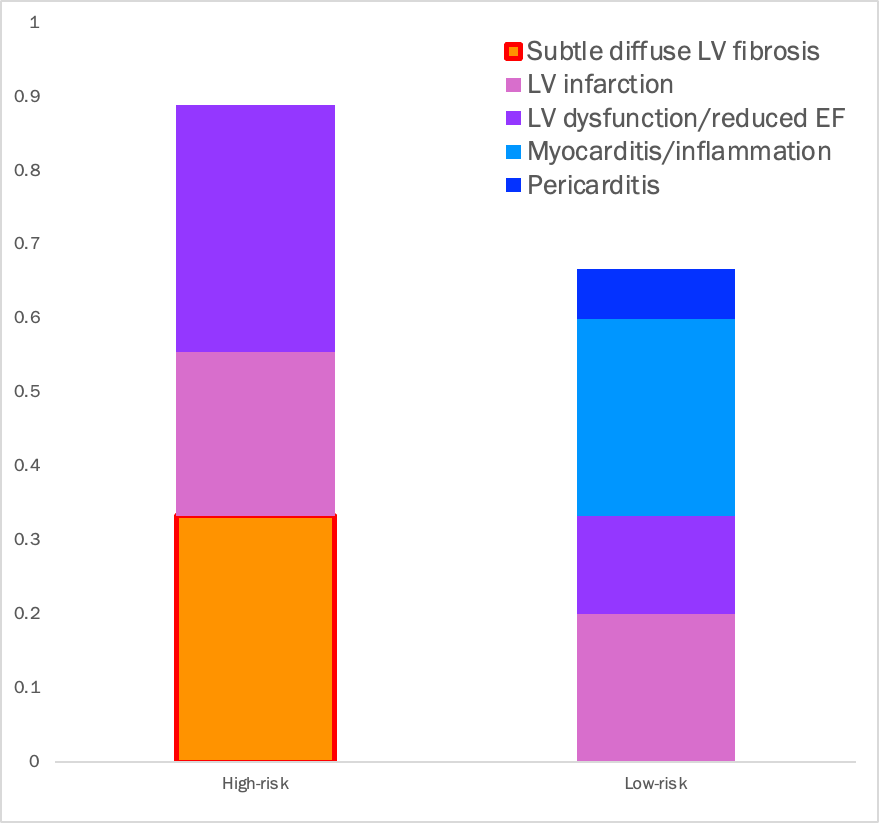


| **Variable** | **Bottom 90% mean** | **Top 10%**  **mean** | **Difference** | **Difference CI (95%)** | **p-value** |  |
| --- | --- | --- | --- | --- | --- | --- |
| Subtle diffuse LV fibrosis | 0.000 | 0.333 | 0.333 | 0.0697 – 0.5970 | 0.0156 |  |
| LV infarction | 0.200 | 0.222 | 0.022 | -0.3486 – 0.3930 | 0.9022 |  |
| LV dysfunction/ reduced EF | 0.133 | 0.333 | 0.200 | -0.1602 – 0.5602 | 0.2619 |  |
| Myocarditis/ inflammation | 0.267 | 0.000 | -0.267 | -0.5860 – 0.0526 | 0.0973 |  |
| Pericarditis | 0.067 | 0.000 | -0.067 | -0.2468 – 0.1134 | 0.4509 |  |

***Figure VI.B.2: Comparison of cMRI findings, high-risk (top 10%) vs. low-risk patients (bottom 90%).*** *Graph above, accompanying data table below shows category comparison.*

#### VII Dataset and key variable construction

##### VII.A Dataset construction—Sweden

Immediately after creation of the full dataset of 441,614 ECGs (from 124,918 patients) linked to health record data and death certificates, we created strict random splits at the patient level (not the ECG level) to safeguard against overfitting. In particular, our first step was to create a data lock-box by randomly sampling 40% of patients and all their ECGs. All authors agreed that the lock-box would not be touched until provisional editorial acceptance of the manuscript, to ensure that final results produced on the lock-box were truly out-of-sample and not over-fit.

For the manuscript preparation, submission, and peer review process, we made exclusive use of the remaining 60% sample, which was immediately split in half at the patient level. One half (131,274 ECGs from 37,556 patients) was used to train the model, after removing 10,052 ECGs from 1045 patients with prior defibrillator placement: we wish the model to learn about risk in the absence of treatment (these patients were not removed from the validation set). The other half (131,280 ECGs from 37,601 patients) formed a validation set in which all results were produced; most results came from the subset of 89,414 ECGs done before age 80 in 29,108 patients. Figure VII.A.1 shows the splits used for this part of the process.


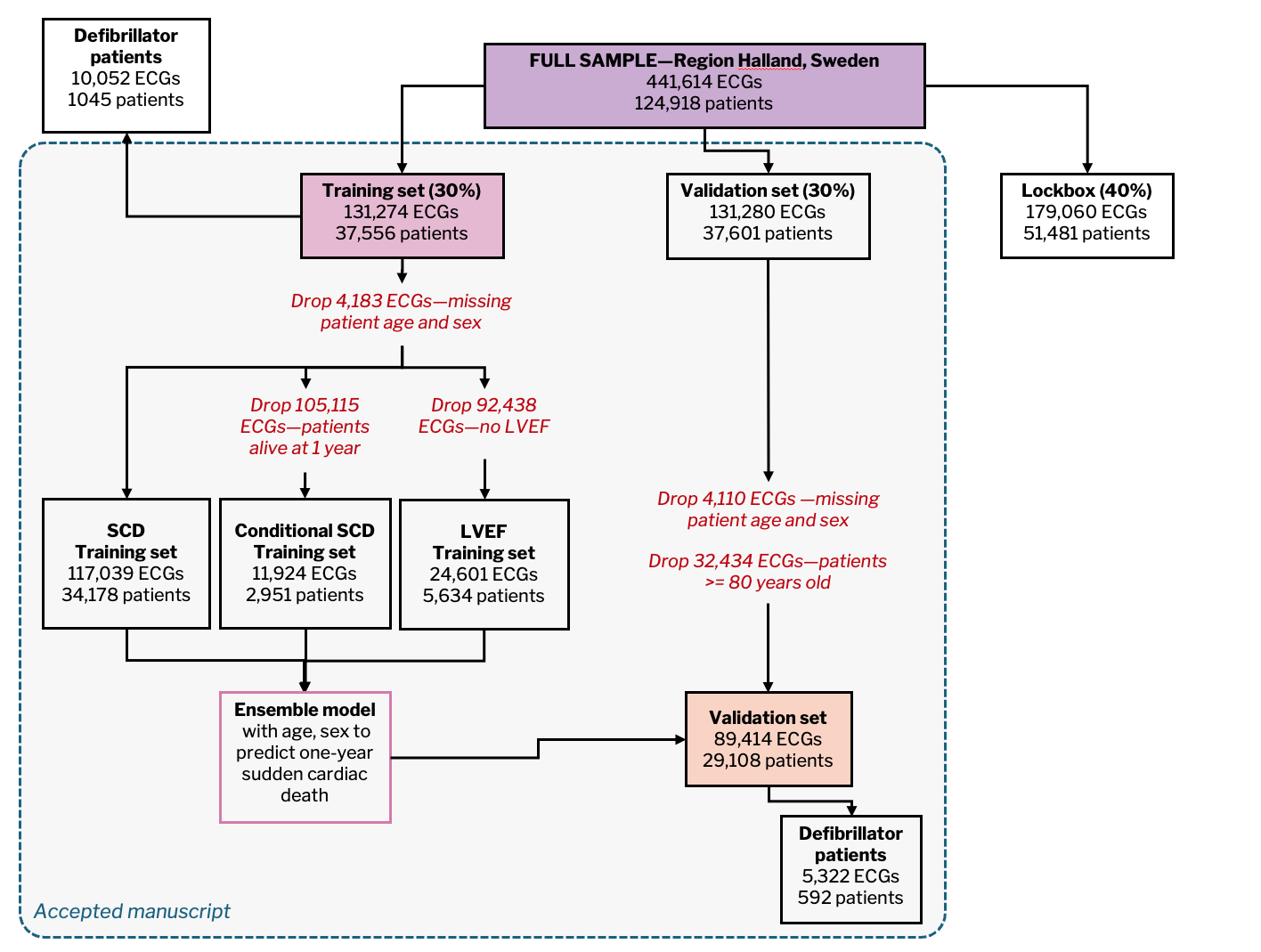


***Figure VII.A.1****: Data splits used from initial manuscript preparation to provisional editorial acceptance.*

Upon acceptance of the manuscript, we retrained the model on the 60% of data we had accessed, 262,554 ECGs from 75,157 patients, and applied the resulting model with no modification to generate predictions in the 40% lock-box: 179,060 ECGs from 51,481 patients.^[[2]](#footnote-2)^ Those results are shown in the main manuscript.


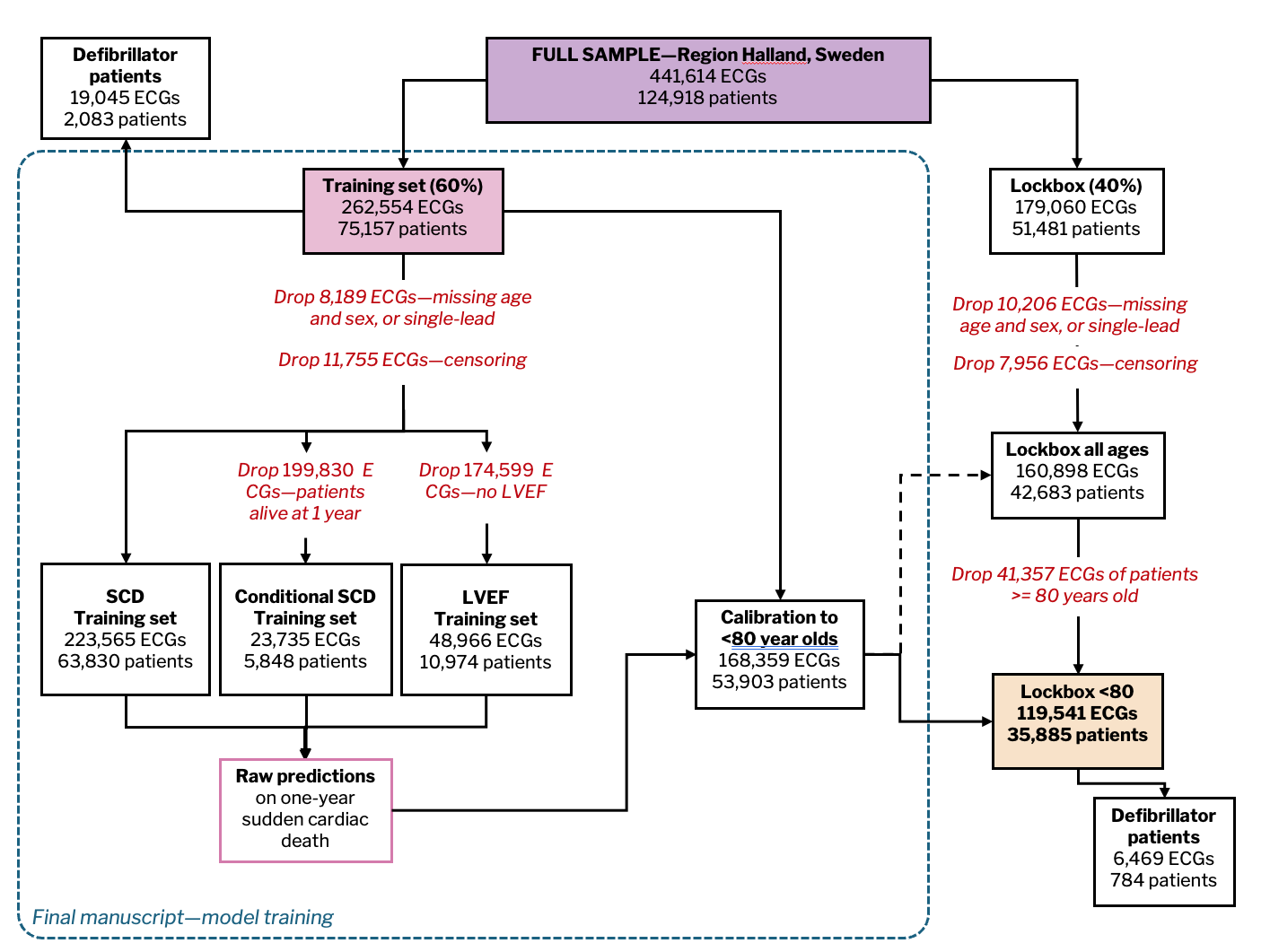


***Figure VII.A.2****: Data splits used for present (final) manuscript.*

The table below summarizes changes between the provisionally accepted version of the manuscript (30% training, 30% validation set) and the present version (60% training, 40% validation), highlighting figures central to the paper’s main claims (e.g., model AUC and PPV in all samples, mortality reductions in those with defibrillators, etc.). Overall, model performance improved, consistent with a larger training set size, providing additional reassurance regarding overfitting.

| **Provisionally accepted version** | **Current and final version** | **Summary of change** |
| --- | --- | --- |
| *Predictive performance: Sweden* | | |
| In a **30% hold-out set**, comprised of **89,414 ECGs from 29,108 Swedish patients** under 80 years old without defibrillators (we return to these groups below), … | After provisional acceptance, we retrained the model on all 60% of data we had accessed (262,554 ECGs from 75,157 patients), and without further modification, produced results in the remaining **40% “lock-box,”** shown below: **119,541 ECGs with one-year follow-up, from 35,885 patients under 80. The main predictive analyses rely on the subset of 113,072 ECGs from 35,417 patients without defibrillators** (to whom we return below) | Lock-box is **larger**, and fully **out of sample**. We clarified the exact sample size. |
| We calculate discriminative power using area under the receiver operating characteristic curve **(AUC):** **0.837** (patient-level bootstrapped 95% confidence interval [CI]: 0.810–0.862 | We calculate discriminative power using area under the receiver operating characteristic curve **(AUC):** **0.872** (patient-level bootstrapped 95% confidence interval [CI]: 0.843–0.899; | New model has **higher AUC** for SCD in Sweden |
| Our preferred threshold flags a high-risk group comprising **1.8%** of the sample, whose one-year sudden cardiac death rate is **6.1%** **(95% CI: 4.9-7.3%)**. | Our preferred threshold flags a high-risk group comprising **2.2%** of the sample, whose one-year sudden cardiac death rate is **7.0% (95% CI: 4.9-9.5%)**. | New model **high-risk group is larger** (risk ≥ RCT controls), and has **higher SCD rate** |
| Over and above the 6.1% of the high-risk group with sudden cardiac death, an **additional 3.5%** have VF/VT | Over and above the 7.0% of the high-risk group with sudden cardiac death, an **additional 3.8% per year (95% CI: 2.2-7.0)** have VF/VT. | New model high-risk group also has **higher VF/VT rate** |
| *Predictive performance: US and Taiwan* | | |
| With no fine-tuning or other modification of the model trained in Sweden, ‘zero-shot’ AUC for VF/VT is **0.792 (95% CI: 0.788-0.796)** in the US | With no fine-tuning or other modification of the model trained in Sweden, ‘zero-shot’ AUC for VF/VT is **0.822 (95% CI: 0.812-0.831)** in the US | New model has **higher AUC** for VF/VT in US |
| Using a similarly-sized high-risk group as in Sweden (top 1.8%), incidence of VF/VT in high-risk patients is **19.1% (95% CI: 18.0-20.3%**; vs. base rate of 3.8%). | Using a similarly-sized high-risk group as in Sweden (riskiest 2.2%, solid dark red point and arrow), incidence of VF/VT in high-risk patients is **29.1% (95% CI: 26.5-31.9%**; vs. base rate of 3.8%). | New model high-risk group has **higher VF/VT incidence** |
| using the same absolute risk threshold as in Sweden, … yields a large high-risk group—**22.6%** of the sample—that still has VF/VT incidence of **10.9% (95% CI: 10.7-11.3%)**. | using the same absolute risk threshold as in Sweden, … yields a larger high-risk group—**3.4%** of the sample (hollow dark red point and arrow)—with VF/VT incidence of **25.7% (95% CI: 23.6- 28.0%)**. | New model’s risk distribution in US changes (performance globally improved). Absolute threshold from Sweden yields **smaller high-risk group**, with **higher VF/VT** |
| Model predictions distinguish future arrhythmic arrests from controls with a zero-shot AUC of **0.769 (95% CI: 0.722-0.875)**. | Model predictions distinguish future arrhythmic arrests from controls with a zero-shot AUC of **0.767 (95% CI: 0.767 (95% CI: 0.706-0.823)**. | **Minimal change** in arrhythmic arrest AUC in Taiwan |
| Empirically, non-arrhythmic AUC is significantly worse (*p*<0.001) than the arrhythmic AUC, indeed it approaches random guessing: 0.5853 **(95% CI: 0.5446 - 0.6622)**. | Empirically, non-arrhythmic AUC is significantly worse (*p*<0.001) than the arrhythmic AUC, indeed it approaches random guessing: **0.582 (95% CI: 0.529 - 0.636)**. | **Minimal change** in placebo AUC in Taiwan |
| *Defibrillators and mortality high-risk patients* | | |
| We find that high-risk patients with a defibrillator in place are significantly less likely to die than predicted: Table 1, Column 1 shows the interaction term (defibrillator placement *x* high-risk) is large, negative, and significant. In absolute terms, those with defibrillators die **3.43 percentage points (p.p.)** less than the predicted rate of 6.39 p.p., a reduction of **53.7% (*p*<0.001)**. | We find that high-risk patients with a defibrillator in place are significantly less likely to die than predicted: Table 1, Column 1 shows the interaction term (defibrillator placement *x* high-risk) is large, negative, and significant. In absolute terms, those with defibrillators die **3.62 percentage points (p.p.)** less than the predicted rate of 6.65 p.p., a reduction of **54.4% (*p*<0.001)**. | High-risk ECG patients have **similar reductions in sudden cardiac death**, both absolute and relative |
| the ECG model’s high-risk group has a **13.7 p.p. (52.8%, *p*<0.001)** reduction in all-cause mortality relative to expected (25.9 p.p.) with defibrillators. | the ECG model’s high-risk group has a **12.6 p.p. (39.0%, *p*<0.001)** reduction in all-cause mortality relative to expected (32.4 p.p.) with defibrillators. | High-risk ECG patients have **similar absolute reductions in all-cause mortality**; higher base mortality in lock-box means **13.8% lower relative change** |
| *Comparison of ECG model to LVEF* | | |
| only **18.6%** of the high-risk ECG group were known to have reduced LVEF | only **13.9%** of the high-risk ECG group were known to have reduced LVEF. | ECG finds **even more unsuspected high-risk patients** without reduced LVEF |
| The model’s high-risk group overall has a higher sudden cardiac death rate than those with reduced LVEF: **6.1% vs. 4.4% (p=0.03)**. | The model’s high-risk group overall has a higher sudden cardiac death rate than those with reduced LVEF: **7.0% vs. 4.6% (p=0.02)**. | **Larger difference in SCD rate between ECG vs. LVEF** high-risk groups |

***Table VII.A.1.*** *Key claims from main text, comparison between provisionally accepted version and present final version.*

There are two places where some nuance did change, that we flag for completeness (blue text in the table); both are subtle, and neither required any rewriting or changes to the paper’s claims.

First, doubling the training set size allowed us to streamline the model post-processing: we replaced the cumbersome ensembling step (which required use of the validation set) with a simpler calibration in the under-80 year olds (in the training set only). We had prespecified our intent to make this change in the previous version and describe the change in detail in Supplement (IX: Final model creation). This step improved performance; it also yielded a different predicted risk distribution in the US, so when we applied the Sweden absolute risk threshold to define a US high-risk group, that group became smaller (3.4% vs 22.6% of the sample) and much higher-risk (25.7% vs 10.9%). The high-risk group is still larger than the high-risk group defined by the relative threshold (2.2% of the sample), and has very high VF/VT incidence. The change in risk distribution helps us adjudicate between two possible explanations for differences in model performance between the US and Sweden (both cited in the previous version): macro health differences between populations, and incentivized coding of VF/VT in the US. The new results suggest US patients’ risk of VF/VT conditional on the new model-predicted risk is now higher than in Sweden (while in the previous version conditional risks were more similar), thus giving more weight to incentivized coding as the dominant explanation. This is discussed extensively in Supplement III.C (Additional analysis on VF/VT—Sweden and US).

Second, when measuring defibrillator related mortality differences in high-risk ECG patients, we get very similar, very significant (p<0.001), absolute reductions in both sudden cardiac death and all-cause mortality. However, because the base mortality rate in the lock-box is higher than in the validation set (32.4 p.p. vs 25.9 p.p.), the relative all-cause mortality reduction is 13.8 p.p. smaller (39.0% vs. 52.8%; the respective absolute reductions are 12.6 p.p. vs. 13.7 p.p). The reduction is still large and significant, and the effect on our primary SCD outcome is unchanged (absolute change: 3.62 vs. 3.43; relative 54.4% vs 53.7%).

Finally, as noted in the main text, all results from the generative model and morphing procedure are drawn from the initial data set (30% training, 30% validation) rather than the lock-box: the process was computationally intensive, and subsequent results involved multiple rounds of human review (i.e., waveforms, blinded interpretation of linked MRIs) that were difficult to perform again. We believe this is acceptable as the emphasis of these results is hypothesis generation, rather than predictive performance.

Naturally, since both the model and the sample changed, there are many other minor changes; a comprehensive table is presented in Table VII.A.2.

| **Paper statistic** | **Accepted version** 30% Validation | **Final version** 40% Lock-box | | | **Meaningful change?** | **Notes** |
| --- | --- | --- | --- | --- | --- | --- |
| *Abstract* |  |  | | |  |  |
| Model high-risk group size (%) | 1.8 | 2.2 | | | No | New model identifies a larger high-risk group |
| Model high-risk group rate of sudden cardiac death (%) | 6.1 | 7.0 | | | No | New model high-risk group has higher SCD rate |
| Reduced LVEF group size (%) | 2.1 | 1.9 | | | No | No meaningful change |
| Reduced LVEF rate of sudden cardiac death (%) | 4.4 | 4.6 | | | No | " |
| Percent of model high-risk without reduced LVEF | 81.4 | 86.0 | | | No | " |
| Risk reduction - high-risk ECG patients + defibrillators (%) | 52.8 | 54.4 | | | No | " |
| *Predictive performance: Independent hold-out set, Sweden* | | |  | |  |  |
| Sample size - ECGs | 89,414 | | | 113,072 | No | 40% Lock-box vs 30% Validation set in original submission |
| Sample size - patients | 29,108 | | | 35,417 | No | " |
| AUC - ECG model | 0.837 (0.810-0.862) | | | 0.872 (0.843-0.899) | No | New model AUC is higher |
| AUC - AHA/ACC model | 0.738 | | | 0.697 | No | No meaningful change |
| AUC - Stanford ECG risk model | 0.641 | | | 0.655 | No | " |
| Rightmost point in F1 - bin size (%) | 0.05 | | | 0.2 | No | We improved graph aesthetics to fit figure guidelines, and added individual high-risk groups; in the process we increased bin size. The old top 0.05% bin contained only 45 patients, so (while it made the y-axis range look impressive) was not a clinically useful number. |
| Rightmost point in F1 - PPV (%) | 17.8 (6.6-28.9) | | | 11.0 (4.4-17.0) | No | Follows mechanically from larger bin size (lowers PPV and raises sensitivity) |
| Model high-risk group size (%) | 1.8 | | | 2.2 | No | New model identifies a larger group with risk ≥ RCT control groups |
| Model high-risk group rate of sudden cardiac death (%) | 6.1 (4.9-7.3) | | | 7.0 (5.0-9.4) | No | New high-risk group has higher SCD rate. New CI is slightly wider - old CI was erroneously not bootstrapped; this was fixed. |
| Model high-risk group rate of VF/VT (%) | 3.5 | | | 3.8 (2.2-7.0) | No | No meaningful change; added CI in new version |
| *External validation - US* |  | | |  |  |  |
| AUC (zero-shot) for VF/VT - US | 0.792 (0.788-0.796) | | | 0.822 (0.812-0.831) | No | New model has slightly higher AUC for VF/VT in US… |
| AUC (zero-shot) for VF/VT - Sweden | 0.742 (0.698-0.781) | | | 0.717 (0.676-0.756) | No | …and slightly lower AUC for VF/VT in Sweden. (Note that new model high-risk groups in both Sweden and US have higher VF/VT rate) |
| VF/VT incidence in high-risk group (same relative threshold as Sweden: top 2.2%) - US (%) | 19.1 (18.0-20.3) | | | 29.1 (26.5-31.9) | No | High-risk group has higher VF/VT incidence in US |
| VF/VT incidence in high-risk group (same absolute threshold as Sweden) - US (%) | 10.9 (10.7-11.3) | | | 25.7 (23.6-28.0) | No | " |
| Model high-risk group size (%) using same absolute risk threshold as Sweden - US (%) | 22.6 | | | 3.4 | Yes | Prespecified change to model post-processing (ensembling step replaced with a simpler calibration to under-80 year olds) improved performance, and yielded different predicted risk distribution in the US. Sweden absolute risk threshold thus produced smaller US high-risk group, with much higher risk of VF/VT (see row above). |
| Mean of predicted risk distribution - US vs. Sweden (%) | 2.9 vs. 0.5 | | | 0.64 vs. 0.38 | No | US mean predicted risk is still substantially higher than Sweden, but less so |
| SD of predicted risk distribution - US vs. Sweden (%) | 4.1 vs. 0.9 | | | 1.85 vs. 1.23 | No | US SD predicted risk is still substantially higher than Sweden, but less so |
| Coefficient from regression of VF/VT diagnosis on model-predicted risk - US vs. Sweden | - | | | 2.79 vs. 0.45 | Yes | Prespecified change to model and risk distribution changed US risk distribution such that, conditional on model-predicted risk, patients in the US are more likely to be coded as having VF/VT vs. Sweden. Old results suggested this difference was small. New results more in line with prior literature on known differences in coding incentives. |
| *External validation - Taiwan* |  | | |  |  |  |
| Median days from last ECG to arrest | 91 | | | 391 | No | Typo fixed in old version |
| AUC - primary cardiac arrests vs. controls | 0.769 (0.722-0.875) | | | 0.767 (0.706-0.823) | No | No meaningful change |
| AUC - non-primary cardiac arrests vs. controls | 0.585 (0.545 - 0.662) | | | 0.582 ( 0.529 - 0.636) | No | " |
| *Potential benefit of defibrillators for high-risk patients* | | |  | |  |  |
| ECG model high risk group |  | | |  |  |  |
| Sudden cardiac death - absolute risk reduction from defibrillators (p.p.) | 3.43 | | | 3.62 | No | No meaningful change |
| Sudden cardiac death - predicted asolute risk (p.p.) | 6.39 | | | 6.65 | No | " |
| Sudden cardiac death - relative risk reduction from defibrillators | 53.7 | | | 54.4 | No | " |
| All-cause mortality - absolute risk reduction from defibrillators (p.p.) | 13.7 | | | 12.6 | No | Reductions are the same in absolute terms… |
| All-cause mortality - predicted asolute risk (p.p.) | 25.9 | | | 32.4 | No | …but predicted baseline mortality is higher… |
| All-cause mortality - relative risk reduction from defibrillators | 52.8 | | | 39.0 | No | …resulting in slightly smaller but still very significant relative risk reductions |
| Reduced LVEF high risk group |  | | |  |  |  |
| Sudden cardiac death - absolute risk reduction from defibrillators (p.p.) | 1.86 | | | 2.99 | No | Reductions are larger in absolute terms… |
| Sudden cardiac death - predicted asolute risk (p.p.) | 4.5 | | | 4.43 | No | …with similar predicted baseline SCD risk... |
| Sudden cardiac death - relative risk reduction from defibrillators | 41.3 | | | 67.5 | No | …and remain in same relative neighborhood as RCT effect sizes (50-88%) |
| All-cause mortality - absolute risk reduction from defibrillators (p.p.) | 2.5 | | | 0.5 | No | Those with reduced LVEF and defibrillators no longer have significant reductions in all-cause mortality. This also matches the RCT literature on defibrillators. |
| All-cause mortality - predicted asolute risk (p.p.) | 11.1 | | | 11.5 | No | " |
| All-cause mortality - relative risk reduction from defibrillators | 22.5 | | | 4.6 | No | " |
| *Comparison of ECG risk prediction to state-of-the-art biomarker: LVEF* | | | | |  |  |
| Percent of sample with LVEF measured | 21.9 | | | 22.5 | No | No meaningful change |
| Percent of ECG high-risk group known to have reduced LVEF | 18.6 | | | 13.9 | No | Even fewer high-risk patients known to have reduced LVEF |
| Percent identified as high-risk by either model | 3.6 | | | 3.8 | No | No meaningful change |
| Percent agreement between ECG and LVEF in this group | 9.1 | | | 8.2 | No | " |
| Sudden cardiac death rate - ECG vs LVEF high-risk groups | 6.1% vs. 4.4%  (p=0.03) | | | 7.0% vs. 4.6%  (p=0.02) | No | Larger difference in SCD rate between ECG vs LVEF high-risk groups |
| Sudden cardiac death rate - ECG and LVEF agree on high-risk | 11.6 | | | 10.7 | No | No meaningful change |
| Sudden cardiac death rate - ECG low-risk but LVEF high-risk | 3.1 | | | 3.4 | No | " |
| Sudden cardiac death rate - ECG high-risk but LVEF low-risk or unknown | 4.8 | | | 6.4 | No | " |
| Sudden cardiac death rate - ECG high-risk but LVEF normal (low-risk) | 4.6 | | | 6.4 | No | " |
| *Methods* |  | | |  |  |  |
| Censoring of observations - training set | 6,090 of 117,039 (5.2%) | | | 12,969 of 247,286 (5.2%) | No | No meaningful change |
| Censoring of observations - hold-out set | 4,579 of 94,736 (4.8%) | | | 6,446 of 125,987 (5.1%) | No | " |

***Table VII.A.2.*** *All results from main text, comparison between provisionally accepted version and present final version.*

##### VII.B ICD 10 Codes used to identify Sudden Cardiac Arrest and Key Study Variables—Sweden

We replicated the standard epidemiological definition for sudden cardiac death based on death certificates from the literature, e.g., as in Chugh et al. (2004).^24^ We use the ICD-10 codes for cardiac causes of death described in Chugh et al., 2004: diseases of the heart (I00 to I09, I11, I20 to I51); congenital heart disease (Q20 to Q24); and ill-defined cause of death (R95 to R99). There are a variety of methods for defining “sudden” death based on death certificates. Studies that only have access to death certificates and not full EHR data typically use out of hospital or emergency department location of death. However, because all death certificates in our sample can be linked to a single source of truth for hospitalizations, we can also include those who died in the first day of hospitalization.

| **ICD-10 Codes to encode key medical risk factors** | |
| --- | --- |
| *Condition* | *ICD-10 Codes* |
| Sudden Cardiac Death | I00 to I09, I11, I20-I51, Q20-Q24, R95-R99  AND death outside of the hospital or in first 24 hours of admission |
| Acute Myocardial Infarction | I2101, I2102, I2109, I2111, I2119, I2121, I2129, I213, I214, I219, I21A1, I21A9, I220, I221, I222, I228, I229 |
| Prior Myocardial Infarction | I2101, I2102, I2109, I2111, I2119, I2121, I2129, I213, I214, I219, I21A1, I21A9, I220, I221, I222, I228, I229, I230, I231, I232, I233, I234, I235, I236, I237, I238, I241 |
| Ventricular Tachycardia | I472 |
| Ventricular Fibrillation | I490 |
| defibrillator placement | DF014, FPG10, FPG20, FPG30, FPG33, FPG36, FPG40, FPG43, FPG50, FPG96, DF016 |
| CHF & Cardiomyopathy | A3681, A381, A3950, A3952, B2682, B3320, B3322, B3324, B5881, D8685, I012, I090, I255, I400, I401, I408, I409, I41, I420, I421, I422, I423, I424, I425, I426, I427, I428, I429, I43, I514, J1082, J1182, O903, I0981, I110, I130, I132, I501, I5020, I5021, I5022, I5023, I5030, I5031, I5032, I5033, I5040, I5041, I5042, I5043, I50810, I50811, I50812, I50813, I50814, I5082, I5083, I5084, I5089, I509, I97130, I97131, O29121, O29122, O29123, O29129, Z95811, Z95812 |
| CAD history | I200, I201, I208, I209, I240, I248, I249, I2510, I25110, I25111, I25118, I25119, I252, I255, I256, I25700, I25701, I25708, I25709, I25710, I25711, I25718, I25719, I25720, I25721, I25728, I25729, I25730, I25731, I25738, I25739, I25750, I25751, I25758, I25759, I25760, I25761, I25768, I25769, I25790, I25791, I25798, I25799, I25810, I25811, I25812, I2582, I2583, I2584, I2589, I259, Z951, Z955, Z9861 |
| Hypertension | I10, O10011, O10012, O10013, O10019, O1002, O1003, O10911, O10912, O10913, O10919, O1092, O1093, O161, O162, O163, O164, O165, O169, H35031, H35032, H35033, I110, I119, I120, I129, I130, I1310, I1311, I132, I150, I151, I152, I158, I159, I160, I161, I169, I674, I973, O10111, O10112, O10113, O10119, O1012, O1013, O10211, O10212, O10213, O10219, O1022, O1023, O10311, O10312, O10313, O10319, O1032, O1033, O10411, O10412, O10413, O10419, O1042, O1043, O111, O112, O113, O114, O115, O119, O131, O132, O133, O134, O135, O139 |
| Hyperlipidemia | E780, E7800, E7801, E781, E782, E783, E784, E7841, E7849, E785 |
| Diabetes & Diabetic Complications | E089, E099, E109, E119, E139, E891, O24011, O24012, O24013, O24019, O2402, O2403, O24111, O24112, O24113, O24119, O2412, O2413, O24311, O24312, O24313, O24319, O2432, O2433, O24410, O24414, O24415, O24419, O24420, O24424, O24425, O24429, O24430, O24434, O24435, O24439, O24811, O24812, O24813, O24819, O2482, O2483, O24911, O24912, O24913, O24919, O2492, O2493, O99810, O99814, O99815, E0800, E0801, E0810, E0811, E0821, E0822, E0829, E08311, E08319, E08321, E083211, E083212, E083213, E083219, E08329, E083291, E083292, E083293, E083299, E08331, E083311, E083312, E083313, E083319, E08339, E083391, E083392, E083393, E083399, E08341, E083411, E083412, E083413, E083419, E08349, E083491, E083492, E083493, E083499, E08351, E083511, E083512, E083513, E083519, E083521, E083522, E083523, E083529, E083531, E083532, E083533, E083539, E083541, E083542, E083543, E083549, E083551, E083552, E083553, E083559, E08359, E083591, E083592, E083593, E083599, E0836, E0837X1, E0837X2, E0837X3, E0837X9, E0839, E0840, E0841, E0842, E0843, E0844, E0849, E0851, E0852, E0859, E08610, E08618, E08620, E08621, E08622, E08628, E08630, E08638, E08641, E08649, E0865, E0869, E088, E0900, E0901, E0910, E0911, E0921, E0922, E0929, E09311, E09319, E09321, E093211, E093212, E093213, E093219, E09329, E093291, E093292, E093293, E093299, E09331, E093311, E093312, E093313, E093319, E09339, E093391, E093392, E093393, E093399, E09341, E093411, E093412, E093413, E093419, E09349, E093491, E093492, E093493, E093499, E09351, E093511, E093512, E093513, E093519, E093521, E093522, E093523, E093529, E093531, E093532, E093533, E093539, E093541, E093542, E093543, E093549, E093551, E093552, E093553, E093559, E09359, E093591, E093592, E093593, E093599, E0936, E0937X1, E0937X2, E0937X3, E0937X9, E0939, E0940, E0941, E0942, E0943, E0944, E0949, E0951, E0952, E0959, E09610, E09618, E09620, E09621, E09622, E09628, E09630, E09638, E09641, E09649, E0965, E0969, E098, E1010, E1011, E1021, E1022, E1029, E10311, E10319, E10321, E103211, E103212, E103213, E103219, E10329, E103291, E103292, E103293, E103299, E10331, E103311, E103312, E103313, E103319, E10339, E103391, E103392, E103393, E103399, E10341, E103411, E103412, E103413, E103419, E10349, E103491, E103492, E103493, E103499, E10351, E103511, E103512, E103513, E103519, E103521, E103522, E103523, E103529, E103531, E103532, E103533, E103539, E103541, E103542, E103543, E103549, E103551, E103552, E103553, E103559, E10359, E103591, E103592, E103593, E103599, E1036, E1037X1, E1037X2, E1037X3, E1037X9, E1039, E1040, E1041, E1042, E1043, E1044, E1049, E1051, E1052, E1059, E10610, E10618, E10620, E10621, E10622, E10628, E10630, E10638, E10641, E10649, E1065, E1069, E108, E1100, E1101, E1110, E1111, E1121, E1122, E1129, E11311, E11319, E11321, E113211, E113212, E113213, E113219, E11329, E113291, E113292, E113293, E113299, E11331, E113311, E113312, E113313, E113319, E11339, E113391, E113392, E113393, E113399, E11341, E113411, E113412, E113413, E113419, E11349, E113491, E113492, E113493, E113499, E11351, E113511, E113512, E113513, E113519, E113521, E113522, E113523, E113529, E113531, E113532, E113533, E113539, E113541, E113542, E113543, E113549, E113551, E113552, E113553, E113559, E11359, E113591, E113592, E113593, E113599, E1136, E1137X1, E1137X2, E1137X3, E1137X9, E1139, E1140, E1141, E1142, E1143, E1144, E1149, E1151, E1152, E1159, E11610, E11618, E11620, E11621, E11622, E11628, E11630, E11638, E11641, E11649, E1165, E1169, E118, E1300, E1301, E1310, E1311, E1321, E1322, E1329, E13311, E13319, E13321, E133211, E133212, E133213, E133219, E13329, E133291, E133292, E133293, E133299, E13331, E133311, E133312, E133313, E133319, E13339, E133391, E133392, E133393, E133399, E13341, E133411, E133412, E133413, E133419, E13349, E133491, E133492, E133493, E133499, E13351, E133511, E133512, E133513, E133519, E133521, E133522, E133523, E133529, E133531, E133532, E133533, E133539, E133541, E133542, E133543, E133549, E133551, E133552, E133553, E133559, E13359, E133591, E133592, E133593, E133599, E1336, E1337X1, E1337X2, E1337X3, E1337X9, E1339, E1340, E1341, E1342, E1343, E1344, E1349, E1351, E1352, E1359, E13610, E13618, E13620, E13621, E13622, E13628, E13630, E13638, E13641, E13649, E1365, E1369, E138, E139 |

***Table VII.B.1****: Specific codes for sudden cardiac death, as well as those for other variables used in the analysis.*

##### VII.C Dataset construction and summary statistics—US


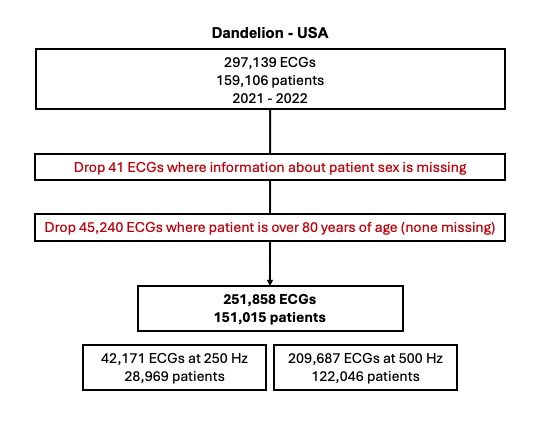


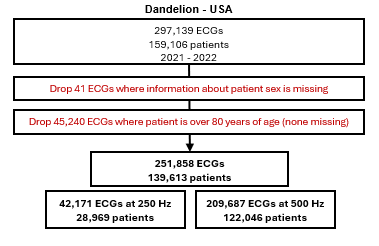


***Figure VII.C.1****: US data flow diagram. Note: the same patient can have both ECGs at 250 and 500 Hz, depending on where the ECG was acquired.*

|  | All | Patients with VF/VT | Patients without VF/VT |
| --- | --- | --- | --- |
| N patients | 139,613 | 2,211 | 137,984 |
| N ECGs | 251,858 | 9,664 | 242,194 |
| ECG count per patient | 1.80 | 4.37 | 1.76 |
| Demographics |  |  |  |
| Age | 54.55 (0.0324) | 61.81 (0.1296) | 54.26 (0.0331) |
| Female (%) | 0.5127 (0.0010) | 0.2829 (0.0046) | 0.5219 (0.0010) |
| Non-White (%) | 0.5218 (0.0010) | 0.4800 (0.0051) | 0.5234 (0.0010) |
| Rate of VF/VT, year after ECG | 0.0384 (0.0004) | 1 | 0 |
| Defibrillator implanted before ECG | 0.0350 (0.0004) | 0.2147 (0.0042) | 0.0278 (0.0003) |
| LVEF |  |  |  |
| LVEF recorded (%) | 0.3936 (0.0016) | 0.9000 (0.0032) | 0.3734 (0.0016) |
| LVEF≤35% | 0.0380 (0.0006) | 0.3107 (0.0050) | 0.0271 (0.0005) |
| Notes: Cell contents show mean (standard error). Table is at ECG (not patient) level. | | | |

***Table VII.C.1****: Summary statistics—US.*

##### VII.D Dataset construction and summary statistics—Taiwan


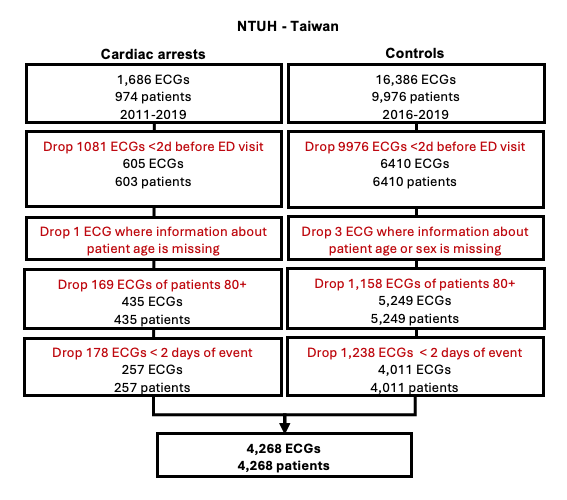


***Figure VII.D.1.*** *Taiwan registry data flow diagram.*

|  | *Registry patients: Cardiopulmonary arrest* | | *Controls* |
| --- | --- | --- | --- |
|  | (1) | (2) | (3) |
|  | Primary Cardiac Etiology | Non-Cardiac Etiology |  |
| *Panel A: Summary statistics* |  |  |  |
| N Patients | 235 | 368 | 6410 |
| N, age<80 | 183 | 252 | 5249 |
| N, age<80 and ECG more than 2 days before arrest | 96 | 161 | 4011 |
| *Demographics* |  |  |  |
| Age (mean) | 61.6 (0.808) | 64.0 (0.617) | 63.9 (0.136) |
| Female (fraction) | 0.354 (0.031) | 0.398 (0.026) | 0.459 (0.006) |
| VF/VT as presenting rhythm | 0.417 (0.032) | 0.043 (0.011) | - |

***Table VII.D.1.*** *Summary statistics—Taiwan registry data and controls.*

#### VIII Replication of main results—Sweden, all ages

##### VIII.A Summary statistics—Sweden (all ages)

|  | All | Sudden cardiac deaths | All others |
| --- | --- | --- | --- |
| Patients (n) | 45,669 | 621 | 45,361 |
| ECGs (n) | 168,854 | 2,762 | 166,092 |
| ECG Count per Patient (mean) | 3.70 | 4.45 | 3.66 |
| Demographics |  |  |  |
| Age (mean) | 65.638 (0.0474) | 83.399 (0.1946) | 65.343 (0.0478) |
| Female | 0.479 (0.0012) | 0.472 (0.0095) | 0.479 (0.0012) |
| Sudden cardiac death, year after ECG | 0.016 (0.0003) | 1 | 0 |
| VF/VT, year after ECG | 0.014 (0.0003) | 0.025 (0.003) | 0.013 (0.0003) |
| ICD Implanted before ECG | 0.067 (0.0006) | 0.106 (0.0059) | 0.067 (0.0006) |
| SCD risk factors |  |  |  |
| Prior VF/VT | 0.027 (0.0004) | 0.065 (0.0047) | 0.026 (0.0004) |
| LVEF recorded | 0.232 (0.001) | 0.301 (0.0087) | 0.231 (0.001) |
| LVEF≤35% | 0.028 (0.0004) | 0.11 (0.006) | 0.027 (0.0004) |
| CHF & Cardiomyopathy | 0.106 (0.0007) | 0.458 (0.0095) | 0.100 (0.0007) |
| Recent MI (40d before ECG) |  |  |  |
| Acute MI diagnosis | 0.037 (0.0005) | 0.087 (0.0054) | 0.036 (0.0005) |
| Positive troponin test | 0.179 (0.0009) | 0.568 (0.0094) | 0.173 (0.0009) |
| CAD history | 0.147 (0.0009) | 0.298 (0.0087) | 0.144 (0.0009) |
| Prior MI | 0.061 (0.0006) | 0.142 (0.0066) | 0.06 (0.0006) |
| Hypertension | 0.249 (0.0011) | 0.478 (0.0095) | 0.245 (0.0011) |
| Hyperlipidemia | 0.034 (0.0004) | 0.03 (0.0033) | 0.034 (0.0004) |
| Diabetes | 0.120 (0.0008) | 0.193 (0.0075) | 0.118 (0.0008) |
| MI: Myocardial infarction, or heart attack.  * High-sensitivity troponin thresholds are sex-specific: ≥34 ng/L male, ≥16 ng/L female | | | |

***Table VIII.A.1****: Summary statistics—Sweden validation set (all ages). Numbers are fractions (with standard errors clustered by patient in parentheses) unless noted. Statistics are calculated at ECG level.*

##### VIII.B Sudden cardiac death and VF/VT vs. model-predicted risk—Sweden (all ages)

***Figure VIII.B.1. Positive predictive value for sudden cardiac death, all ages.*** *Rate of sudden cardiac death (from death certificates) in high-risk group (y-axis, with bootstrapped 95% CI), vs. percentile threshold used to define high-risk group (x-axis); inset zooms in on the top 10%. Upper CI in the highest-risk group is censored by top of y-axis. Horizontal lines show median outcome rate in defibrillator trial control groups (lower line) and in patients with reduced LVEF (upper line).*

***Figure VIII.B.2. Ventricular arrhythmia incidence in high-risk group, vs. threshold for defining high-risk group, all ages****. Rate of VF/VT, combined with sudden cardiac death (SCD, from death certificates), in orange, and alone, in purple.*

##### VIII.C Mortality with vs. without defibrillator—Sweden (all ages)

**Observed differences in sudden cardiac death and all-cause mortality, by model predictions and defibrillator placement (all age groups)**. Columns 1-3 show results from regressions of sudden cardiac death on high-risk indicators (using same threshold as main analysis for ECG model, LVEF≤35% for LVEF), an indicator for defibrillator placement before ECG, and an interaction term. Columns 4-6 show regressions of all-cause mortality on the same variables. The interaction effect captures how much less high-risk patients with defibrillators die than expected. This analysis utilizes the absolute high-risk cutoff value established in the main text for individuals younger than 80 years.

|  | (1) | (2) | (3) | (4) | (5) | (6) |
| --- | --- | --- | --- | --- | --- | --- |
|  | **Sudden cardiac death** | | | **All-cause mortality** | | |
|  | *ECG model* | *LVEF* | *Both* | *ECG model* | *LVEF* | *Both* |
| *Risk indicator variables* | |  |  |  |  |  |
| ECG high-risk | 0.0932*** (0.002) |  | 0.0897*** (0.002) | 0.3268*** (0.004) |  | 0.3219*** (0.004) |
| LVEF high-risk |  | 0.0584*** (0.002) | 0.043*** (0.002) |  | 0.1152*** (0.005) | 0.0601*** (0.005) |
| Defibrillator present | -0.0041** (0.001) | 0.0005 (0.001) | -0.0034* (0.001) | -0.0553*** (0.003) | -0.0438*** (0.003) | -0.0584*** (0.003) |
| *Defibrillator x high-risk interactions* | |  |  |  |  |  |
| Defibrillator x ECG high-risk | -0.0362*** (0.003) |  | -0.0329*** (0.003) | -0.114*** (0.008) |  | -0.1146*** (0.008) |
| Defibrillator x LVEF high-risk |  | -0.0446*** (0.004) | -0.041*** (0.004) |  | -0.0311** (0.01) | -0.0187 (0.01) |
| Baseline Rate | 0.0083*** (0.0003) | 0.0105*** (0.0004) | 0.0076*** (0.0004) | 0.0675*** (0.0008) | 0.0767*** (0.0008) | 0.0665*** (0.0008) |
| *n*=160,898 for all regressions. All models control for patient age and sex. Baseline rate combines model intercept with age and sex effects (set to population means; SE accounts for covariances) to estimate outcome rate in low-risk patients without defibrillators. | | | | | | |

#### IX Deep learning model

Our model is structured as a 64-layer ResNet with 32 residual blocks. Each block contains two convolutional layers with 128 filters and a kernel size of 16. After each convolutional layer, batch normalization is applied, followed by dropout at a rate of 40%. The model’s input consists of 10-second 12-lead ECG waveforms sampled at 500 Hz. A schematic overview of this architecture is provided here.

**
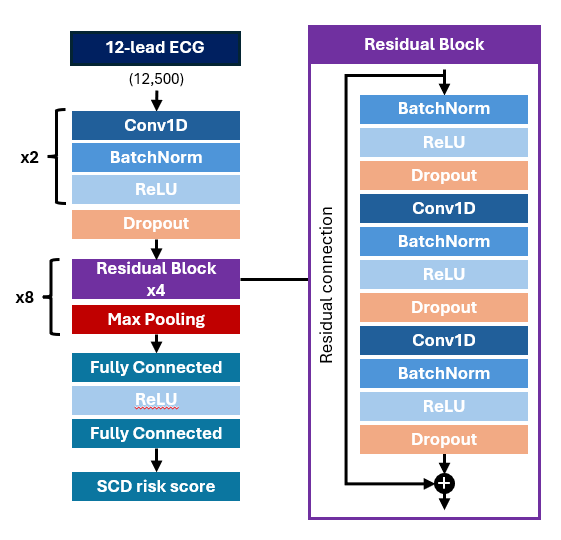
**

***Figure IX.1*** *Model architecture*

The model was trained as a multi-task predictor, designed to estimate the risk of sudden cardiac death and all-cause mortality at 3‑month, 6‑month, 1‑year, and 2‑year intervals. Observations from the final year of our dataset (2016) may have been censored (see Methods for details) and we use only uncensored observation-outcome pairs for training (e.g., an ECG performed in May 2016 will contribute to the training for 3- and 6-month outcomes, but not 1- and 2-year outcomes). The model also predicts whether a patient will experience acute myocardial infarction or have a positive troponin test, or receive an ICD code for ventricular tachycardia or ventricular fibrillation, within 1 year.

**Training Procedure**We trained the model using the Adam optimizer (learning rate 1×10^-5^), reducing the learning rate by half every 10 epochs. A minimum of 40 epochs was enforced, after which training was halted if the validation loss did not improve for 10 consecutive epochs; the model converged at 46 epochs under these conditions. The loss function used for optimization was binary cross-entropy with logits.

**Final model creation**

Before applying our model to the Swedish hold-out set or external datasets, we calibrate the model to the under-80 population where it will eventually be used, using only training data. Specifically, we use logistic regression (logit) to predict SCD from raw SCD-12-month predictions in the subset of training set ECGs taken in patients under 80 years old, effectively functioning as a linear final layer with sigmoid activation. This accurately calibrates predictions to the intended population, while still incorporating the learning from the large number of SCDs in the over-80 population. As noted in the accepted version of the manuscript, this replaces the procedure used in that version. (Previously, we ensembled all raw model outputs with age and sex in a logit, by splitting the hold-out in two, randomly selecting patients, and fitting the logit twice, once in each half; each logit was used to generate predictions for the other half, and these (mostly) out-of-sample predictions were used for evaluation.) The new procedure takes advantage of the larger training set size to improve and simplify the analytic pipeline, and means that no lock-box data were used for any part of the predictions. We emphasize that this structure was decided on before accessing the data lock-box, and pre-specified in the provisionally accepted version of the manuscript; no decisions or changes were made to the model or the calibration after accessing the lock-box.


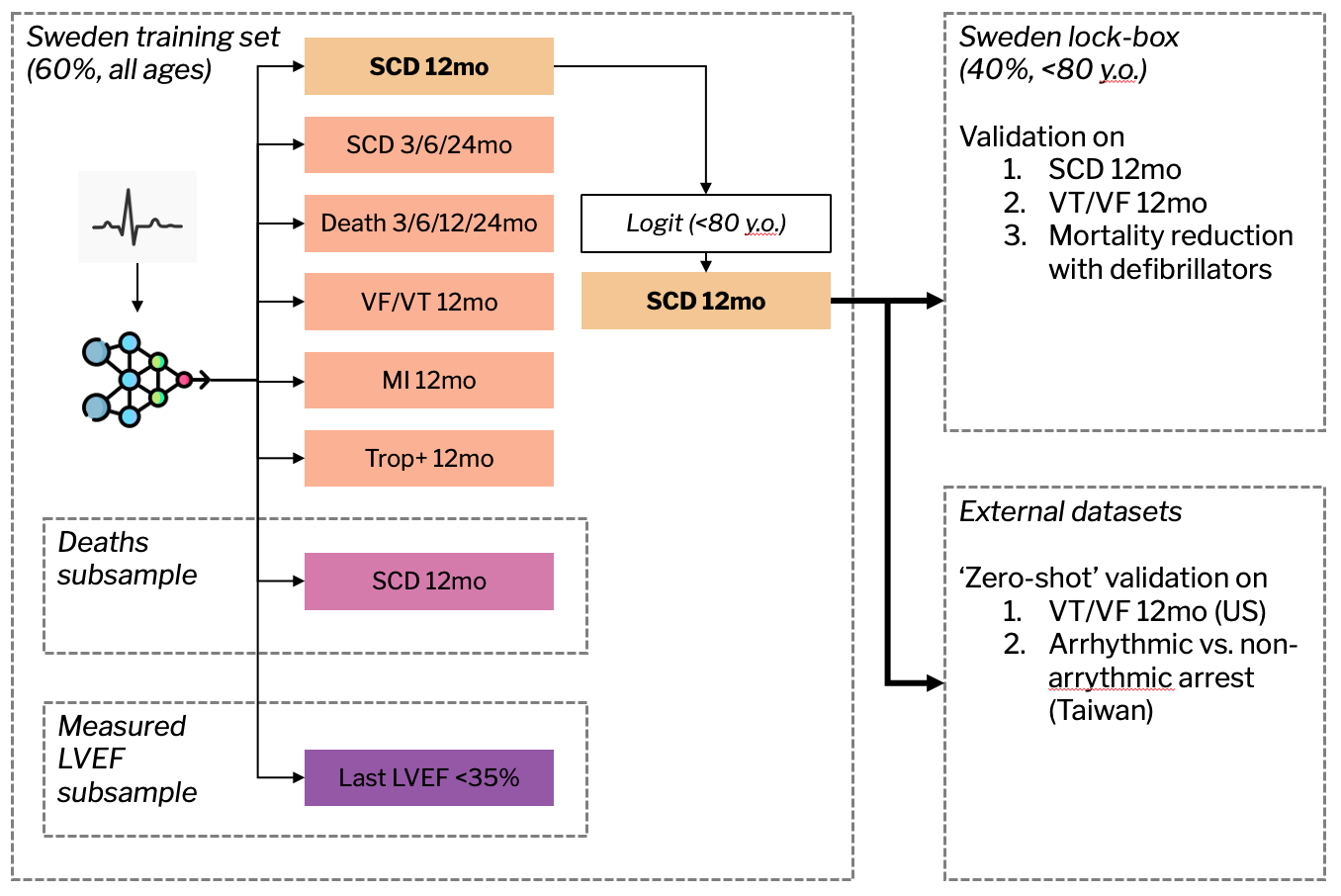


**Figure IX.2.** Model training and evaluation overview.

1. This is slightly different from the 16.0% of SCD with reduced LVEF in Table 1, because that number is produced in the entire validation set including those with defibrillators. We calculate model performance metrics on the non-defibrillator population to avoid improperly including the treatment effect of the defibrillator on outcomes. [↑](#footnote-ref-1)
2. On accessing the lock-box, we realized that all 4,024 single-lead ECGs (0.9% of all ECGs), which we had decided to exclude, had been placed in the lock-box instead, as noted in Figure VII.A.2; these were simply excluded from the lock-box as initially intended. [↑](#footnote-ref-2)
