# Supplementary material for: An ECG biomarker for sudden cardiac death discovered with deep learning
Source: Nature. 2026 Jun 24;655(8121):210–8. doi: 10.1038/s41586-026-10674-6 (PMC13323061; doi:10.1038/s41586-026-10674-6)
Supplement: Supplementary file 2 — Reporting Summary [file 41586_2026_10674_MOESM2_ESM.pdf]

nature portfolio

Corresponding author(s): Ziad Obermeyer

Last updated by author(s): Mar 4, 2025

Reporting Summary

Nature Portfolio wishes to improve the reproducibility of the work that we publish. This form provides structure for consistency and transparency in reporting. For further information on Nature Portfolio policies, see our Editorial Policies and the Editorial Policy Checklist.

Statistics

For all statistical analyses, confirm that the following items are present in the figure legend, table legend, main text, or Methods section.

n/a	Confirmed	
☐	☒	The exact sample size (*n*) for each experimental group/condition, given as a discrete number and unit of measurement
☐	☒	A statement on whether measurements were taken from distinct samples or whether the same sample was measured repeatedly
☐	☒	The statistical test(s) used AND whether they are one- or two-sided<br>*Only common tests should be described solely by name; describe more complex techniques in the Methods section.*
☐	☒	A description of all covariates tested
☒	☐	A description of any assumptions or corrections, such as tests of normality and adjustment for multiple comparisons
☐	☒	A full description of the statistical parameters including central tendency (e.g. means) or other basic estimates (e.g. regression coefficient) AND variation (e.g. standard deviation) or associated estimates of uncertainty (e.g. confidence intervals)
☐	☒	For null hypothesis testing, the test statistic (e.g. *F*, *t*, *r*) with confidence intervals, effect sizes, degrees of freedom and *P* value noted<br>*Give P values as exact values whenever suitable.*
☒	☐	For Bayesian analysis, information on the choice of priors and Markov chain Monte Carlo settings
☒	☐	For hierarchical and complex designs, identification of the appropriate level for tests and full reporting of outcomes
☒	☐	Estimates of effect sizes (e.g. Cohen's *d*, Pearson's *r*), indicating how they were calculated

Our web collection on statistics for biologists contains articles on many of the points above.

Software and code

Policy information about availability of computer code

Data collection	Scripts for data preprocessing and collection were written in python (version 3.8.8) using the open-source libraries pandas (version 1.5.3), numpy (version 1.24.4) and pyodbc (version 4.0.0)
Data analysis	The code for the data analysis and modeling was written in python (version 3.8.8). We leveraged the following open-source python libraries for our analysis: PyTorch (version 2.1.0, CUDA version 12.1), SciPy (version 1.11.3), Statsmodels (version 0.14.0), Jax (version 0.4.28), flax (version 0.8.3), Biosppy (version 1.0.0), scikit-learn (version 1.2.2), lifelines (version 0.27.8), captum (version 0.7.0), pandas (version 1.5.3), numpy (version 1.24.4), matplotlib (version 3.7.1), matplotlib_venn (version 1.1.1) and tqdm (version 4.66.1)

For manuscripts utilizing custom algorithms or software that are central to the research but not yet described in published literature, software must be made available to editors and reviewers. We strongly encourage code deposition in a community repository (e.g. GitHub). See the Nature Portfolio guidelines for submitting code & software for further information.

Data

Policy information about availability of data

All manuscripts must include a data availability statement. This statement should provide the following information, where applicable:

- Accession codes, unique identifiers, or web links for publicly available datasets
- A description of any restrictions on data availability
- For clinical datasets or third party data, please ensure that the statement adheres to our policy

The main dataset used in this study is derived from Region Halland (Sweden), a public regional health system. Owing to GDPR and patient data privacy regulations, this dataset cannot be shared publicly. The external validation dataset for the US patient cohort is drawn from the electronic health records of Sharp HealthCare (San Diego, CA) and is maintained by Dandelion Health. Under the terms of the data use agreement with Dandelion Health, these data are not publicly available. The external validation dataset for the Taiwanese patient cohort is derived from the hospital-based cardiopulmonary arrest registry at National Taiwan University Hospital (Taipei). This dataset is open-access and available via the Nightingale Open Science platform at https://docs.ngsci.org/datasets/arrest-ntuh-ecg/.

Research involving human participants, their data, or biological material

Policy information about studies with human participants or human data. See also policy information about sex, gender (identity/presentation), and sexual orientation and race, ethnicity and racism.

Reporting on sex and gender	We studied adult patients of both biological sexes, as recorded in each health system's electronic health records. These data did not include separate information on gender identity. Sex was incorporated into our predictive model and we report sex distributions publicly in table 2 as well as section VI and VII of the supplementary material, for all datasets.
Reporting on race, ethnicity, or other socially relevant groupings	n/a
Population characteristics	See table 1 in the paper as well as section VI and VII of the supplementary material.
Recruitment	No patients were recruited for this retrospective study.
Ethics oversight	This research was approved by the Ethical Review Board of Lund University (protocol 2016/517 and amendment 2024-02316-02).

Note that full information on the approval of the study protocol must also be provided in the manuscript.

Field-specific reporting

Please select the one below that is the best fit for your research. If you are not sure, read the appropriate sections before making your selection.

☒ Life sciences ☐ Behavioural & social sciences ☐ Ecological, evolutionary & environmental sciences

For a reference copy of the document with all sections, see nature.com/documents/nr-reporting-summary-flat.pdf

Life sciences study design

All studies must disclose on these points even when the disclosure is negative.

Sample size	A total of 441,614 electrocardiograms were collected in Region Halland (Sweden) between 2010–2016, performed on 187,677 unique patients. No a priori sample size calculations were performed, as we sought to maximize the available training data. Given that several ECG-based AI studied have been conducted using smaller training dataset sizes, we were confident that the available dataset size would be sufficient to train a capable model.<br>For external validation, we used 251,858 ECGs from 151,015 unique patients in the United States cohort, and 4,107 ECGs (including 96 future arrest cases plus 4,011 controls) in the Taiwanese registry.
Data exclusions	For all datasets we excluded samples where the corresponding patient had missing age or sex. In the main results we further exclude patients with age above 80 years as the benefits of ICD implantation are more limited in this group, but report results for the full age cohort in supplement VII.<br>In the Taiwanese external validation cohort we additionally exclude ECGs done in the 2 days leading up to the arrest, potentially in the context of the same acute event that precipitated arrest, which are less useful for the purposes of longer-term risk prediction and prevention. (<8% of the sample)
Replication	The code to replicate the algorithms developed in this study has been made publicly available and is accessible via: https://github.com/alexmschubert/ECG-SCD.
Randomization	Randomization was used to generate the train, validation and test sets for the Region Halland dataset that was used to develop our predictive and generative model. We evaluate on the complete external US-based and Taiwanese datasets.

Blinding	In a secondary analysis involving cardiac MRI for a subset of patients, the cardiologist who read the scans was blinded to all model-generated risk predictions during image review.

Reporting for specific materials, systems and methods

We require information from authors about some types of materials, experimental systems and methods used in many studies. Here, indicate whether each material, system or method listed is relevant to your study. If you are not sure if a list item applies to your research, read the appropriate section before selecting a response.

Materials & experimental systems

n/a	Involved in the study
☒	☐ Antibodies
☒	☐ Eukaryotic cell lines
☒	☐ Palaeontology and archaeology
☒	☐ Animals and other organisms
☒	☐ Clinical data
☒	☐ Dual use research of concern
☒	☐ Plants

Methods

n/a	Involved in the study
☒	☐ ChIP-seq
☒	☐ Flow cytometry
☒	☐ MRI-based neuroimaging

Plants

Seed stocks	n/a
Novel plant genotypes	n/a
Authentication	n/a
