# Supplementary material for: An ECG biomarker for sudden cardiac death discovered with deep learning
Source: Nature. 2026 Jun 24;655(8121):210–8. doi: 10.1038/s41586-026-10674-6 (PMC13323061; doi:10.1038/s41586-026-10674-6)
Supplement: Supplementary file 3 — Peer Review File [file 41586_2026_10674_MOESM3_ESM.pdf]

nature portfolio

Peer Review File

An ECG biomarker for sudden cardiac death discovered via deep learning

Corresponding Author: Professor Ziad Obermeyer

This file contains all reviewer reports in order by version, followed by all author rebuttals in order by version.

Version 0:

Reviewer comments:

Referee #1

(Remarks to the Author)
Manuscript Title: An ECG biomarker for sudden cardiac death discovered via deep learning

1. Summary of the Key Results
This manuscript presents a deep learning model trained on 441,614 ECGs from a Swedish regional health system to predict one-year sudden cardiac death (SCD). The model, a 64-layer ResNet, was trained using death certificates and further enhanced through an ensemble approach that incorporates prediction of LVEF and competing risk mortality. A high-risk group comprising 1.8% of the cohort had an annual SCD risk of 6.1%, comparable to or exceeding risk seen in landmark ICD trials. The model's performance was validated in U.S. and Taiwanese datasets (AUCs of 0.792 and 0.769, respectively) without fine-tuning. A generative model was used to reveal ECG features associated with high SCD risk, including a novel morphology in lead aVL. Importantly, ECG-defined high-risk patients demonstrated potential benefit from ICDs, with an estimated 60.3% reduction in all-cause mortality.

2. Originality and Significance
The study represents a novel and impactful contribution to cardiac risk stratification. Prior work has shown AI-based ECG predictors for arrhythmia or mortality risk, but this study is among the first to:
• Focus on SCD in a general population cohort.
• Use death certificate-based outcomes at scale.
• Apply an ensemble model for multi-faceted learning.
• Incorporate generative modeling for biomarker visualization.
The model uncovers risk not captured by traditional LVEF, with major implications for expanding ICD eligibility criteria and reducing missed opportunities for prevention. The hypothesis-generating nature of the morphing and interpretation tools also provides new scientific leads.The use of external validation sets across continents also strengthens its generalizability, although concerns regarding population heterogeneity remain (see below).

3. Data & Methodology
Validity of Approach:
The methodology is robust, with careful design of training, validation, and hold-out cohorts. Exclusion of ECGs post-ICD placement ensures clean outcome modeling. The ensemble approach (SCD prediction, cause of death comparison, and LVEF regression) leverages multi-task learning effectively.
Quality of Data:
The Swedish dataset is comprehensive and population-based, with rigorous linkage to health and mortality records. The U.S. and Taiwanese datasets provide geographic and healthcare diversity but differ structurally from the training cohort.
Presentation Quality:
The manuscript is well-structured with clear exposition. The visualizations (e.g., Figures 1–5) are compelling and well-integrated. Some methodological clarifications (e.g., calibration strategy, data imputation) could be expanded in the Supplemental materials.

4. Appropriate Use of Statistics and Treatment of Uncertainties
AUC is appropriately used for model discrimination, and 95% confidence intervals are consistently reported. However:
• Precision-recall curves and F1 scores should be added due to likely class imbalance (especially in external validations).

• Calibration assessment (e.g., Brier score or calibration plots) is missing and would be important for clinical application.
• The use of OLS to estimate mortality reduction is well justified, but causal inference should be more explicitly caveated, especially in observational settings with potential unmeasured confounding.

5. Conclusions: Robustness, Validity, Reliability
The conclusions are generally well-supported. Internal validation is strong, and the model's identification of a novel high-risk group not captured by LVEF is compelling. However, external generalizability is limited by differing cohort designs and outcome definitions:
The U.S. dataset uses VT/VF as a surrogate, not actual SCD.
The Taiwanese cohort is case-control-based and limited in scope.
Thus, while the model shows promising transportability, caution is warranted in interpreting performance across dissimilar populations.

6. Suggested Improvements
Major:
• Obermeyer et al. must be commended on a meticulous study design and attention to detail in developing a practically useful model that may have several applications in clinical care. That being said, there is an attempt to present a large amount of information in one paper that can be fine-tuned to divert the readers' attention to some key focus areas. Some weaker aspects of the paper include the attempts at validation with cohorts that do not represent the training sample. The validation of the Swedish training set model (SCD defined with death certificated) against the US population (SCD, VT/VF identified by medical records linkage) may represent a fundamentally different population. More striking is the validation in the Taiwanese cohort which represented patients visiting the emergency room which is not at all representative of the public health sample of the Swedish training sample. Given that there are some very insightful observations made by the authors, some of these "forced" validation cohort analysis may be placed in the supplement or better eliminated altogether, with the focus of the paper being the development of an ECG model to detect SCD, and the generative model to understand potential high-risk ECG features.
• Line 19-21: How do the authors reconcile the higher ECG model risk for SCD among patients with unkown LVEF when the survival was better than the low LVEF groups. Could this represent a higher risk patient for future decompensation and perhaps the follow-up duration was not enough to see the difference? Were there any attempts to look at other comorbidities for outcomes such as heart failure, or MI in the high versus low-risk ECG model?
• Line 183: In the external validation with the US dataset, the model performance was suboptimal in both the US and Swedish datasets, with the model actually performing better in the US dataset. The authors attempt to explain this by citing differences in the incidence of VT/VF in the US population versus Sweden. While this explanation makes sense to some extent, it does not address the bigger issue of underfitting of the model. What do the authors feel could be the reasons for this?
• To address the imbalances in the datasets, it would be ideal for the authors to report precision-recall curves, and F1 scores.
• Explore subgroup calibration and risk prediction performance across clinically relevant strata.
• The correlation of AI model output performance to specific signal feature changes has been elusive, and the report of the change in aVL impacting SCD risk is striking and almost startling. Generative AI models may find different signal features associated with an outcome or phenotype based on the initial "seed" ECG fed into the model. It is a bit surprising that changes in a single lead (aVL) were so strongly associated with the model's overall performance. Was this generative experiment repeated and was the finding in aVL consistent, or were changes in other leads more prominent in some experiments? Using lead aVL alone, what is the performance of the model – how much of the total impact of the model comes from this lead alone, and how well do other leads perform in isolation?

Minor:
• Include calibration curves or Brier scores to demonstrate how well predicted probabilities reflect actual risk.
• Report follow-up duration and censoring strategy in greater detail.
• Grammatical/spelling errors: Line 503

7. References: Appropriate Credit to Previous Work?
Yes, the manuscript references a comprehensive array of literature, including foundational ICD trials, LVEF limitations, and recent AI-ECG applications.

8. Clarity and Context
Abstract:
Accurate, informative, and well-written. It effectively communicates the problem, methods, key findings, and implications.

Introduction:
Thorough and contextual. It clearly outlines the limitations of current biomarkers and motivates the development of an ECG-based deep learning model.

Conclusion:
The discussion of scientific and clinical implications is balanced and insightful. The hypothesis about myocardial fibrosis and conduction abnormalities is intriguing and biologically plausible, though it should be clearly labeled as speculative
Recommendation:
Major Revisions
The manuscript is a high-quality and innovative contribution with substantial clinical and scientific value. Revisions to

improve clarity on external validation limitations, expand evaluation metrics, and fine-tune presentation of exploratory findings will enhance its impact and interpretability.

Referee #2

(Remarks to the Author)
This is a novel and exemplary study showcasing how we can derive new, human interpretable knowledge in medical science, specifically, about ECG and sudden cardiac death (SCD), from deep learning. The authors developed a deep learning model with a large dataset to predict SCD from ECGs, and validated the model in external datasets internationally from different cohorts at different locations. Through a series of interesting statistical analysis, the authors showed that: 1) the model can accurately predict both SCD and VF/VT, despite that it was trained on death certificates; 2) the model is specific to predict arrhythmic death; 3) the predicted high-risk group can benefit from ICDs, but the risk has not been fully captured by the widely used metric LVEF. Until here, the manuscript has told an excellent research story, like previous publications on this topic, but the following analysis has made this manuscript extraordinary. The authors exploited what exact patterns / biomarkers in ECGs are predictors learned by the model to predict SCD. By visualizing ECG waveform morphs, they discovered that a missing S-wave in lead aVL is a strong predictor for SCD.
My comments are below:
1. The number of patients is not mentioned anywhere in the main text. This is a particularly important factor people consider when evaluating the performance metrics and the validity of results in clinical studies. I think this should be added to the abstract and main text, instead of remaining in the methods and supplements.
2. The only performance metric reported in the manuscript is AUC. AUC is known to be sensitive to class imbalance (https://doi.org/10.1371/journal.pone.0118432), and SCD is indeed very rare in the population. Other metrics that are less sensitive to class imbalance should be reported as well in complementary to AUC, like F score, balanced accuracy, PR curves, etc.
3. The authors chose a threshold based on population percentile. I agree on the rationale how this was chosen, but how did you apply this threshold on other cohorts (US and Taiwan)? From figure 2b, it seems that you still chose the top 1.8% as high-risk groups. If a model needs information about the patient distribution to set threshold, then for new cohorts with no prior distribution information, how will you use it?
4. The analysis about VFVT on page 5 is interesting. The authors showed two (almost) monotonically increasing curves of VFVT in figure 2, and suggesting the model is predicting arrhythmic death. On the right side of figure 2a, the VFVT alone risk is not increasing so monotonically, but the VFVT plus SCD risk is rising sharply. Does it suggest the model picks more unpreventable non-arrhythmic death in the high-risk group? To complement this analysis, I would like a curve of the ratio between (VFVT+SCD) and VFVT. Assuming detected the ratio between recorded VFVT and all VFVT is a constant; the ratio between all VFVT and death certificates is also a constant, then we should see a flat curve of (VFVT+SCD)/VFVT.
5. Re “We can then compare this AUC to the AUC for arrhythmic arrests above: if our model is specific to arrhythmia, it should perform worse on the placebo test; if our model inappropriately flags both arrhythmic and non-arrhythmic deaths as high risk, the statistics should be similar. Empirically, the non-arrhythmic AUC is significantly worse ($p<0.001$) than the arrhythmic AUC, indeed it approaches random guessing: 0.5853 (0.5446 - 0.6622).”: Interesting analysis. I need confirm I understand correctly. This calculates two AUCs, one for arrhythmic, the other for non-arrhythmic. If so, what are the event rates for either of them? Following comment 2, I suggest reporting additional metrics, especially if the rates are different, because AUC is sensitive to event rates.
6. Re “This latter is the quantity of interest: it captures mortality differences in high-risk patients with defibrillators, relative to what we would expect based on (i) their high risk and (ii) the fact that they had a defibrillator, independently.”: Very clear idea and interesting analysis.
7. Re Table 1: Why ICD increases risk for SCD but decreases risk for all-cause mortality? This counter intuitive results make me double this methodology and other results in this table.
8. The missing S-wave morph in aVL, although has not been discussed much before, also correlates with left anterior–superior fascicle block and axis deviation. There are existing ECGs characteristics for this condition in different leads. How much additional information does the proposed biomarkers provide in predicting SCD?
9. I am disappointed that the model weights are not provided. I understand the original data cannot be made public, but I think the weights should be. The model is on ECG, and the model weights do not contain identifiable information.

(Remarks on code availability)
It is hard to reproduce if model weights are not published.

Referee #3

(Remarks to the Author)
I co-reviewed this manuscript with one of the reviewers who provided the listed reports.

Version 1:

Reviewer comments:

Referee #2

(Remarks to the Author)
All my previous comments have been thoroughly addressed and deeply discussed in both the authors' responses and the revised manuscript. I appreciate their extensive and insightful interpretation of the new results, particularly the detailed explanations regarding the VF/VT observations and the discrepancy between SCD and all-cause mortality. These additions meaningfully strengthen the manuscript. I congratulate the authors on this impactful work and its brilliant presentation.
I have no further comments.

(Remarks on code availability)
The repository provides a good starting point for understanding the methods used in the paper, but the documentation has weaknesses. The README is a decent overview but lacks crucial details on pipeline execution. The code is sparsely commented, with minimal docstrings. I recommend enhancing the README and adding comprehensive comments and docstrings to improve reproducibility and reusability.

Open Access This Peer Review File is licensed under a Creative Commons Attribution 4.0 International License, which permits use, sharing, adaptation, distribution and reproduction in any medium or format, as long as you give appropriate credit to the original author(s) and the source, provide a link to the Creative Commons license, and indicate if changes were made.
In cases where reviewers are anonymous, credit should be given to 'Anonymous Referee' and the source.
The images or other third party material in this Peer Review File are included in the article's Creative Commons license, unless indicated otherwise in a credit line to the material. If material is not included in the article's Creative Commons license and your intended use is not permitted by statutory regulation or exceeds the permitted use, you will need to obtain permission directly from the copyright holder.
To view a copy of this license, visit https://creativecommons.org/licenses/by/4.0/

Cover letter

Dear Editor:

Many thanks for giving us the opportunity to revise our manuscript. We appreciate the careful, thoughtful reviews, and think the revised version is considerably improved as a result. We summarize the main changes here, and include point by point responses below.

The reviewers were in agreement on the need to **expand the set of performance metrics we report**. We have added these for both clinically-relevant subgroups in our main Swedish sample (Supplement I.A), and external validation datasets (Supplement III.A), as requested: calibration, ROC, and precision-recall curves; F1 and Brier scores, expected calibration error, accuracy, and balanced accuracy. These results confirm good performance across subgroups and datasets, and complement the more clinically-oriented metrics in the main text. They also highlight some interesting new findings, which we discuss in line below.

The reviewers also probed aspects of **model generalization to external validation datasets**.

- R1 emphasized the very *different cohort designs and outcome definitions* in the external datasets vs. the Swedish dataset. We now prominently acknowledge the complexity this creates in terms of interpreting performance metrics, just as we introduce the datasets. We have added deeper discussion and new results on dataset-specific issues: incentivized VF/VT measurement and health inequality in the US , and the selected nature of cases and controls in patients seeking emergency care in Taiwan (Supplement III.A-C). Overall, we absolutely agree that these differences complicate interpretation. However, they also bring advantages: they allow us to validate predictions (i) in diverse global populations, which is rare in health machine learning, and (ii) across many different ways to measure sudden cardiac death - including the arrhythmias that cause it, and detailed case investigations common in the literature - which is important given the absence of a perfect measure. We feel these are important for the paper's longer-term impact, in clinical audiences especially. So while R1 suggested moving these results to the Supplement, we hope you agree that they are important enough to keep in the main text (we have tried to condense as much as possible).
- In response to R2's excellent question about *setting a high-risk threshold in new populations where we do not have distributional information*, we now apply the same absolute risk threshold from Sweden to define high-risk groups in the US and Taiwan. This requires no population information, and results remain good: the new threshold yields PPV and sensitivity (and most other metrics) at least as high as in Sweden.

The reviewers also suggested fascinating new ways to **deepen understanding of the ECG morphology** 'discovered' by the generative model.

- We have added an analysis that speaks to R1's question about the *predictive power of leads other than aVL*. Interestingly, predictive power appears broadly distributed across

leads - even while human-visible signal is concentrated in aVL - suggesting a diffuse process throughout the myocardium (Supplement V.H).
- In response to R2’s question about *how much the new morphology adds to the known feature of left axis deviation (LAD, a measure of left anterior fascicular block)*, we now break out the LAD coefficient separately, so its predictive power can be compared to the 1st- and 2nd-difference features (we had already adjusted for this in the original submission, but had not shown the coefficients). This shows that the new feature adds independent signal of about the same magnitude as LAD (Supplement V.G).

Finally, we have added a number of **clarifications requested by the reviewers** (on sample size, how we deal with censoring, interpretation of regression coefficients, etc.), detailed below. One important question raised by R2 concerns the decreasing share of VF/VT (relative to the sum of VF/VT and sudden cardiac deaths) in the high-risk group in Sweden, and whether this might represent fewer preventable deaths in this group. We show (via a simple simulation) that even small amounts of censoring in high-risk patients, i.e., failure to measure VF/VT due to sudden death outside of the hospital, can account for the pattern we see.

We thought it might be useful at this stage to flag our **plan for the data ‘lockbox,’** in case we are fortunate enough to have the opportunity to move forward with this manuscript at *Nature*. So far, we have accessed only 60% of our full Swedish dataset (which was split into a 30% training set, and a 30% validation set in which all results to date were produced); the remaining 40% are untouched. Our plan, should the manuscript move ahead in the review process, is to retrain the model on the 60% we have already accessed, in exactly the same way we trained our currently model, and immediately freeze it - i.e., no further modifications. We will then reproduce the main results in the 40% lockbox. This procedure ensures the integrity of the hold-out. Of course it also introduces some uncertainty - and we would completely understand if anything at that stage causes you to change course. However, we view this as unlikely: this was our plan from the start of this project, so we have been careful about overfitting. And the new training set and lockbox will be larger than the training and validation set in the current version (60% vs. 30%, and 40% vs. 30%, respectively). So we expect model performance to be at least as good, and confidence intervals to be at least as tight.

Thank you again for giving us this chance to improve the manuscript, and we look forward to hearing from you.

Ziad

Referee #1 (Remarks to the Author):

1. Summary of the Key Results
This manuscript presents a deep learning model trained on 441,614 ECGs from a Swedish regional health system to predict one-year sudden cardiac death (SCD). The model, a 64-layer ResNet, was trained using death certificates and further enhanced through an ensemble approach that incorporates prediction of LVEF and competing risk mortality. A high-risk group comprising 1.8% of the cohort had an annual SCD risk of 6.1%, comparable to or exceeding risk seen in landmark ICD trials. The model's performance was validated in U.S. and Taiwanese datasets (AUCs of 0.792 and 0.769, respectively) without fine-tuning. A generative model was used to reveal ECG features associated with high SCD risk, including a novel morphology in lead aVL. Importantly, ECG-defined high-risk patients demonstrated potential benefit from ICDs, with an estimated 60.3% reduction in all-cause mortality.

2. Originality and Significance
The study represents a novel and impactful contribution to cardiac risk stratification. Prior work has shown AI-based ECG predictors for arrhythmia or mortality risk, but this study is among the first to:
• Focus on SCD in a general population cohort.
• Use death certificate-based outcomes at scale.
• Apply an ensemble model for multi-faceted learning.
• Incorporate generative modeling for biomarker visualization.
The model uncovers risk not captured by traditional LVEF, with major implications for expanding ICD eligibility criteria and reducing missed opportunities for prevention. The hypothesis-generating nature of the morphing and interpretation tools also provides new scientific leads.The use of external validation sets across continents also strengthens its generalizability, although concerns regarding population heterogeneity remain (see below).

Thank you for the thoughtful summary - we return to discuss the population heterogeneity point in detail below. To summarize that discussion:

1. We have added the caveats you note to the main text, when we introduce the external validation datasets: we note immediately that their different designs and outcome definitions vs. the main Swedish dataset complicate interpretation of performance metrics. We have also gone deeper into the specific issues in each dataset, adding both more discussion and empirical results on differences in population characteristics and outcomes measurement in the US and Taiwan.
2. While acknowledging these weaknesses prominently, we also note that they present some advantages: in addition to geographic and ethnic diversity, the datasets' different outcome definitions - death certificates, detailed case reviews, and well-measured shockable rhythms - each speak to different measurable aspects of sudden cardiac

death. Based on feedback from clinicians, these contrasts paint a fuller picture of model performance across populations and outcomes, and increase the study's clinical validity.

3. Data & Methodology
Validity of Approach:
The methodology is robust, with careful design of training, validation, and hold-out cohorts. Exclusion of ECGs post-ICD placement ensures clean outcome modeling. The ensemble approach (SCD prediction, cause of death comparison, and LVEF regression) leverages multi-task learning effectively.
Quality of Data:
The Swedish dataset is comprehensive and population-based, with rigorous linkage to health and mortality records. The U.S. and Taiwanese datasets provide geographic and healthcare diversity but differ structurally from the training cohort.
Presentation Quality:
The manuscript is well-structured with clear exposition. The visualizations (e.g., Figures 1–5) are compelling and well-integrated. Some methodological clarifications (e.g., calibration strategy, data imputation) could be expanded in the Supplemental materials.

Thank you - we agree on the US and Taiwan datasets, and have added prominent caveats regarding their structural differences vs. Sweden. We return to discuss this, as well as the methodological clarifications, as they arise in more detail below.

4. Appropriate Use of Statistics and Treatment of Uncertainties
AUC is appropriately used for model discrimination, and 95% confidence intervals are consistently reported. However:
• Precision-recall curves and F1 scores should be added due to likely class imbalance (especially in external validations).
• Calibration assessment (e.g., Brier score or calibration plots) is missing and would be important for clinical application.

We have added the requested figures and metrics to the Supplement (III), where they complement the more clinically-oriented metrics presented in the main text.

Specifically, we now show calibration curves, as well as ROC and precision-recall curves, for both Sweden and the external validation samples (III.A). We also present all the requested metrics, as well as some additional ones, in a new table: AUC and AUPRC statistics, F1 and Brier scores, as well as accuracy and balanced accuracy (in response to another reviewer, who had similar feedback). Regarding calibration metrics, Brier scores can be hard to interpret for rare outcomes, so we also include expected calibration error as an alternative metric of calibration that is less sensitive to base rates (III.B). Note that, in response to another reviewer comment, we now use the absolute risk threshold from Sweden to define discrete high-risk

groups in the external validation datasets (rather than classifying the top 1.8% as high-risk as we did in the initial submission, which requires knowledge of the population distribution).

This was a very valuable exercise, and we are grateful to you for suggesting it - we summarize some of the new insights here (and have inserted these into the main text and Supplement where relevant).

Calibration. As shown in the calibration curves, the model is very well calibrated in the Swedish validation set for predicting sudden cardiac death. The overall Brier score is 0.005 (95% CI: 0.005, 0.006). To give some sense of a baseline against which to compare this number, we calculate the model's Brier score in the subset of those with LVEF recorded, 0.011 (95% 0.010, 0.013), which is very close to LVEF, 0.011 (95% CI: 0.010, 0.012; to get LVEF into a probability suitable for Brier calculation, we include it in a logistic regression mode with age and sex in the training set, and calculate Brier score in the validation set, just as we do for the ECG model). Results for additional clinical subgroups are shown in response to your comment below, and in Supplement I). We can also assess calibration in the Taiwanese dataset, which we report naively, treating the case-control sample as a 'population' (with a base rate of the outcome is 1.6%, vs. 0.5% in Sweden, driven by the ratio of cases to controls; this is arbitrary, but likely not far from realistic population rates). With these caveats in mind, calibration remains good in Taiwan: the risk increase is linear at 45-degrees, i.e. observed risk rises proportionally and linearly with predicted risk. The intercept is slightly shifted above the 45-degree line, but the 95% confidence intervals include it. Of note, the highest-risk decile in Taiwan is almost 2x as risky as the highest-risk decile in Sweden, but calibration is linear and unaffected.

Because the model was not trained to predict VF/VT, we should not necessarily expect perfect calibration on the 45-degree line for this outcome. However, given the physiological relationship between VF/VT and the prediction target of SCD, we can make some inferences regarding what the graph should look like. First, VF/VT and SCD should be strongly positively correlated: VF/VT causes sudden cardiac death. Reassuringly, we see the monotonic increases in VF/VT vs. predicted risk we expect as a result, i.e., higher risk bins have higher rates of VF/VT. Second, not all VF/VT leads to SCD (these rhythms can self-resolve), so SCD is a subset of VF/VT. Thus we should expect a calibration slope >1, i.e., above the 45-degree line, which is also what we observe. Again here, we emphasize that the highest-risk decile in the US is 4x as risky as the highest-risk decile in Sweden, but calibration remains linear.

Precision and recall. The precision-recall curve for sudden cardiac death shows clinically high precision (PPV) in the top few percent with reasonable sensitivity. At our preferred threshold (based on ICD RCTs, with median control-arm SCD rates of 4.9%), precision is 6.1% with a sensitivity of 20.4% (see table). As a point of comparison, reduced LVEF has a precision of 4.4% and a sensitivity of 18.1% in our sample.[1] The corresponding F1 scores are 0.093 (95%

[1] This is slightly different from the 21.7% of SCD with reduced LVEF in Table 1, because that number is produced in the entire validation set including those with defibrillators. We calculate model performance metrics on the non-defibrillator population to avoid improperly including the treatment effect of the defibrillator on outcomes

CI: 0.077, 0.108) for our model vs. 0.070 (95% CI: 0.058, 0.083) for LVEF. In Taiwan (with the same caveats regarding case-control design noted above), the precision-recall curve is at least as good as in Sweden, with very similar PPV, sensitivity, and F1 scores.

The difference between the Swedish vs. US precision-recall curves is quite striking - more so than with AUC, because here the base rate affects precision - with US precision and recall both far better than in Sweden. This relates to one of your subsequent questions, on the differences between the US and Sweden datasets, which we return to below where you raise that point (and have added a new section to the Supplement on this topic: III.C). But since it's relevant here, we'll summarize what we think drives better performance in the US: the higher risk, and also higher risk variance, of the US population, likely reflecting differences in general health and health equity. The simplest way to see this is by comparing the mean and SD of predicted risk: in the US these are 0.029 and 0.041, while in Sweden they are 0.005 and 0.009, respectively. The low average risk makes precision lower, and the compressed risk distribution in Sweden makes the model's discrimination task harder. Another factor that may contribute is that VF/VT are likely better measured in the US, due to strong financial incentives. This could improve model performance, particularly if high-risk patients are measured more in the US: if our model (trained on death certificates) accurately predicts VF/VT in a patient who does have VF/VT, it will often appear "wrong" in Sweden—because VF/VT happens but is not recorded. Conversely, it will do better in the US, where these events are more likely to be recorded.

Calibration curves (decile bins)

Outcome: Sudden cardiac death

Datasets: Sweden vs. Taiwan

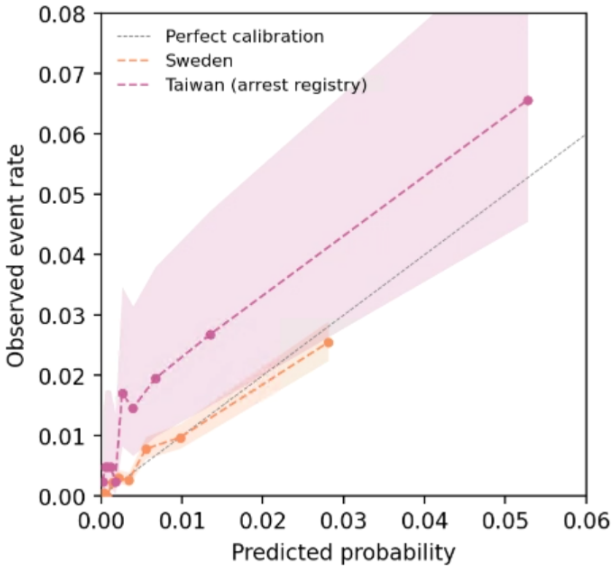

Outcome: VF/VT

Datasets: Sweden vs. US

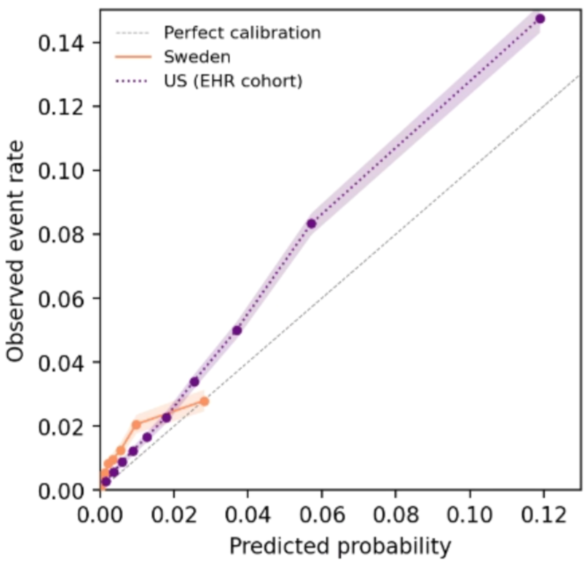

Receiver-operating-characteristic curves

Outcome: Sudden cardiac death
Datasets: Sweden vs. Taiwan

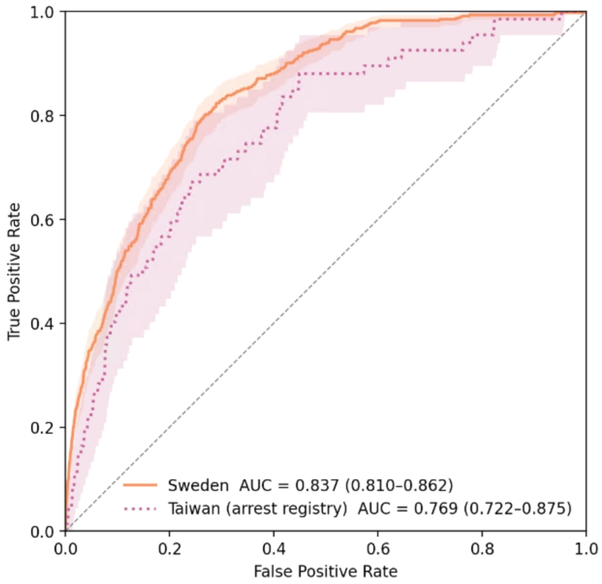

Outcome: VF/VT
Datasets: Sweden vs. US

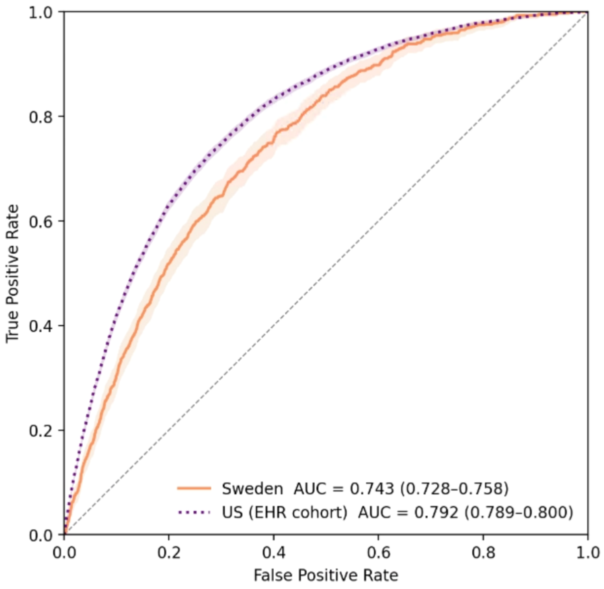

Precision-recall curves

Outcome: Sudden cardiac death
Datasets: Sweden vs. Taiwan

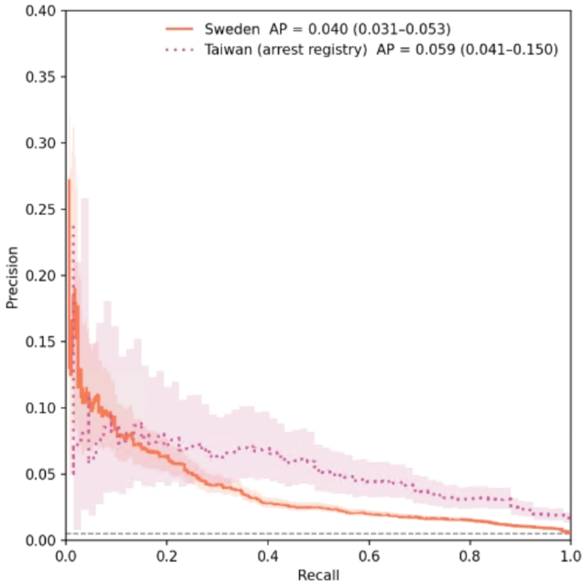

Outcome: VF/VT
Datasets: Sweden vs. US

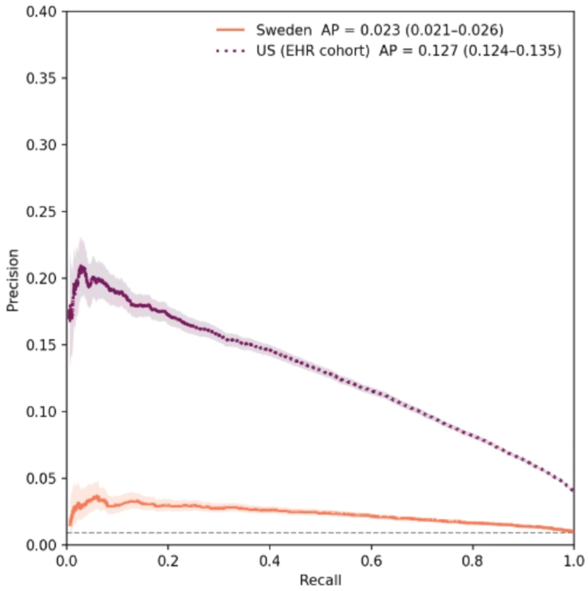

Additional performance metrics

Outcome	Sudden cardiac death		VF/VT	
Population	*Sweden*	*Taiwan*‡	*Sweden*	*US*
n	89,414	4107	89,414	251,858
Base rate of outcome	0.0052	0.0163	0.0094	0.0384
Calibration metrics				
Brier Score	0.005	0.016	0.009	0.036
	(0.005, 0.006)	(0.013, 0.023)	(0.009, 0.010)	(0.035, 0.037)
Expected Calibration Error (ECE)†	0.001	0.008	0.004	0.01
	(0.001, 0.001)	(0.006, 0.015)	(0.004, 0.005)	(0.009, 0.010)
Threshold-independent classification metrics				
AUC	0.837	0.769	0.743	0.792
	(0.810, 0.862)	(0.722, 0.875)	(0.728, 0.758)	(0.789, 0.800)
AUPRC	0.04	0.059	0.023	0.127
	(0.031, 0.053)	(0.041, 0.150)	(0.021, 0.026)	(0.124, 0.135)
Threshold-dependent classification metrics				
High risk fraction*	0.018	0.054	0.018	0.226
PPV	0.061	0.067	0.034	0.109
	(0.050, 0.070)	(0.042, 0.120)	(0.025, 0.043)	(0.107, 0.113)
Sensitivity	0.204	0.224	0.063	0.643
	(0.168, 0.239)	(0.145, 0.451)	(0.046, 0.080)	(0.635, 0.659)
F1 Score	0.093	0.103	0.044	0.186
	(0.077, 0.108)	(0.065, 0.177)	(0.033, 0.056)	(0.183, 0.192)
Accuracy	0.979	0.937	0.974	0.785
	(0.979, 0.980)	(0.931, 0.953)	(0.973, 0.975)	(0.783, 0.788)
Balanced Accuracy	0.594	0.586	0.523	0.717
	(0.576, 0.611)	(0.546, 0.699)	(0.515, 0.531)	(0.713, 0.725)

Note: 95% CIs are computed via 1,000 bootstrap resamples.

* High-risk threshold is set as described in the main text in the Swedish sample, based on defibrillator trial control group rates of sudden cardiac death. In external validation samples, we set the high-risk threshold to the same absolute value of predicted risk used in the Swedish sample.

† ECE was computed with 10 equal-frequency (quantile) bins.

‡ Taiwan dataset has a case–control structure, and statistics are reported naively, i.e., as if the fraction of cases were the population base rate. Thus metrics that involve the base rate—AUPRC, Brier, log-loss, PPV, F1, Accuracy—will be driven by the fraction of cases in the dataset, rather than the true population rate (which is unknown, given the case–control structure).

• The use of OLS to estimate mortality reduction is well justified, but causal inference should be more explicitly caveated, especially in observational settings with potential unmeasured confounding.

We have added a clearer caveat on unmeasured confounding, just as we introduce the OLS mortality reduction exercise.

5. Conclusions: Robustness, Validity, Reliability
The conclusions are generally well-supported. Internal validation is strong, and the model's identification of a novel high-risk group not captured by LVEF is compelling. However, external generalizability is limited by differing cohort designs and outcome definitions:
The U.S. dataset uses VT/VF as a surrogate, not actual SCD.
The Taiwanese cohort is case-control-based and limited in scope.
Thus, while the model shows promising transportability, caution is warranted in interpreting performance across dissimilar populations.

Thank you! We address these points in our response to the next comment, just below.

6. Suggested Improvements
Major:
• Obermeyer et al. must be commended on a meticulous study design and attention to detail in developing a practically useful model that may have several applications in clinical care. That being said, there is an attempt to present a large amount of information in one paper that can be fine-tuned to divert the readers' attention to some key focus areas. Some weaker aspects of the paper include the attempts at validation with cohorts that do not represent the training sample. The validation of the Swedish training set model (SCD defined with death certificated) against the US population (SCD, VT/VF identified by medical records linkage) may represent a fundamentally different population. More striking is the validation in the Taiwanese cohort which represented patients visiting the emergency room which is not at all representative of the public health sample of the Swedish training sample. Given that there are some very insightful observations made by the authors, some of these "forced" validation cohort analysis may be placed in the supplement or better eliminated altogether, with the focus of the paper being the development of an ECG model to detect SCD, and the generative model to understand potential high-risk ECG features.

We appreciate this feedback very much. We agree that there is a lot going on in the paper.

Your points about the validation datasets are very fair and we have added the caveats you note to the main text: (i) when we introduce the external validation datasets, we note immediately that their different designs and outcome definitions vs. the main Swedish dataset complicate

interpretation of performance metrics. (ii) we have done a better job of calling out the specific issues in each dataset, where we discuss the results: incentivized measurement of VF/VT in the US, highly selected sample of ED patients in Taiwan.

We hope it's ok that we respectfully choose to keep the external validation results in the main text, rather than moving them to a Supplement—though we have tried to condense as much as possible to maintain focus. Our rationale is that the differences across datasets - while “forced” in some ways, as you note - also provide two key advantages, particularly given some feedback we've received from clinicians in this space.

First, of course, understanding performance in diverse global populations, and on different medical equipment, is important. This isn't the norm in ML papers, because getting access to even one dataset is so hard. And even when you are lucky enough to get access to multiple datasets, the outcome variables often do not line up. As you note, this introduces complexity when it comes to interpreting the performance metrics in these external datasets.

Second, it is exactly this complexity that represents an advantage: the lack of perfect alignment in study outcomes is quite useful for studying sudden cardiac death in particular, because no one definition is perfect. Anecdotally, when we presented this paper in seminars before submission, most people did focus on the Swedish death certificates as the central outcome. But a substantial minority, especially cardiologists steeped in the traditional SCD epidemiology literature, were concerned about false positives and negatives, and would have been dissatisfied if we relied on death certificates alone. These people were reassured by the Taiwan results because they have more faith in detailed case studies. By contrast, interventionalists who focus on defibrillators cared less about the minutiae of definitions - but they were very interested in the presence of shockable rhythms like VF/VT, which are very common and well-measured in the US (likely better than Sweden, for reimbursement reasons, as we discuss below).

We absolutely acknowledge that the different cohort designs and outcomes create some difficulties for interpreting performance statistics. But we hope that they are offset by some advantages: the model's ability to predict many different outcomes related to SCD - including the arrhythmias that cause it, and detailed case study definitions common in the literature - across diverse populations triangulates a more complete picture of potential arrhythmic deaths. We think this is useful for the paper's longer-term impact in clinical audiences especially.

• Line 19-21: How do the authors reconcile the higher ECG model risk for SCD among patients with unknown LVEF when the survival was better than the low LVEF groups. Could this represent a higher risk patient for future decompensation and perhaps the follow-up duration was not enough to see the difference? Were there any attempts to look at other comorbidities for outcomes such as heart failure, or MI in the high versus low-risk ECG model?

This is an interesting question. We perform the disaggregated follow-up analysis you suggest: we add survival curves for the model's low- vs. high-risk patients, separately by LVEF status (reduced, normal, and unknown). The new figures confirm that patients with both high-risk ECGs <u>and</u> reduced LVEF have by far the worst outcomes; those with high-risk ECGs and either high or unknown LVEF are next-worst; and those with reduced LVEF whose ECGs are not high-risk do quite well. We have added this to Supplement II.B along with the other survival curves.

SCD survival by risk group

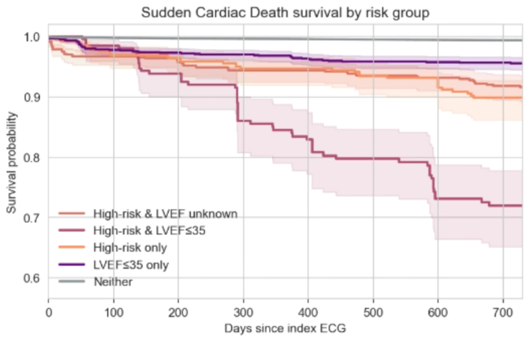

	0	100	200	300	400	500	600	700
**High-risk & LVEF unknown**								
At risk	817	656	617	581	552	525	500	481
Censored	16	157	190	217	246	268	291	303
Events	6	26	32	41	41	46	48	55
**High-risk & LVEF≤35**								
At risk	289	246	210	172	156	148	131	125
Censored	2	41	66	88	98	100	105	109
Events	0	4	15	31	37	43	55	57
**High-risk only**								
At risk	438	361	351	329	314	297	281	271
Censored	1	66	74	90	105	118	128	133
Events	0	12	14	20	20	24	30	35
**LVEF≤35 only**								
At risk	1626	1515	1478	1436	1392	1359	1329	1298
Censored	1	81	111	148	182	208	237	267
Events	4	35	42	47	57	64	65	66
**Neither**								
At risk	86104	82788	80863	78786	77034	75219	72996	71070
Censored	93	3242	5099	7140	8837	10603	12791	14670
Events	17	184	252	288	343	392	427	474

All-cause survival by risk group

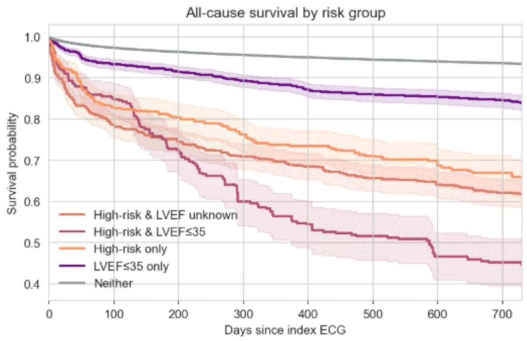

	0	100	200	300	400	500	600	700
**High-risk & LVEF unknown**								
At risk	817	656	617	581	552	525	500	481
Censored	0	2	8	15	24	29	41	45
Events	22	181	214	243	263	285	298	313
**High-risk & LVEF≤35**								
At risk	289	246	210	172	156	148	131	125
Censored	0	1	1	3	3	3	6	8
Events	2	44	80	116	132	140	154	158
**High-risk only**								
At risk	438	361	351	329	314	297	281	271
Censored	0	2	2	6	9	16	22	25
Events	1	76	86	104	116	126	136	143
**LVEF≤35 only**								
At risk	1626	1515	1478	1436	1392	1359	1329	1298
Censored	0	7	15	21	32	46	66	85
Events	5	109	138	174	207	226	236	248
**Neither**								
At risk	86104	82788	80863	78786	77034	75219	72996	71070
Censored	0	1114	2215	3667	4949	6333	8201	9790
Events	110	2312	3136	3761	4231	4662	5017	5354

We think the model's ability to predict risk among unknown-LVEF patients - despite their higher survival than low-LVEF patients - is the result of two factors. First, the population with unknown LVEF is large (77.5% of the sample), so the model has many instances to learn from; this drives better model performance. But second, <u>many high risk patients have been removed</u> from that set, i.e., patients with LVEF measured: doctors are not bad at predicting SCD risk, and when they identify high-risk patients they go ahead and measure the LVEF. So the remaining patients with unknown LVEF are, on average, lower risk as a result. Together these two factors allow the model to (i) perform well, by identifying very high-risk subsets, in a large population that is (ii) low-risk on average. We have added a brief summary of this discussion to the relevant section in Supplement I.B.

• Line 183: In the external validation with the US dataset, the model performance was suboptimal in both the US and Swedish datasets, with the model actually performing better in the US dataset. The authors attempt to explain this by citing differences in the incidence of VT/VF in the US population versus Sweden. While this explanation makes sense to some extent, it does not address the bigger issue of underfitting of the model. What do the authors feel could be the reasons for this?

Thanks for pushing us on this. You're absolutely right that higher incidence of VF/VT in the US is not, by itself, a good explanation for better AUC: ceteris paribus, changes in outcome rates should not change AUC. As noted above, we also see large differences in precision and recall, with better performance in the US as well (though for precision, the higher US incidence will drive improvements, unlike AUC) . We didn't do a good job of probing this in the original submission, and we have tried to clarify in the current draft, in the main text. We have also added a section to the Supplement (III.C) on this topic with more details.

Briefly, we believe that the model is faced with a classification task that is more difficult in Sweden than in the US, because of two key differences between the health systems and populations in those two countries.

The main driver relates to the well-known fact that Swedish patients are healthier to begin with (e.g., life expectancy of 81.7 years vs. 75.8 in the US), and also more likely to have earlier intervention and better disease management thanks to lower barriers to care. This likely compresses the risk distribution captured by ECGs, and with fewer obviously high-risk people, it is more difficult for the model to distinguish future SCDs from others: everyone looks similarly healthy, so the model has to "work harder" to distinguish high- vs. low-risk ECGs. By contrast, the US population is likely to have a wider distribution of risk captured by the ECG, reflecting less equal access to care and outcomes. Thus the same small differences the model picked up on in Sweden are amplified in the US, making it easier to discriminate.

To show this empirically, we show the distribution of model predicted risk in Sweden vs. the US directly, as a histogram (with each population normalized to 100% so heights reflect percent of ECGs rather than counts; binning is identical, with 500 equal-width bins spanning 0–0.20 risk predictions). The vertical dashed line marks the absolute high-risk threshold defined in the Swedish cohort (4.0%). The US mean risk is far higher (0.029 vs. 0.005), which we expect from the higher base rate of VF/VT. Just as importantly, the US risk distribution is far wider (SD 0.041 vs. 0.009), supporting the idea of differences in distribution, not just mean. We include the figure, with a condensed version of this discussion, in Supplement III.C.

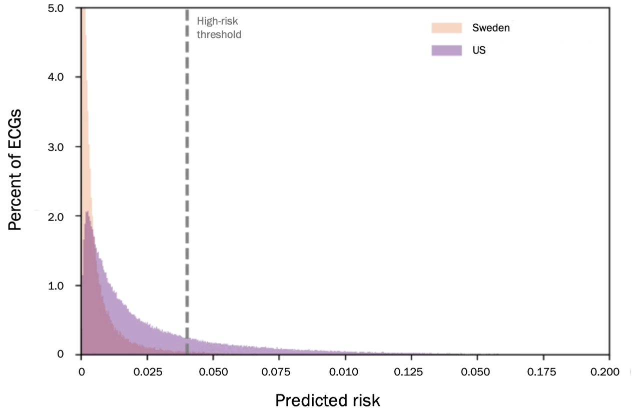

One other potential problem in Sweden is that testing for VF/VT is likely far less common than in the US: insurance reimbursement rates are far higher when VF/VT is present in the US (they are “CC/MCC”’s - see here), creating strong financial incentives to test, e.g., with Holter monitors, cardiac monitoring during hospitalization, and document. (We attempted to produce some empirical results on the frequency of measurement across settings, but unfortunately, ambulatory ECG monitoring in Sweden is either very infrequently recorded in EHRs, or very infrequently done: we were only able to identify a handful of patients with Holter monitors, for example, over the span of our dataset.) This means the VF/VT label is likely *more aligned with the true underlying incidence* of VF/VT in the US, which increases the AUC of the model - which is quite good at predicting arrhythmias, because it was trained on death certificates. As a concrete example, imagine that the model correctly flags a high-risk Swedish patient who goes on to have VF/VT. Because that person is less likely to be tested, the model is more likely to appear “wrong” in Sweden data, decreasing AUC - but in this case, the model prediction was right and the label was wrong. In the US dataset, the same patient is more likely to be tested, and the model is more likely to be vindicated by a recorded episode of VF/VT. So this story is not so much underfitting, but rather differential measurement of the outcome across datasets.

• To address the imbalances in the datasets, it would be ideal for the authors to report precision-recall curves, and F1 scores.

Done for all datasets, and included in Supplement III.

• Explore subgroup calibration and risk prediction performance across clinically relevant strata.

We now show a calibration graph (similar to the ones shown above, comparing Sweden vs external datasets) for clinically-relevant subgroups in Sweden in Supplement I.A. This indicates good calibration, except in the subset with LVEF, who have higher event rates than predicted. This echoes the results mentioned in the main text (Figure 3), showing that LVEF adds independent predictive power to the ECG-based risk predictions. We also replicate Figure 1 from the main text, to show how different choices of high-risk group definition affect these same subgroups. In addition, we have replicated the calibration metrics (and other performance metrics, for completeness) suggested by you and the other reviewer, in a new Table for the same subgroups. This is also in Supplement I.A.

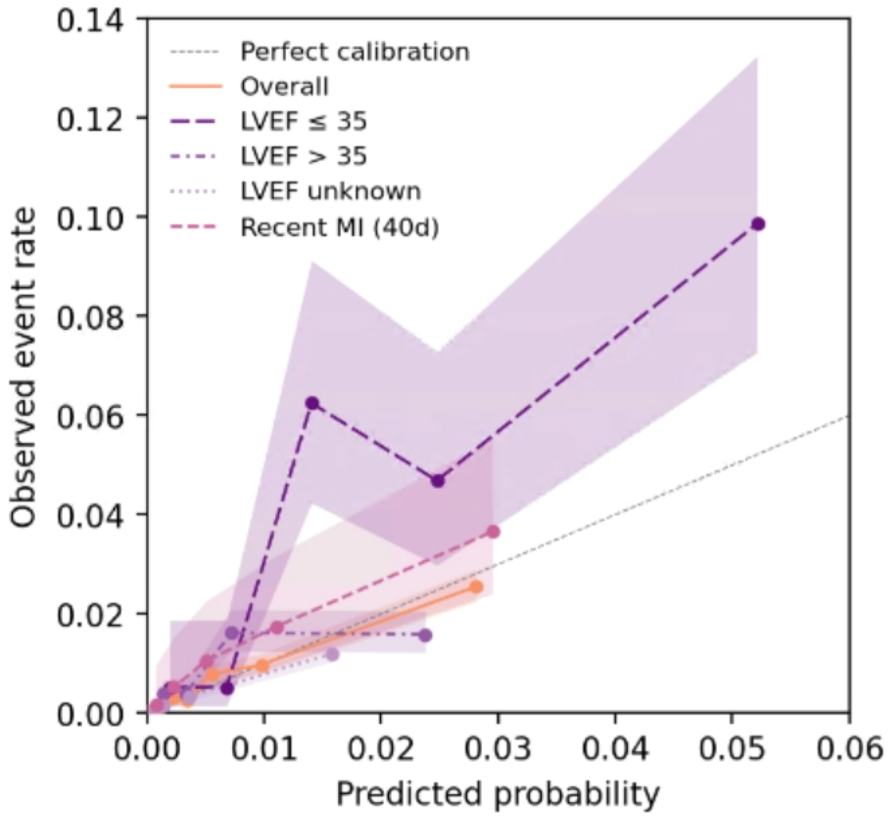

Perfect calibration
Overall
LVEF ≤ 35
LVEF > 35
LVEF unknown
Recent MI (40d)
Observed event rate
Predicted probability
0.14
0.12
0.10
0.08
0.06
0.04
0.02
0.00
0.00 0.01 0.02 0.03 0.04 0.05 0.06

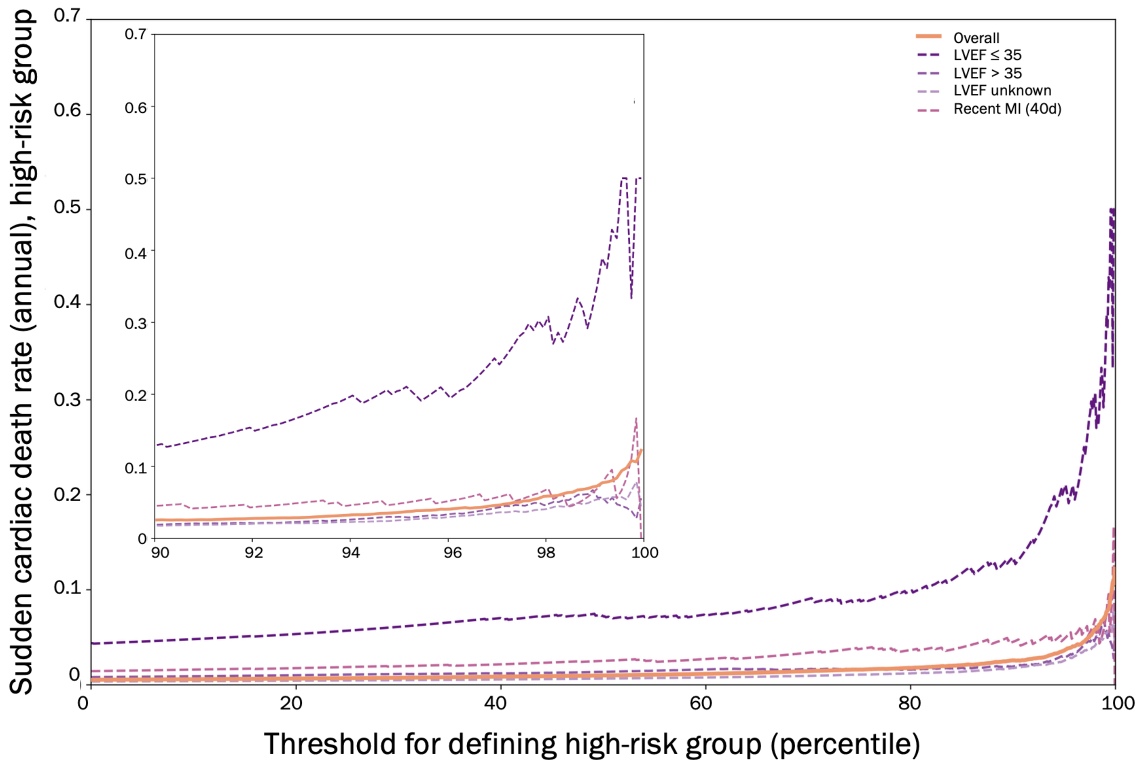

Overall
LVEF ≤ 35
LVEF > 35
LVEF unknown
Recent MI (40d)
Sudden cardiac death rate (annual), high-risk group
Threshold for defining high-risk group (percentile)
0.7
0.6
0.5
0.4
0.3
0.2
0.1
0
0 20 40 60 80 100
90 92 94 96 98 100

Population statistics	*Clinically relevant subgroups*				
Sample	All	LVEF ≤35%	LVEF >35%	No EF recorded	Recent MI (40 days)
N	89,414	1922	17,631	69,861	2,870
Base rate of outcome	0.0052	0.0437	0.008	0.0034	0.0143
Calibration metrics					
Brier Score	0.005	0.041	0.008	0.003	0.014
	(0.005, 0.006)	(0.033, 0.050)	(0.007, 0.009)	(0.003, 0.004)	(0.010, 0.018)
Expected Calibration Error (ECE)†	0.001	0.025	0.004	0.001	0.005
	(0.001, 0.001)	(0.018, 0.035)	(0.003, 0.006)	(0.001, 0.001)	(0.004, 0.011)
Threshold-independent classification metrics					
AUC	0.837	0.741	0.736	0.841	0.758
	(0.810, 0.862)	(0.691, 0.786)	(0.705, 0.770)	(0.816, 0.863)	(0.682, 0.821)
AUPRC	0.04	0.154	0.023	0.029	0.046
	(0.031, 0.053)	(0.101, 0.232)	(0.017, 0.035)	(0.020, 0.044)	(0.026, 0.106)
Threshold-dependent classification metrics					
High risk fraction	0.0176	0.152	0.0249	0.012	0.0369
PPV	0.061	0.116	0.046	0.049	0.057
	(0.050, 0.070)	(0.081, 0.151)	(0.028, 0.066)	(0.034, 0.064)	(0.019, 0.108)
Sensitivity	0.204	0.405	0.142	0.171	0.146
	(0.168, 0.239)	(0.303, 0.507)	(0.089, 0.200)	(0.123, 0.217)	(0.048, 0.262)
F1 Score	0.093	0.181	0.069	0.076	0.082
	(0.077, 0.108)	(0.128, 0.228)	(0.043, 0.099)	(0.054, 0.098)	(0.027, 0.151)
Accuracy	0.979	0.84	0.969	0.986	0.953
	(0.979, 0.980)	(0.823, 0.856)	(0.967, 0.972)	(0.985, 0.987)	(0.945, 0.961)
Balanced Accuracy	0.594	0.632	0.559	0.58	0.555
	(0.576, 0.611)	(0.580, 0.683)	(0.533, 0.587)	(0.556, 0.603)	(0.506, 0.614)

• The correlation of AI model output performance to specific signal feature changes has been elusive, and the report of the change in aVL impacting SCD risk is striking and almost startling. Generative AI models may find different signal features associated with an outcome or phenotype based on the initial "seed" ECG fed into the model. It is a bit surprising that changes in a single lead (aVL) were so strongly associated with the model's overall performance. Was this generative experiment repeated and was the finding in aVL consistent, or were changes in other leads more prominent in some experiments? Using lead aVL alone, what is the performance of the model – how much of

the total impact of the model comes from this lead alone, and how well do other leads perform in isolation?

These are both great questions. We answer them via new analyses that are now shown in Supplement V.H, and mentioned in the main text.

Q1: "Was this generative experiment repeated and was the finding in aVL consistent, or were changes in other leads more prominent in some experiments?"
Yes, we did perform several runs of the generative experiment, and qualitatively, we found the aVL changes to be consistently the most visually lead of the high-risk morphs. But to answer this question more rigorously, we designed a new experiment to measure predictive power of the 1st and 2nd difference features, when measured in leads other than aVL. To implement this, we simply calculate the 1st and 2nd difference features in each lead, and compare their predictive performance using our two main metrics: AUC and PPV (rate of sudden cardiac death in the highest-risk 1.8%, for comparison to the results in our main analysis). The results are in the Table below (and reproduced in Supplement V.H).

Across leads, we see that the features calculated in aVL do have the highest combined PPVs for sudden death; however <u>most leads generate features with roughly similar performance to the aVL features, so it almost doesn't matter which lead we use</u> (with some exceptions: aVR and the right-sided precordial leads are less good). This challenged our prior belief - thank you! - and highlights an interesting difference between features noticeable by humans and the model: we found the aVL differences very striking, but this lead turns out to be only slightly more quantitatively important than other leads. We think this suggests that the features are capturing some diffuse process happening throughout the myocardium.

Q2: "Using lead aVL alone, what is the performance of the model – how much of the total impact of the model comes from this lead alone, and how well do other leads perform in isolation?"
To answer this question, we trained 12 new models, each using the full waveform from one ECG lead. We then compared the AUCs and PPVs of these single-lead models to assess whether there is more or less information in one particular lead. The results (below, and in Supplement V.H) were interesting - thank you for suggesting this! <u>relative to the full 12-lead model, with AUC 0.84, the single-lead predictions range from AUCs of 0.78 (in III and aVF) to 0.82 (in II, aVR, V3-6), indicating that discriminative power is broadly present across all leads</u>. The PPVs (SCD rate in the high-risk group) for these single-lead models, however, were substantially lower than the full 12-lead model, reflecting worse performance in the tails: using the same 1.8% threshold for defining the high-risk group as in the main analysis, single-lead PPVs range from 2.1% (in III) to 4.9% (V3), compared to 6.1% for the full 12-lead model. Together these results suggest that the model finds signal diffusely and throughout many different leads, not just aVL, and that putting all leads together does better for identifying the highest-risk ECGs.

One last note on the comparison of the first- and second-difference feature AUCs (around 0.60) vs. the full model AUC (0.84): the features are only a simple proxy measure for the morphology visualized by the generative model, which is only a subset of the signal used by the predictive model. Obviously, the full model is much richer than any one feature, and its AUC is much higher as a result. These features are highlighted not for their predictive power (though it's important to establish that they have predictive power alone), but rather as a way to generate physiological hypotheses. We have added a concise summary of these points to Supplement V.H.

Lead	Feature	AUC (95 % CI)	SCD rate, high-risk	Lead	Feature	AUC (95 % CI)	SCD rate, high-risk
I	1st difference	0.58 (0.55, 0.61)	0.011	V1	1st difference	0.59 (0.56, 0.62)	0.014
	2nd difference	0.60 (0.57, 0.62)	0.013		2nd difference	0.60 (0.57, 0.63)	0.01
	Single-lead model	0.79 (0.77, 0.81)	0.034		Single-lead model	0.80 (0.78, 0.82)	0.034
II	1st difference	0.53 (0.50, 0.56)	0.01	V2	1st difference	0.54 (0.51, 0.56)	0.009
	2nd difference	0.58 (0.55, 0.61)	0.013		2nd difference	0.52 (0.49, 0.55)	0.004
	Single-lead model	0.82 (0.80, 0.84)	0.036		Single-lead model	0.80 (0.78, 0.82)	0.034
III	1st difference	0.61 (0.58, 0.63)	0.014	V3	1st difference	0.50 (0.47, 0.52)	0.006
	2nd difference	0.63 (0.60, 0.65)	0.012		2nd difference	0.52 (0.49, 0.54)	0.006
	Single-lead model	0.78 (0.76, 0.79)	0.021		Single-lead model	0.82 (0.80, 0.83)	0.041
aVR	1st difference	0.49 (0.46, 0.51)	0.006	V4	1st difference	0.56 (0.53, 0.58)	0.009
	2nd difference	0.55 (0.52, 0.57)	0.005		2nd difference	0.59 (0.57, 0.62)	0.008
	Single-lead model	0.82 (0.80, 0.83)	0.038		Single-lead model	0.82 (0.80, 0.83)	0.041
**aVL**	**1st difference**	**0.58 (0.56, 0.61)**	**0.014**	V5	1st difference	0.59 (0.57, 0.62)	0.013
	**2nd difference**	**0.58 (0.55, 0.60)**	**0.016**		2nd difference	0.62 (0.60, 0.65)	0.016
	**Single-lead model**	**0.79 (0.77, 0.81)**	**0.030**		Single-lead model	0.82 (0.80, 0.83)	0.047
aVF	1st difference	0.58 (0.55, 0.61)	0.012	V6	1st difference	0.57 (0.54, 0.60)	0.014
	2nd difference	0.60 (0.57, 0.63)	0.013		2nd difference	0.60 (0.57, 0.63)	0.012
	Single-lead model	0.78 (0.76, 0.80)	0.033		Single-lead model	0.82 (0.80, 0.84)	0.037

Minor:

• Include calibration curves or Brier scores to demonstrate how well predicted probabilities reflect actual risk.

Both added as noted above, in Supplement I (for subgroups in Sweden) and III (for comparison to external validation samples).

• Report follow-up duration and censoring strategy in greater detail.

We were remiss in not reporting this in the original submission, and appreciate the catch. We have added detail to the manuscript on this point, mainly in the Methods (though also in the Supplement, regarding survival analysis and model training). We summarize those details here.

Main analysis. We start observing ECGs on January 1, 2012, and our linkage to death certificate data ends on December 31, 2016. Our primary outcomes, sudden cardiac death and all-cause mortality outcomes, are defined over a period of one year following each ECG. So ECGs in the final year of data (2016) can be censored by the end of our death certificate data. (We are not particularly worried about other kinds of incomplete follow-up, as official death certificates are obtained via centralized Swedish public health records which have been quite reliable going back to the 17th century!).

Without death certificates after 2016, we would normally have to consider all ECGs censored if the patients was still alive on December 31, 2016; however, we fortunately have access to full electronic health record data from December 2016 to 2022, which allow us to observe if a patient is still interacting with the health care system. Thus for ECGs from 2016, even those without a death certificate, if we see a clinical encounter in the EHR after the one-year follow up period, we can confidently label the ECG as “no one-year SCD” and “no one-year all-cause mortality”. We thus update our follow-up duration for these observations to that encounter. Only ECGs for which we observe no EHR data after one year are considered censored. This means only 6,090 out of 117,039 ECG records in the training set and 4,579 out of 89,414 ECG records in the validation set are censored for one-year mortality outcomes. These observations are excluded from outcome evaluation metrics throughout the manuscript to ensure that performance estimates reflect accurately observed events. The median and IQR follow-up durations have been added to Table 2 in the Methods.

	**All**	**By outcome**	
		Sudden cardiac death (death certificates)	All others
Patients	29,462	112	29,350
ECGs	94,736	538	94,198
ECG Count per Patient (mean)	3.22	4.08	3.2

Median days from ECG to end of follow-up period (25-75th pctile)	1980 (996-2700)	390 (95-798)	1980 (1020-2700)

Survival analyses. Here, we transparently display sample sizes of eligibles and deaths along with the graphs. Of note, due to an oversight on our part, the survival curves displayed in our original submission did not incorporate the post-2016 EHR data to reduce censoring; in the new version, we have fixed this. The mean estimates are very similar, but the confidence intervals look much tighter, as expected.

SCD survival: ECG High- vs. Low-risk

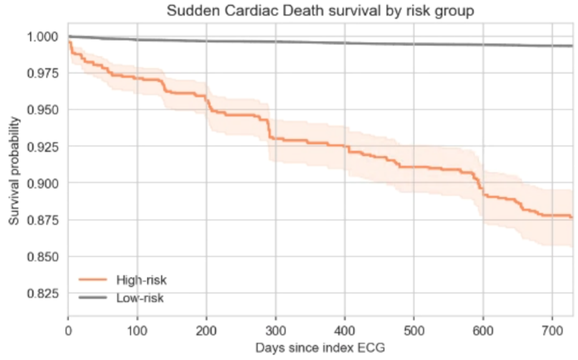

**High-risk**								
At risk	1544	1263	1178	1082	1022	970	912	877
Censored	19	264	330	395	449	486	524	545
Events	6	42	61	92	98	113	133	147
**Low-risk**								
At risk	87730	84303	82341	80222	78426	76578	74325	72368
Censored	94	3323	5210	7288	9019	10811	13028	14937
Events	21	219	294	335	400	456	492	540

All-cause survival: ECG High- vs. Low-risk

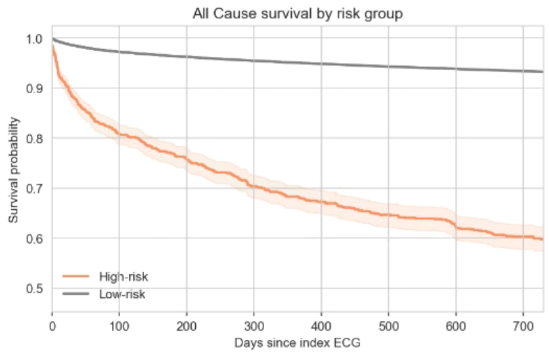

**High-risk**								
At risk	1544	1263	1178	1082	1022	970	912	877
Censored	0	5	11	24	36	48	68	77
Events	25	301	380	463	511	551	589	615
**Low-risk**								
At risk	87730	84303	82341	80222	78426	76578	74325	72368
Censored	0	1120	2229	3684	4974	6368	8254	9860
Events	115	2422	3275	3939	4445	4899	5266	5617

SCD survival: By LVEF status

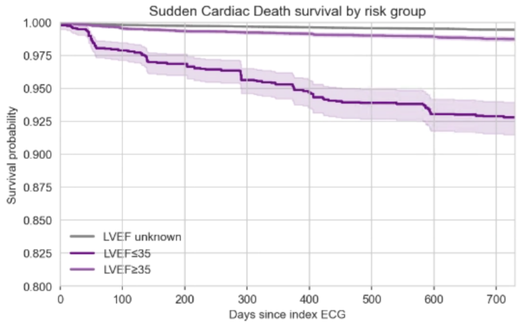

**LVEF unknown**								
At risk	69748	66993	65358	63513	62010	60462	58501	56820
Censored	90	2728	4319	6131	7594	9106	11039	12684
Events	23	140	184	217	257	293	321	357
**LVEF≤35**								
At risk	1915	1761	1688	1608	1548	1507	1460	1423
Censored	3	122	177	236	280	308	342	376
Events	4	39	57	78	94	107	120	123
**LVEF≥35**								
At risk	17611	16812	16473	16183	15890	15579	15276	15002
Censored	20	738	1045	1317	1595	1884	2172	2423
Events	0	81	113	131	146	168	183	206

All-cause survival: By LVEF status

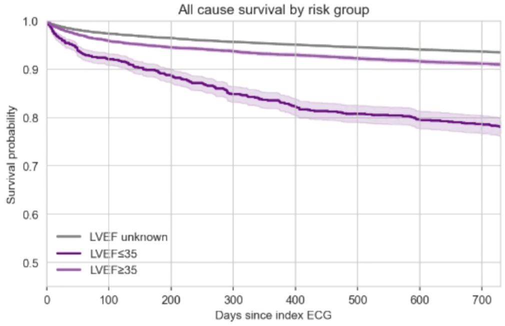

**LVEF unknown**								
At risk	69748	66993	65358	63513	62010	60462	58501	56820
Censored	0	1033	2030	3341	4464	5670	7352	8754
Events	113	1835	2473	3007	3387	3729	4008	4287
**LVEF≤35**								
At risk	1915	1761	1688	1608	1548	1507	1460	1423
Censored	0	8	16	24	35	49	72	93
Events	7	153	218	290	339	366	390	406
**LVEF≥35**								
At risk	17611	16812	16473	16183	15890	15579	15276	15002
Censored	0	84	194	343	511	697	898	1090
Events	20	735	964	1105	1230	1355	1457	1539

Model training. In training our model, we predict outcomes ranging from 6-month to 24-month sudden cardiac death and use the same approach to defining censoring over the relevant time frame. Censored cases are masked to prevent them from influencing gradient computations, as their outcomes are not definitively known within the prediction horizon.

External validation. Censoring is less important in both our external validation datasets. There is no censoring in the US dataset, as the follow up period for health records is over 2 years after the last ECG we observe, and we compute only one-year incidence of VF/VT. Similarly, due to the case-control nature of the Taiwan dataset, there is no censoring.

• Grammatical/spelling errors: Line 503

Fixed.

7. References: Appropriate Credit to Previous Work?
Yes, the manuscript references a comprehensive array of literature, including foundational ICD trials, LVEF limitations, and recent AI-ECG applications.

8. Clarity and Context
Abstract: Accurate, informative, and well-written. It effectively communicates the problem, methods, key findings, and implications.

Introduction: Thorough and contextual. It clearly outlines the limitations of current biomarkers and motivates the development of an ECG-based deep learning model.

Thank you for these comments!

Conclusion: The discussion of scientific and clinical implications is balanced and insightful. The hypothesis about myocardial fibrosis and conduction abnormalities is intriguing and biologically plausible, though it should be clearly labeled as speculative

Thanks, we have revised to emphasize the very speculative nature of the hypotheses in that section. We also note that future work to correlate ECG waveforms with biopsy findings could be valuable for identification of a broad set of pathophysiological factors in high-risk patients.

Recommendation: Major Revisions
The manuscript is a high-quality and innovative contribution with substantial clinical and scientific value. Revisions to improve clarity on external validation limitations, expand evaluation metrics, and fine-tune presentation of exploratory findings will enhance its impact and interpretability.

We appreciate the careful review and thoughtful comments.

Referee #2 (Remarks to the Author):

This is a novel and exemplary study showcasing how we can derive new, human interpretable knowledge in medical science, specifically, about ECG and sudden cardiac death (SCD), from deep learning. The authors developed a deep learning model with a large dataset to predict SCD from ECGs, and validated the model in external datasets internationally from different cohorts at different locations. Through a series of interesting statistical analysis, the authors showed that: 1) the model can accurately predict both SCD and VF/VT, despite that it was trained on death certificates; 2) the model is specific to predict arrhythmic death; 3) the predicted high-risk group can benefit from ICDs, but the risk has not been fully captured by the widely used metric LVEF. Until here, the manuscript has told an excellent research story, like previous publications on this topic, but the following analysis has made this manuscript extraordinary. The authors exploited what exact patterns / biomarkers in ECGs are predictors learned by the model to predict SCD. By visualizing ECG waveform morphs, they discovered that a missing S-wave in lead aVL is a strong predictor for SCD.

Thank you for this very encouraging feedback.

My comments are below:
1. The number of patients is not mentioned anywhere in the main text. This is a particularly important factor people consider when evaluating the performance metrics and the validity of results in clinical studies. I think this should be added to the abstract and main text, instead of remaining in the methods and supplements.

Thanks - we have moved the sample size to accompany the results in the main text, and provide additional detail in the Methods (and a CONSORT-style diagram in Supplement VII.A, reproduced below). Briefly:

- The main results are produced in a validation set of 89,414 ECGs from 29,108 Swedish patients, all under 80 years old at the time of ECG and without defibrillators. (This represents a 30% random sample, originally comprised on 37,601 patients and all 131,280 of their ECGs; we then restrict to those under 80 years old at the time of the ECG and without defibrillators to get the numbers above.)
- The model was trained in another 30% sample, all ages but restricted to those without defibrillators, consisting of 34,178 patients and all 117,039 of their ECGs to train.
- The remaining 40% of the full dataset remain untouched in a “lock box” that no one on the research team accessed after receiving the data. If we are lucky enough to have this manuscript accepted, we propose retraining the algorithm on the full 60% of patients whose data we have accessed - and thus in whom we created the possibility of inadvertent overfitting - and present results for the remaining 40% lock box with no modifications to the model.

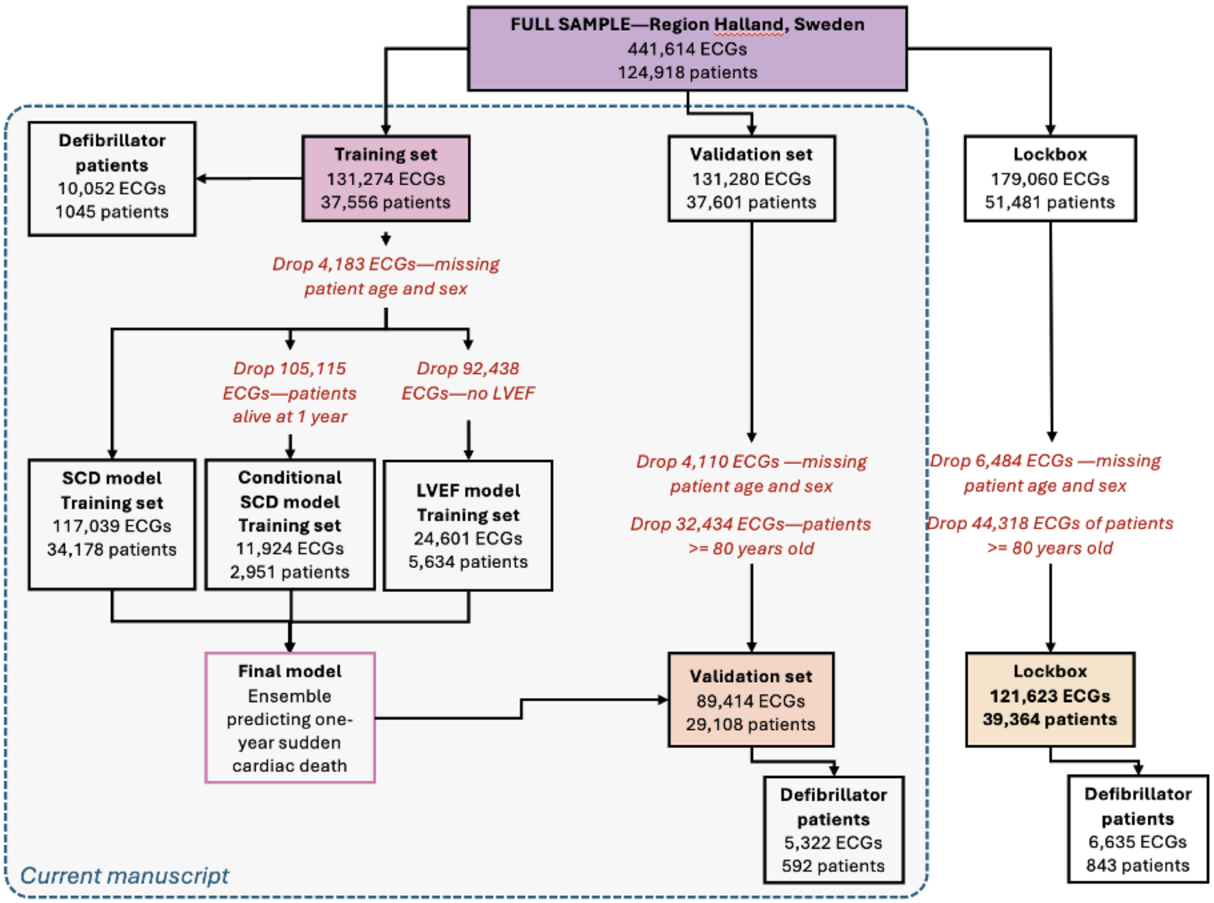

2. The only performance metric reported in the manuscript is AUC. AUC is known to be sensitive to class imbalance (https://doi.org/10.1371/journal.pone.0118432), and SCD is indeed very rare in the population. Other metrics that are less sensitive to class imbalance should be reported as well in complementary to AUC, like F score, balanced accuracy, PR curves, etc.

We have added the requested figures and metrics to the Supplement (III), where they complement the more clinically-oriented metrics presented in the main text. Specifically, we now show calibration curves, as well as ROC and precision-recall curves, for both Sweden and the external validation samples (III.A). We also present all the requested metrics, as well as some additional ones, in a new table: AUC and AUPRC statistics, F1 scores, accuracy and balanced accuracy (in response to another reviewer, who had similar feedback), as well as Brier score and expected calibration error (III.B). In response to your next comment, we now use the absolute risk threshold from Sweden to define discrete high-risk groups in the external validation datasets (rather than classifying the top 1.8% as high-risk as we did in the initial submission, which as you noted requires knowledge of the population distribution).

This was a very valuable exercise, and we are grateful to you for suggesting it - we summarize some of the new insights here (and have inserted these into the main text and Supplement where relevant).

Calibration. As shown in the calibration curves, the model is well calibrated in the Swedish validation set for predicting sudden cardiac death. The overall Brier score is 0.005 (95% CI: 0.005, 0.006). To give some sense of a baseline against which to compare this number, we calculate the model's Brier score in the subset of those with LVEF recorded, 0.011 (95% 0.010, 0.013), which is very close to LVEF, 0.011 (95% CI: 0.010, 0.012; to get LVEF into a probability suitable for Brier calculation, we include it in a logistic regression mode with age and sex in the training set, and calculate Brier score in the validation set, just as we do for the ECG model.) Results for additional clinical subgroups are shown in Supplement I. We can also assess calibration in the Taiwanese dataset, which we report naively, treating the case-control sample as a 'population' (with a base rate of the outcome is 1.6%, vs. 0.5% in Sweden, driven by the ratio of cases to controls; this is arbitrary, but likely not far from realistic population rates). With these caveats in mind, calibration remains good in Taiwan: the risk increase is linear at 45-degrees, i.e. observed risk rises proportionally and linearly with predicted risk. The intercept is slightly shifted above the 45-degree line, but the 95% confidence intervals include it. Of note, the highest-risk decile in Taiwan is almost 2x as risky as the highest-risk decile in Sweden, but calibration is linear and unaffected.

Because the model was not trained to predict VF/VT, we should not necessarily expect perfect calibration on the 45-degree line for this outcome. However, given the physiological relationship between VF/VT and the prediction target of SCD, we can make some inferences regarding what the graph should look like. First, VF/VT and SCD should be strongly positively correlated: VF/VT causes sudden cardiac death. Reassuringly, we see the monotonic increases in VF/VT vs. predicted risk we expect as a result, i.e., higher risk bins have higher rates of VF/VT. Second, not all VF/VT leads to SCD (these rhythms can self-resolve), so SCD is a subset of VF/VT. Thus we should expect a calibration slope >1, i.e., above the 45-degree line, which is also what we observe. Again here, we emphasize that the highest-risk decile in the US is 4x as risky as the highest-risk decile in Sweden, but calibration remains linear.

Precision and recall. The precision-recall curve for sudden cardiac death shows clinically high precision (PPV) in the top few percent with reasonable sensitivity. At our preferred threshold (based on ICD RCTs, with median control-arm SCD rates of 4.9%), precision is 6.1% with a sensitivity of 20.4% (see table). As a point of comparison, reduced LVEF has a precision of 4.4% and a sensitivity of 18.1% in our sample.[2] The corresponding F1 scores are 0.093 (95% CI: 0.077, 0.108) for our model vs. 0.070 (95% CI: 0.058, 0.083) for LVEF. In Taiwan (with the same caveats regarding case-control design noted above), the precision-recall curve is at least as good as in Sweden, with very similar PPV, sensitivity, and F1 scores.

[2] This is slightly different from the 21.7% of SCD with reduced LVEF in Table 1, because that number is produced in the entire validation set including those with defibrillators. We calculate model performance metrics on the non-defibrillator population to avoid improperly including the treatment effect of the defibrillator on outcomes

The difference between the Swedish vs. US precision-recall curves is quite striking - more so than with AUC, because here the base rate affects precision - with US precision and recall both far better than in Sweden. We provide more detail on this in response your next question, on defining the high-risk groups for the US and Sweden datasets, but since it's relevant here, we'll summarize what we think drives better performance in the US: the higher risk, and also higher risk variance, of the US population, likely reflecting differences in general health and health equity. The simplest way to see this is by comparing the mean and SD of predicted risk: in the US these are 0.029 and 0.041, while in Sweden they are 0.005 and 0.009, respectively. The low average risk makes precision lower, and the compressed risk distribution in Sweden makes the model's discrimination task harder. Another factor that may contribute is that VF/VT are likely better measured in the US, due to strong financial incentives. This could improve model performance, particularly if high-risk patients are measured more in the US: if our model (trained on death certificates) accurately predicts VF/VT in a patient who does have VF/VT, it will often appear "wrong" in Sweden—because VF/VT happens but is not recorded. Conversely, it will do better in the US, where these events are more likely to be recorded.

Calibration curves (decile bins)

Outcome: Sudden cardiac death — Datasets: Sweden vs. Taiwan

Outcome: VF/VT — Datasets: Sweden vs. US

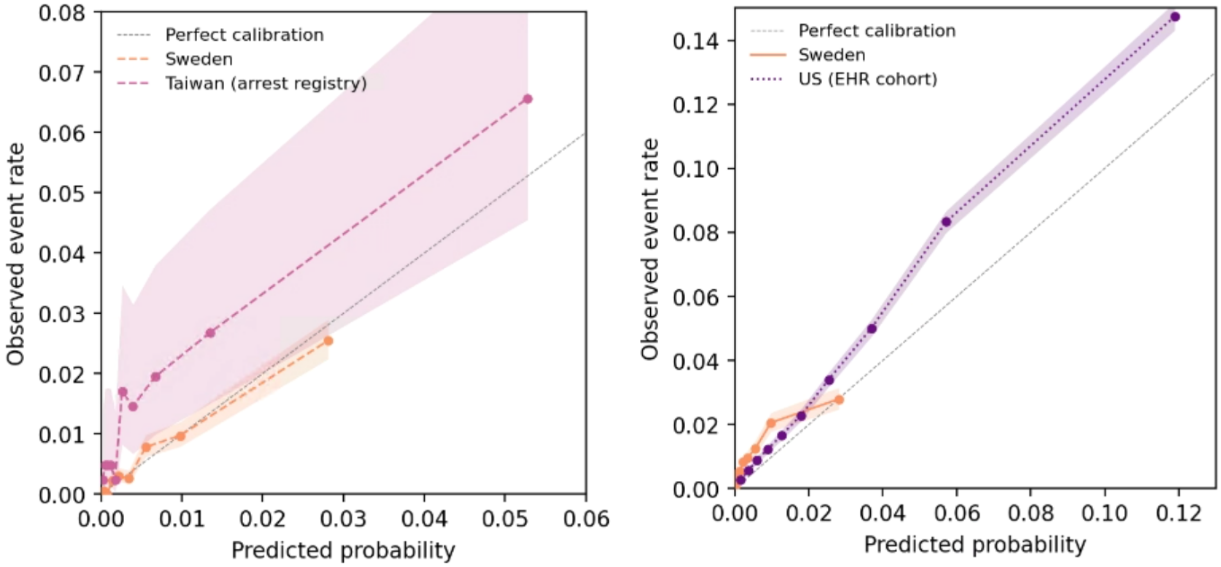

Receiver-operating-characteristic curves

Outcome: Sudden cardiac death

Datasets: Sweden vs. Taiwan

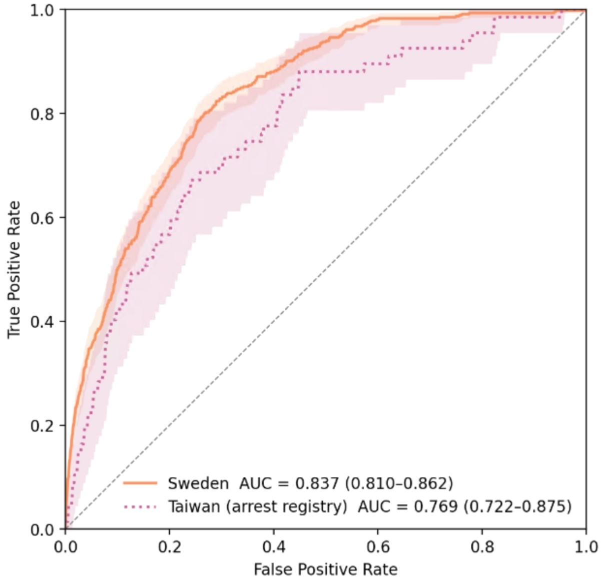

Outcome: VF/VT

Datasets: Sweden vs. US

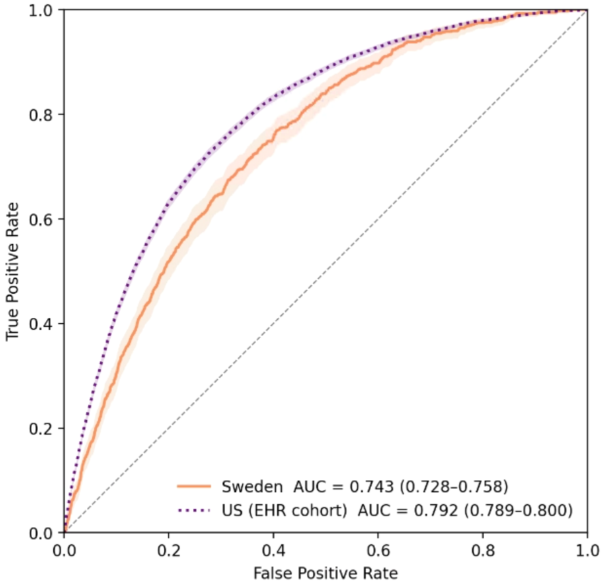

Precision-recall curves

Outcome: Sudden cardiac death

Datasets: Sweden vs. Taiwan

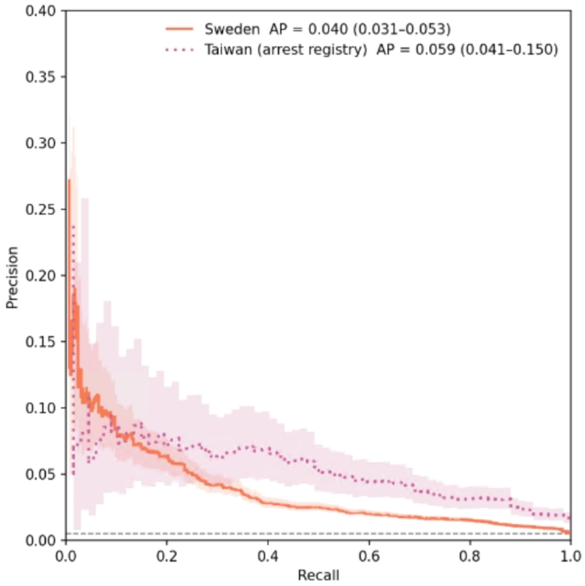

Outcome: VF/VT

Datasets: Sweden vs. US

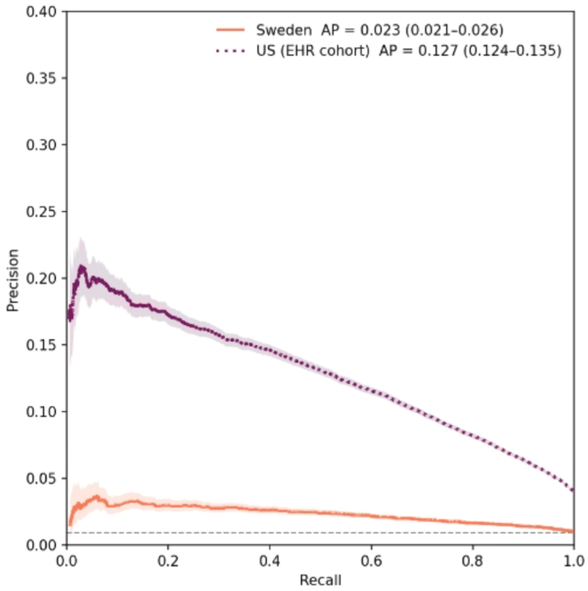

Additional performance metrics

Outcome	Sudden cardiac death		VF/VT	
Population	*Sweden*	*Taiwan*[‡]	*Sweden*	*US*
n	89,414	4107	89,414	251,858
Base rate of outcome	0.0052	0.0163	0.0094	0.0384
Calibration metrics				
Brier Score	0.005	0.016	0.009	0.036
	(0.005, 0.006)	(0.013, 0.023)	(0.009, 0.010)	(0.035, 0.037)
Expected Calibration Error (ECE)[†]	0.001	0.008	0.004	0.01
	(0.001, 0.001)	(0.006, 0.015)	(0.004, 0.005)	(0.009, 0.010)
Threshold-independent classification metrics				
AUC	0.837	0.769	0.743	0.792
	(0.810, 0.862)	(0.722, 0.875)	(0.728, 0.758)	(0.789, 0.800)
AUPRC	0.04	0.059	0.023	0.127
	(0.031, 0.053)	(0.041, 0.150)	(0.021, 0.026)	(0.124, 0.135)
Threshold-dependent classification metrics				
High risk fraction*	0.018	0.054	0.018	0.226
PPV	0.061	0.067	0.034	0.109
	(0.050, 0.070)	(0.042, 0.120)	(0.025, 0.043)	(0.107, 0.113)
Sensitivity	0.204	0.224	0.063	0.643
	(0.168, 0.239)	(0.145, 0.451)	(0.046, 0.080)	(0.635, 0.659)
F1 Score	0.093	0.103	0.044	0.186
	(0.077, 0.108)	(0.065, 0.177)	(0.033, 0.056)	(0.183, 0.192)
Accuracy	0.979	0.937	0.974	0.785
	(0.979, 0.980)	(0.931, 0.953)	(0.973, 0.975)	(0.783, 0.788)
Balanced Accuracy	0.594	0.586	0.523	0.717
	(0.576, 0.611)	(0.546, 0.699)	(0.515, 0.531)	(0.713, 0.725)

Note: 95% CIs are computed via 1,000 bootstrap resamples.

* High-risk threshold is set as described in the main text in the Swedish sample, based on defibrillator trial control group rates of sudden cardiac death. In external validation samples, we set the high-risk threshold to the same absolute value of predicted risk used in the Swedish sample.

[†] ECE was computed with 10 equal-frequency (quantile) bins.

[‡] Taiwan dataset has a case–control structure, and statistics are reported naively, i.e., as if the fraction of cases were the population base rate. Thus metrics that involve the base rate—AUPRC, Brier, log-loss, PPV, F1, Accuracy—will be driven by the fraction of cases in the dataset, rather than the true population rate (which is unknown, given the case–control structure).

3. The authors chose a threshold based on population percentile. I agree on the rationale how this was chosen, but how did you apply this threshold on other cohorts (US and Taiwan)? From figure 2b, it seems that you still chose the top 1.8% as high-risk groups. If a model needs information about the patient distribution to set threshold, then for new cohorts with no prior distribution information, how will you use it?

Thank you, this is a really good point. We now set the high-risk threshold for the external validation datasets using the same absolute value of predicted risk as in the Swedish sample (i.e., the absolute risk threshold that classifies 1.8% of the Swedish sample high risk, which we chose based on ICD RCTs). This avoids the need for distributional information about new populations. Those results are mentioned in the main text, and also shown in the table above (in response to your last comment; this is also in Supplement III.B).

In Taiwan, it's worth noting that the case-control design affects interpretation of all metrics that involve classification of a high-risk group (as opposed to the analyses in the main text, which focus on discrimination of cases vs. controls). In particular, the "population rate" of SCD (1.6%) is driven simply by the ratio of sampled cases to controls in this dataset, which is somewhat arbitrary, and not a true population rate. With that in mind, as shown in the table above, performance in Taiwan matches performance in Sweden closely, with very similar PPV, sensitivity, etc.

In the US, the absolute risk threshold produces one striking change: the high-risk group is much larger, 22.6% of the sample (vs. 1.8% in Sweden). Despite that, the US high-risk group still has a rate of VF/VT of 10.9% per year (95% CI: 10.7-11.3%), which is both high in absolute terms and far higher than the far smaller Swedish high risk group (3.4%, 95% CI: 2.5-4.3%). We reproduce the calibration curve from above for reference. The absolute high-risk threshold is at 4.0% on the x-axis. As you can see, there is a long tail of US patients who are far higher risk than the riskiest Swedish patients—e.g., the highest-risk decile in the US is 4x as risky as the highest-risk decile in Sweden, but the relationship of observed vs. predicted risk remains linear and smooth.

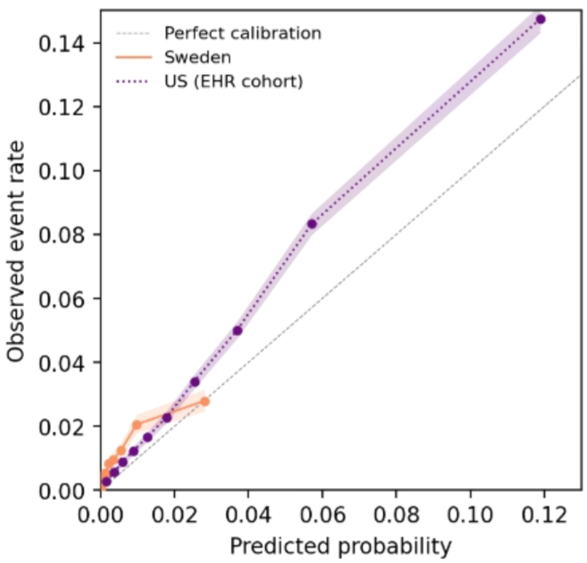

We think the main driver here is not over- or under-fitting of the algorithm—after all, the relationship of observed vs. predicted risk is similar for Sweden and the US—but rather large differences in the population risk distributions. Sweden is both lower-mean, and lower-variance, given general health and equal access to the health system. We confirm this visually using an overlayed histogram of the distribution of predicted risk in Sweden vs. the US (with each population normalized to 100% so heights reflect percent of ECGs rather than counts; binning is identical, with 500 equal-width bins spanning 0–0.20 risk predictions). The vertical dashed line marks the absolute high-risk threshold defined in the Swedish cohort. The US mean risk is far higher (0.029 vs. 0.005), which we expect from the higher base rate of VF/VT. But importantly, the US risk distribution is far wider (SD 0.041 vs. 0.009), supporting the idea of differences in distribution, not just mean. We include the figure, with a condensed version of this discussion, in Supplement III.C.

Overall, we think the absolute risk threshold is a good and conservative choice, and we really appreciate the comment. We could of course tailor the model much better to new populations if we did have distributional information, and we can adjust the risk threshold up or down depending on the capacity constraints of the health care system for follow-up (which also does not require population information). But overall, these results indicate that even without that information, the model is able to identify similarly risky high-risk groups in a truly “zero-shot” way.

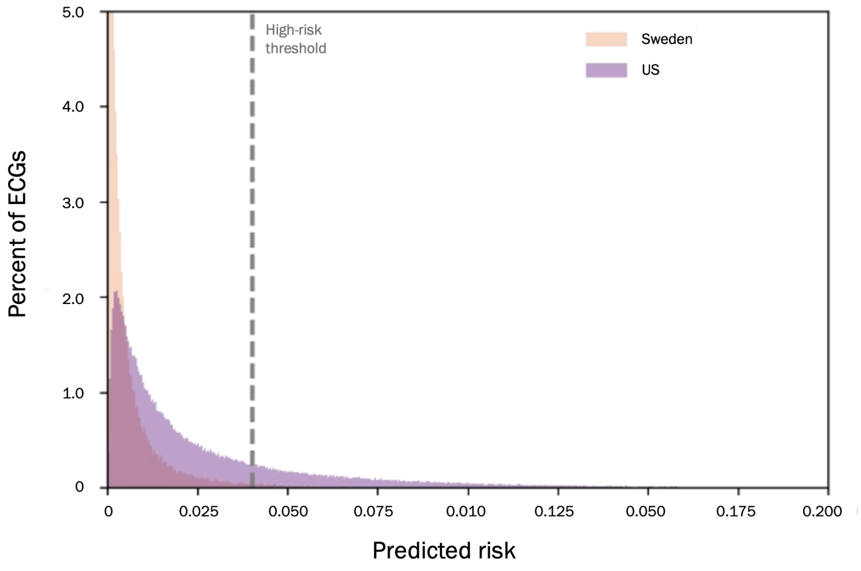

4. The analysis about VFVT on page 5 is interesting. The authors showed two (almost) monotonically increasing curves of VFVT in figure 2, and suggesting the model is predicting arrhythmic death. On the right side of figure 2a, the VFVT alone risk is not increasing so monotonically, but the VFVT plus SCD risk is rising sharply. Does it suggest the model picks more unpreventable non-arrhythmic death in the high-risk group? To complement this analysis, I would like a curve of the ratio between (VFVT+SCD) and VFVT. Assuming detected the ratio between recorded VFVT and all VFVT is a constant; the ratio between all VFVT and death certificates is also a constant, then we should see a flat curve of (VFVT+SCD)/VFVT.

This is an interesting question. It's certainly possible that the model is picking up on more unpreventable non-arrhythmic death in the high-risk group, and we now mention this possibility explicitly in the main text.

You are correct that the share of VF/VT cases (vs. the total sum of VF/VT+SCD) in Sweden is decreasing - here is the graph you suggest:

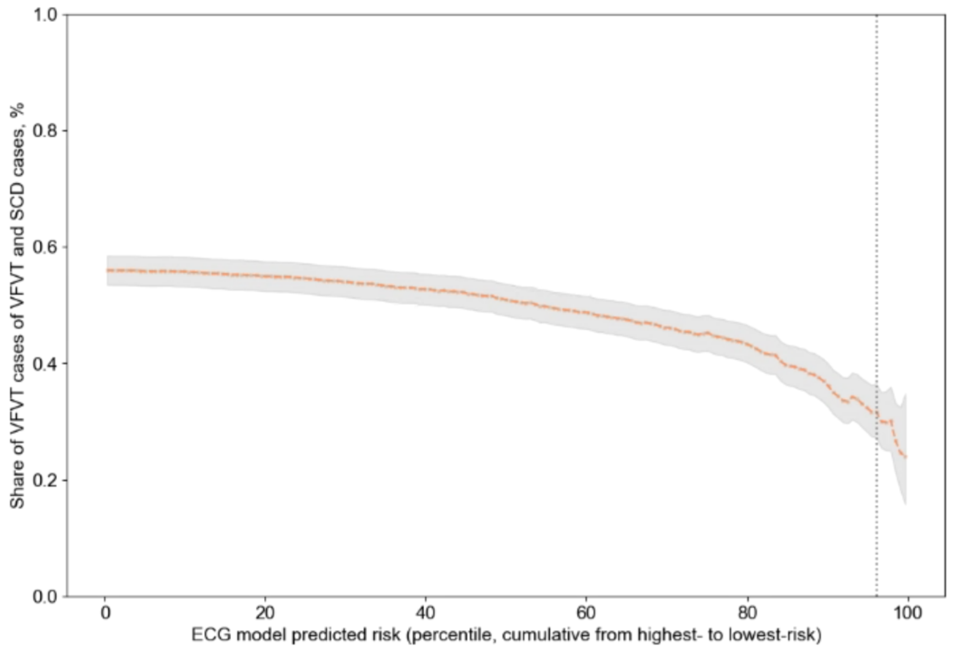

However, we have a more optimistic interpretation of this fact. One of the reasons that VF/VT is a complex validation metric is because it depends on measurement: a doctor needs to do an ECG (or order a Holter monitor, or place the patient on a cardiac monitor in the hospital, etc.), at the right time to catch the rhythm, before it self-resolves or kills the patient. So it's quite possible that there is more VF/VT than we observe in the high-risk patients, but it is not captured before they die. Indeed, because VF/VT and sudden cardiac death are correlated, there is likely considerable censoring of VF/VT in the highest-risk patients where death is more common.

Of course, if testing for VF/VT is constant across risk groups (as a fraction of all arrhythmic events, whether they lead to death or self-resolve), we would see the same share of VF/VT in the graph above, not a decrease. But even very small correlations between testing and risk can change this dramatically: if, for example, the high-risk cases are also more sudden, or more likely to kill the patient as opposed to self-resolving - which seems plausible - censoring would increase. A simple simulation shows this directly: in a population with Beta-distributed risk *r* of arrhythmia (similar to the empirical distribution of risk predictions) a doctor will test with some probability *t*, revealing VF/VT (*v*=1); otherwise the patient dies (*d*=1) and no VF/VT is recorded. Censoring is simulated by introducing a small negative correlation between testing *t* and risk *r*. We reproduce Figure 2 in this population below, left, and the graph you requested - share of VF/VT - on the right. With no correlation between *r* and *t*, risk increases exponentially (left: *k*=0, top line), and VF/VT share is flat (right: *k*=0, top line). But even small correlations (k = -0.025, corresponding to a ~10% decrease in testing in the top risk decile) flattens this into a straight line, and causes the share of VF/VT to slope down in the highest-risk groups, just as we see in the real graphs. So censoring is also very consistent with the data. A link to the Jupyter notebook is here if you want to play with the parameters and remake the graphs.

Simulation: VF/VT outcomes, varying correlation between testing and risk

Rate of VF/VT, high risk group

Share of VF/VT in high risk group

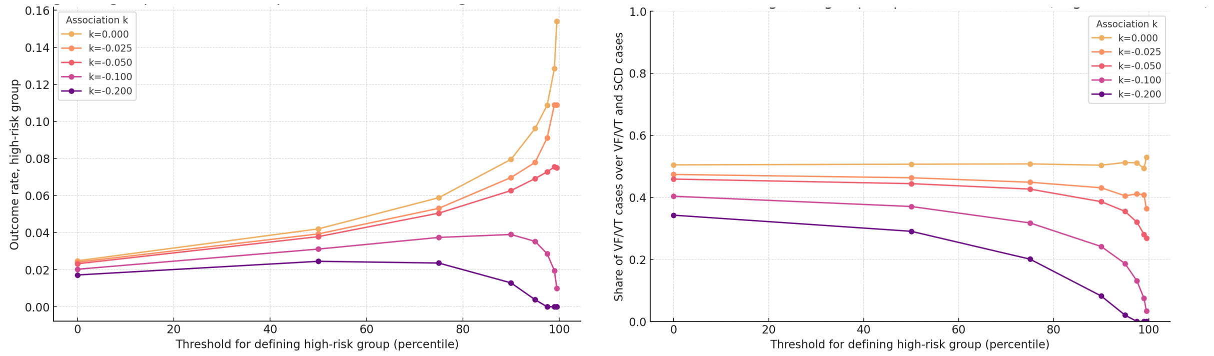

Another piece of circumstantial evidence is that the VF/VT graphs in the US look more exponential in risk. One reason for this is that - for better or worse - measurement of VF/VT happens far more often in the US's incentive-driven fee-for-service system, than it does in a national health system. As we note above, strong financial incentives drive testing for arrhythmias like VF/VT, because their presence increases reimbursement rates (they are "CC/MCC"'s - see here). This leads to far higher testing and documentation rates of VF/VT in the US. So in Sweden, the model could be (correctly) predicting that a high-risk patient will have VF/VT, but because that person is less likely to be tested or documented in Sweden, the model will look "wrong" in the Swedish data, decreasing PPV. By contrast, in the US, when a higher proportion of incident VF/VT is actually captured, the PPV of the model goes up. So overall, we think the US data supports our more optimistic interpretation of the fact you noted from Sweden - that is represents a lack of measurement for one reason or another, rather than the presence of a non-intervenable group.

As noted above, we transparently mention both possibilities in the text, so that the reader can draw her own conclusions. We also provide additional detail on censoring and the US vs Sweden comparison in a new Supplement section (III.C).

5. Re "We can then compare this AUC to the AUC for arrhythmic arrests above: if our model is specific to arrhythmia, it should perform worse on the placebo test; if our model inappropriately flags both arrhythmic and non-arrhythmic deaths as high risk, the statistics should be similar. Empirically, the non-arrhythmic AUC is significantly worse (p<0.001) than the arrhythmic AUC, indeed it approaches random guessing: 0.5853 (0.5446 - 0.6622).": Interesting analysis. I need confirm I understand correctly. This calculates two AUCs, one for arrhythmic, the other for non-arrhythmic. If so, what are the event rates for either of them? Following comment 2, I suggest reporting additional metrics, especially if the rates are different, because AUC is sensitive to event rates.

Yes, exactly - two AUCs, one for arrhythmic (vs. control), the other for non-arrhythmic (vs. control).

As noted above, we have added the additional metrics you request to Supplement III.

6. Re "This latter is the quantity of interest: it captures mortality differences in high-risk patients with defibrillators, relative to what we would expect based on (i) their high risk and (ii) the fact that they had a defibrillator, independently.": Very clear idea and interesting analysis.

Thank you!

7. Re Table 1: Why ICD increases risk for SCD but decreases risk for all-cause mortality? This counter intuitive results make me double this methodology and other results in this table.

This is a good question - we hadn't noticed that specific contrast. That said, we think there is a good explanation, related to selection bias rather than causal effects of ICD on the outcome. To summarize (and far more detail below): when doctors select which patients get ICDs, they are specifically trying to identify those who go on to have SCD, as opposed to all-cause mortality. This creates selection bias - i.e., a positive correlation between ICD and SCD; and this is more pronounced for the SCD regression than for the all-cause mortality regression. This results in a more positive coefficient on ICDs in the SCD-specific regressions, and a less positive (i.e., negative) coefficient in the all-cause mortality regressions.

Here is a bit of a longer explanation. In both the SCD regressions and the all-cause regressions, the coefficients capture the complex effects of three different forces:

(1) Selection bias: Doctors select patients for ICDs based on their perceived risk of SCD. Doctors have a lot of information about patients that are not fully captured by our risk predictions (based on either the ECG or LVEF), and some of that risk information is accurate. That results in a positive correlation (without causation) between ICDs and the outcome, conditional on the ECG or LVEF.

(2) Causation: of course, we believe that ICDs causally reduce mortality, which results in a negative correlation between ICDs and the outcome.

(3) High- vs. low-risk groups: Because we include both an ICD indicator variable, and an interaction term between the ICD variable and the high-risk variable (based on ECG or LVEF), the net correlation between ICDs and mortality - the offsetting effects of (1) and (2) - is going to be divided up between two coefficients:
   a. The ICD coefficient alone will capture the net correlation with the outcome among those who are not high risk (those the ECG model low-risk group, or those with preserved LVEF)
   b. The interaction term coefficient will capture the net correlation with the outcome among the high risk group (the ECG model high-risk group, or those with reduced LVEF).

Here’s how those forces can together translate into a more negative coefficient on ICD (alone) in the all-cause mortality regressions than the SCD regressions.

- In SCD regressions: We observe that the ICD (alone) coefficient is positive, which reflects the net balance of the two forces in the lower-risk group: (1) doctors are good at identifying risk factors for SCD that are *not captured* by the ECG-risk or LVEF (which we’re controlling for). These things are present in the low-risk group - by definition, they are not captured by ECG-risk or LVEF - and that drives a positive correlation between low-risk patients having an ICD and ultimately having SCD. The causal effect (2) is not big enough in this group to cancel it out, resulting in a positive coefficient.
- In all-cause mortality regressions: The key observation is that force (1) is going to be much smaller. When doctors choose which patients get ICDs, they are making the decision based on risk of SCD, *not* all cause mortality. That means ICDs will be *less correlated with all-cause mortality* than they are with SCD-specific mortality. So, unlike the positive correlation between having and ICD and the outcome we saw in the SCD regressions, here we will have a smaller correlation (closer to zero). If force (1) goes from positive to zero, it will make the total coefficient more negative.

One other thing that might have worried you is that the coefficient magnitude in the all-cause mortality regressions is much larger than in the SCD regressions. But the base rate of all-cause mortality is also much higher than SCD, because SCD is a subset of all-cause mortality: you can see this by comparing the absolute magnitude of the ICD coefficient to the intercept (a proxy for the base rate), and seeing they are about the same relative magnitude in the SCD and all-cause mortality regressions.

So overall, while we didn’t notice this and are very grateful that you pointed it out, after reflection we don’t think this should cause less confidence in the results. We have added a very condensed version of this explanation to a pre-existing footnote on interpreting coefficients, in the same section.

Finally, and stepping back from all these details, we want to highlight another reason to have confidence in this general analytic setup. When we use this setup to estimate the effect of defibrillators in those with reduced LVEF, we get a coefficient that is quite close to the causal effects shown in RCTs. So while it’s difficult to definitively say what’s going on with the control variables - they reflect a mix of selection bias and causation - the quantity of interest is quite close to our best guess about the truth, in the setting where the truth is known.

8. The missing S-wave morph in aVL, although has not been discussed much before, also correlates with left anterior–superior fascicle block and axis deviation. There are existing ECGs characteristics for this condition in different leads. How much additional information does the proposed biomarkers provide in predicting SCD?

We’ve added an analysis to Supplement V.G to answer this question, and mention the results in the main text as well. The results in the initial submission did control for frontal axis rotation

(which is the usual measure of LAFB; we also controlled for horizontal axis). But this is an interesting and important question, so we now show the frontal axis coefficient alongside the new features' coefficients directly in a new table, for easy comparison. This is below and in Supplement V.G.

There is a lot going on in the table (and more detail below), but to summarize: in the majority of regressions, both the new difference features and the LAFB feature are all significant, and the z-score analysis shows that they are of the same order of magnitude (in terms of how variation in the features correlates to variation in the outcome). So the new features are adding new, independent signal for predicting SCD, over an above (and about the same size as) LAFB.

More detail: The table uses Swedish data to compare the coefficients of the first- and second-difference features vs. frontal axis deviation, to investigate how much the new features add to the known feature of left anterior–superior fascicle block. Each row calculates the difference features in one ECG lead. Each pair of columns A and B show the coefficient of a difference feature (columns A) and the frontal axis feature (columns B) in one regression. All regressions control for (but do not show coefficients for) standard quantitative ECG features: heart rate, duration of P wave and QRS complex, frontal and horizontal QRS axis, QT interval (unnormalized and corrected), and an indicator for QTc>500ms. The left four columns use sudden cardiac death as the outcome, and the right four columns use VF/VT as the outcome. The top panel shows the results of a simple regression with all variables in their natural units; and the bottom panel converts the variables into standard deviation units (i.e., each predictor variable is converted to a z-score) in order to directly compare the magnitude of the new variables vs. frontal axis rotation.

	**SCD**				**VF/VT**			
	1st diff	Front axis‡	2nd diff	Front axis‡	1st diff	Front axis‡	2nd diff	Front axis‡
**Natural units**								
	-0.0242***	-0.0014*	-0.0103	-0.0016**	-0.0554***	-0.0059***	-0.0297**	-0.0062***
Lead I	(0.006)	(0.001)	(0.007)	(0.001)	(0.008)	(0.001)	(0.009)	(0.001)
	-0.0233**	-0.0016**	0.0335**	-0.0021***	-0.0697***	-0.0061***	-0.0074	-0.0066***
Lead II	(0.008)	(0.001)	(0.01)	(0.001)	(0.011)	(0.001)	(0.014)	(0.001)
	-0.0336***	-0.0012*	-0.0214***	-0.0016**	-0.0315***	-0.0060***	-0.0153*	-0.0064***
Lead III	(0.005)	(0.001)	(0.006)	(0.001)	(0.007)	(0.001)	(0.008)	(0.001)
	-0.0055	-0.0018**	0.061***	-0.0019***	-0.0584***	-0.0064***	-0.0125	-0.0066***
Lead aVR	(0.008)	(0.001)	(0.011)	(0.001)	(0.01)	(0.001)	(0.015)	(0.001)
	-0.0468***	-0.0017**	-0.0186	-0.0015**	-0.1395***	-0.0068***	-0.0616**	-0.0062***
Lead aVL	(0.012)	(0.001)	(0.015)	(0.001)	(0.017)	(0.001)	(0.019)	(0.001)
	-0.0248***	-0.0015**	-0.0113	-0.0017**	-0.0564***	-0.0058***	-0.0309**	-0.0061***
Lead aVF	(0.006)	(0.001)	(0.007)	(0.001)	(0.008)	(0.001)	(0.009)	(0.001)
	-0.023***	-0.0015**	-0.0175**	-0.0018**	-0.0257**	-0.0064***	-0.0078	-0.0067***
Lead V1	(0.006)	(0.001)	(0.007)	(0.001)	(0.007)	(0.001)	(0.009)	(0.001)

Lead V2	-0.003 (0.009)	-0.0018** (0.001)	-0.0083 (0.011)	-0.0018** (0.001)	-0.0204 (0.012)	-0.0066*** (0.001)	0.0003 (0.015)	-0.0067*** (0.001)
Lead V3	-0.0029 (0.008)	-0.0018** (0.001)	0.0283* (0.012)	-0.0018** (0.001)	-0.0076 (0.011)	-0.0067*** (0.001)	0.0144 (0.016)	-0.0067*** (0.001)
Lead V4	-0.0309*** (0.006)	-0.0013* (0.001)	-0.0137 (0.011)	-0.0016** (0.001)	-0.0305*** (0.009)	-0.0063*** (0.001)	-0.009 (0.014)	-0.0066*** (0.001)
Lead V5	-0.0433*** (0.007)	-0.0007 (0.001)	-0.0255* (0.01)	-0.0013* (0.001)	-0.0813*** (0.009)	-0.0050*** (0.001)	-0.0558*** (0.014)	-0.0060*** (0.001)
Lead V6	-0.0441*** (0.008)	-0.0011 (0.001)	-0.0208 (0.011)	-0.0013* (0.001)	-0.0823*** (0.011)	-0.0060*** (0.001)	-0.0338* (0.015)	-0.0065*** (0.001)
**SD units**†								
Lead I	-0.1022*** (0.025)	-0.0681* (0.027)	-0.0389 (0.025)	-0.0759** (0.027)	-0.2344*** (0.033)	-0.2802*** (0.036)	-0.1119** (0.034)	-0.2934*** (0.036)
Lead II	-0.076** (0.026)	-0.076** (0.027)	0.0869** (0.027)	-0.1002*** (0.027)	-0.227*** (0.035)	-0.2896*** (0.036)	-0.0191 (0.036)	-0.3142*** (0.036)
Lead III	-0.16*** (0.025)	-0.0559* (0.027)	-0.0889*** (0.024)	-0.0765** (0.027)	-0.15*** (0.033)	-0.2863*** (0.036)	-0.0638* (0.032)	-0.3075*** (0.036)
Lead aVR	-0.0188 (0.026)	-0.087** (0.027)	0.1535*** (0.028)	-0.0928*** (0.026)	-0.1991*** (0.035)	-0.3046*** (0.036)	-0.0314 (0.038)	-0.3132*** (0.036)
Lead aVL	-0.101*** (0.025)	-0.0818** (0.027)	-0.0348 (0.027)	-0.072** (0.027)	-0.2815*** (0.033)	-0.3249*** (0.036)	-0.1155** (0.037)	-0.2941*** (0.036)
Lead aVF	-0.1041*** (0.025)	-0.0711** (0.027)	-0.0421 (0.025)	-0.0787** (0.027)	-0.237*** (0.033)	-0.276*** (0.036)	-0.1151** (0.034)	-0.2891*** (0.036)
Lead V1	-0.1027*** (0.025)	-0.0699** (0.027)	-0.0649** (0.024)	-0.085** (0.026)	-0.1148** (0.033)	-0.3032*** (0.036)	-0.0288 (0.032)	-0.3205*** (0.036)
Lead V2	-0.0086 (0.026)	-0.0845** (0.027)	-0.0191 (0.026)	-0.0838** (0.027)	-0.0582 (0.035)	-0.3142*** (0.036)	0.0006 (0.035)	-0.3195*** (0.036)
Lead V3	-0.0085 (0.025)	-0.0851** (0.026)	0.0616* (0.026)	-0.0847** (0.026)	-0.0226 (0.034)	-0.3204*** (0.036)	0.0314 (0.036)	-0.3199*** (0.036)
Lead V4	-0.117*** (0.025)	-0.0613* (0.027)	-0.0325 (0.025)	-0.0749** (0.027)	-0.1156*** (0.033)	-0.2994*** (0.036)	-0.0213 (0.034)	-0.3146*** (0.036)
Lead V5	-0.166*** (0.026)	-0.0344 (0.027)	-0.0633* (0.025)	-0.0622* (0.027)	-0.3114*** (0.035)	-0.2406*** (0.037)	-0.1388*** (0.034)	-0.2881*** (0.037)
Lead V6	-0.1418*** (0.026)	-0.0515 (0.027)	-0.0485 (0.026)	-0.0638* (0.027)	-0.2642*** (0.036)	-0.2854*** (0.036)	-0.0786* (0.036)	-0.3096*** (0.036)

† Predictors were standardized and then divided by 100. To obtain per 1 SD effects, divide coefficients by 100.
‡ QRS Front Axis measurements were divided by 100.

Finally, we also now include in Supplement V.G a discussion of features beyond LAFB that relate to the ECG morphology the model discovers. For example, intrinsicoid deflection, increased time from QRS start to peak, has been linked to cardiac adverse events. However, the high-risk changes we identify are most apparent in the later parts of the QRS complex, not the early parts; and empirically, calculation of intrinsicoid deflection revealed that it is not increased in high-risk waveforms in our sample. Fragmented QRS morphology linked to prior MI

has also been described, but (because of the RSR' or notching pattern) this feature is likely linked to increased rather than decreased first and second derivatives of the QRS. Late potentials, low-level signals after the QRS complexes, are also related, but these are small-amplitude enough that they require special signal processing to observe, and are not visible on standard ECGs. Finally, QRS duration has been linked to sudden cardiac death; however, empirically the QRS interval is not linked to changes in the first- or second-differences. In any case, we control for QRS interval (and all other intervals) in the regression, so QRS duration has already been accounted for in the analysis.

9. I am disappointed that the model weights are not provided. I understand the original data cannot be made public, but I think the weights should be. The model is on ECG, and the model weights do not contain identifiable information.

It would be our preference to release the model weights as well. We are very committed to open science: as a concrete example, our Taiwan validation dataset is completely open access and hosted on a non-profit that two of the co-authors (ZO, SM) founded, Nightingale Open Science. The organization's mission is to make medical data accessible for non-profit research.

If we can be very frank, the primary concern is uncertainty regarding how European laws potentially governing the model will be applied. GDPR is relatively new and hasn't been tested much in the real world. This project represents a very, very unusual case where US researchers have been allowed to interact directly with (anonymized) patient data from Europe, which is likely to attract additional scrutiny on publication. This makes everyone, including us, quite nervous. We have taken great pains to carry out the work in the most secure and ethical way possible, and we believe everything is compliant with laws on both sides of the Atlantic. But the reality is that we are pushing outside of an established comfort zone for multiple reasons. We are hoping to reevaluate this question after the paper is published, after we can judge any reactions and (we hope) will be in a better position to make the case regarding the benefits of sharing. We hope this is at least a partial explanation for this suboptimal situation.

Referee #2 (Remarks on code availability):

It is hard to reproduce if model weights are not published.

We agree. We are investigating ways to satisfy our own commitment to open science while working around some of the logistical issues that arise from this particular research collaboration.

We do want to point out that one part of the paper can be reproduced, even pending the full model weights availability, which is the results concerning the first- and second-difference of the QRS waveform. These features are straightforward to calculate in any ECG, and we hope that this is one way in which replication can proceed in the short term.

Referee #3 (Remarks to the Author):

I co-reviewed this manuscript with one of the reviewers who provided the listed reports.

Thank you!
